# Well-Defined Aryl-Fe^II^ Complexes in Cross-Coupling and C–H Activation Processes

**DOI:** 10.1021/acs.organomet.1c00100

**Published:** 2021-03-09

**Authors:** Carla Magallón, Oriol Planas, Steven Roldán-Gómez, Josep M. Luis, Anna Company, Xavi Ribas

**Affiliations:** Institut de Química Computacional i Catàlisi (IQCC) and Departament de Química, Universitat de Girona, Campus Montilivi, Girona E-17003, Catalonia, Spain

## Abstract

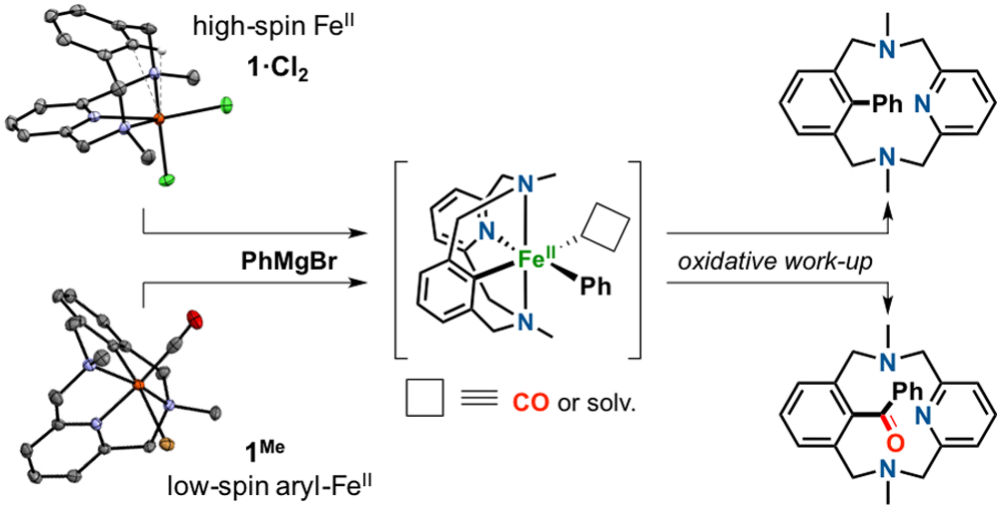

Herein we explore the intrinsic organometallic reactivity of iron embedded in a tetradentate N_3_C macrocyclic ligand scaffold that allows the stabilization of aryl-Fe species, which are key intermediates in Fe-catalyzed cross-coupling and C–H functionalization processes. This study covers C–H activation reactions using ^**Me**^**L**_**H**_ and FeCl_2_, biaryl C–C coupling product formation through reaction with Grignard reagents, and cross-coupling reactions using ^**Me**^**L**_**B**r_ or ^**H**^**L**_**Br**_ in combination with Fe^0^(CO)_5_. Synthesis under light irradiation and moderate heating (50 °C) affords the aryl-Fe^II^ complexes [Fe^II^(Br)(^**Me**^**L**)(CO)] (**1**^**Me**^) and [Fe^II^(^**H**^**L**)(CO)_2_]Br (**1**^**H**^). Exhaustive spectroscopic characterization of these rare low-spin diamagnetic species, including their crystal structures, allowed the investigation of their intrinsic reactivity.

Organoiron species have been invoked for a long time in cross-coupling transformations and C–H functionalization reactions for the formation of C–C products. Early in the 1970s, Kochi reported that simple FeCl_3_ could catalyze the methylation of haloalkenes with the use of alkyl Grignard reagents.^1,2^ Since then, many reports using cheap and nontoxic iron-based catalysts have appeared, highlighting the use of *N*-methylpyrrolidine (NMP) as an additive.^3−6^ More recently, the use of bisphosphine^7−9^ ligands or N-heterocyclic carbene^10−13^ ligands to tune the reactivity of the *in situ* formed organoiron species has allowed the development of a variety of cross-coupling C–C bond forming transformations.^14−22^ Many iron-catalyzed C–H functionalization protocols have also flourished in the past decade involving C_sp^2^_–H and C_sp^3^_–H activation, C–C bond forming reactions being the vast majority,^23−25^ although some examples of C–X bond formation (X = N, B, Si, O, halides) have also been reported.^26^ In the past decade, important advances in understanding the mechanism of these reactions relied on trapping relevant aryl or alkyl organoiron intermediate species.^18,27−31^ However, in the particular case of aryl-Fe species bearing directing groups (DG) attached to the substrate, detection of the organometallic species involved in cross-coupling or C–H activation catalysis has been quite elusive for a long time, and only scarce spectroscopic characterization has been reported. Either oxidative addition^32^ at Fe^0^ or σ-bond metathesis at Fe^II^ has been proposed to lead to the formation of aryl-Fe^II^ species (Scheme 1a).^32,33^ Concerted metalation–deprotonation (CMD) by Fe^II^ has also been proposed in some cases.^34^

**Scheme 1:**
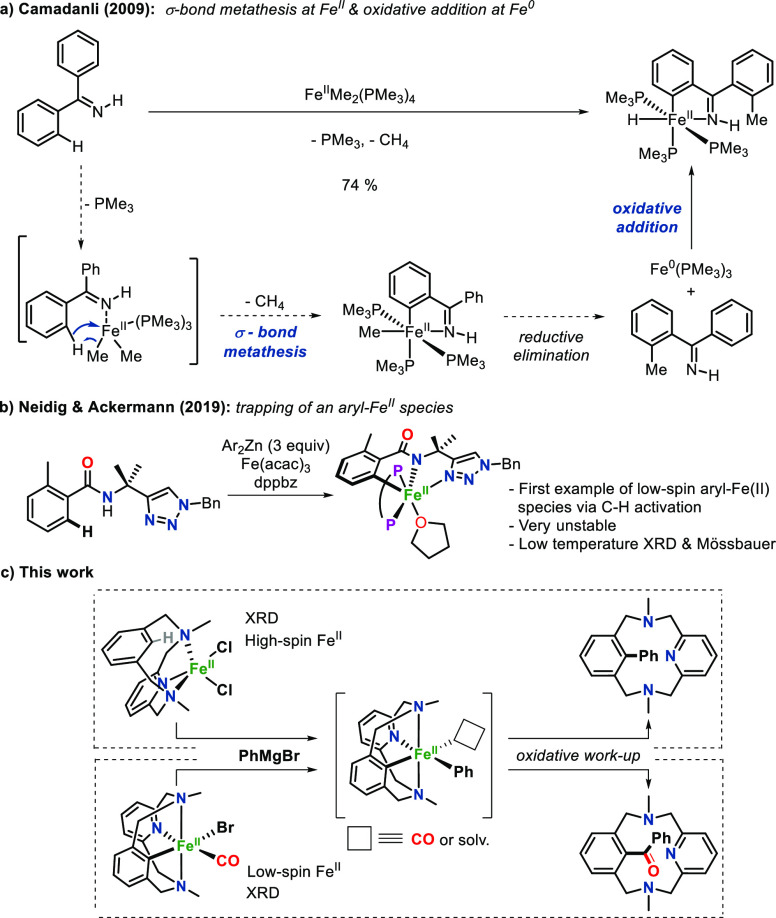
Relevant Examples of Iron-Mediated C–H Activation: (a) σ-Bond Metathesis at Fe^II^ and Oxidative Addition at Fe^0^, (b) Low-Spin Aryl-Fe^II^ Trapped at Low Temperature, and (c) Reactivity of Well-Defined Aryl-Fe^II^ Species formed via C–H Activation or Cross-Coupling to Undergo C–C Coupling (This Work)

Nakamura postulated a cyclometalated iron species as the active intermediate in an arene-containing substrate using the aminoquinoline (AQ) directing group, but actual spectroscopic data on this compound were not reported.^35,36^ This lack of mechanistic understanding stems from the metastable character of organoiron species together with their multiple geometries and oxidation and spin states. Recently Neidig reported a series of insightful publications in which the combination of advanced spectroscopic techniques such as Mössbauer spectroscopy and X-ray crystallography proved to be a successful strategy to identify catalytically relevant organoiron species.^37−39^

Moreover, there are very few examples of key low-spin aryl-Fe^II^ species stemming from C–H metalation in DG-bearing substrates. One of them was recently trapped by Neidig at very low temperatures using noncyclic substrates with an amide-triazole bidentate directing group (Scheme 1b).^40^ Another species was reported by Ackermann featuring a cyclometalated low-spin aryl-Fe^II^-hydride species ligated with a ketone DG and three PMe_3_ ligands.^41^ With regard to well-defined systems featuring aryl-halide oxidative addition processes, Nishiyama reported a low-spin aryl-Fe^II^ complex using a bisoxazoline aryl-Br pincer ligand and Fe^0^_2_(CO)_9_.^42^ Recently, Fout described the synthesis of an aryl-Fe^II^-hydride stabilized within a bis(carbene) pincer CCC ligand, but no reactivity of the aryl-Fe^II^ was reported.^43^ An alternative strategy to get access to well-defined aryl-Fe^II^ species consists of the use of macrocyclic aryl-X and aryl-H model substrates capable of stabilizing otherwise very reactive species. These size-tunable macrocyclic model substrates have been used by our group and others to stabilize square-planar aryl-Cu^III^,^44^ aryl-Ag^III^,^45^ and aryl-Ni^II^,^46^ as well as octahedral aryl-Co^III^ ^47^ and aryl-Mn^III^ species.^48^ Following this strategy, herein we report the reactivity of well-defined octahedral aryl-Fe^II^ species and their C–C cross-coupling reactivity with ArMgX reagents (Scheme 1c).

The model arene substrate ^**Me**^**L**_**H**_ was exposed to FeCl_2_ in CH_3_CN to obtain the coordination complex [Fe^II^(Cl)_2_(^**Me**^**L**_**H**_)] (**1·Cl**_**2**_) in 86% yield, which was isolated as a yellowish crystalline solid (Figure 1a). Paramagnetic ^1^H NMR spectroscopy clearly indicated a high-spin Fe^II^ species, which was confirmed by X-ray crystallography (Figure 1b). The Fe^II^ center featured a pentacoordinated distorted-square-pyramidal geometry (τ = 0.46)^49^ with long Fe–N distances (>2.1 Å). Noticeably, the sixth coordination site was occupied by an interaction with the inner aromatic C–H bond of ^**Me**^**L**_**H**_, which conformed to an incipient C_Ar_-H···Fe interaction (Figure S59). The analogous structure with bromides as counterions was also obtained (**1·Br**_**2**_, τ = 0.46; Figure 1b and Figure S60).

**Figure 1:**
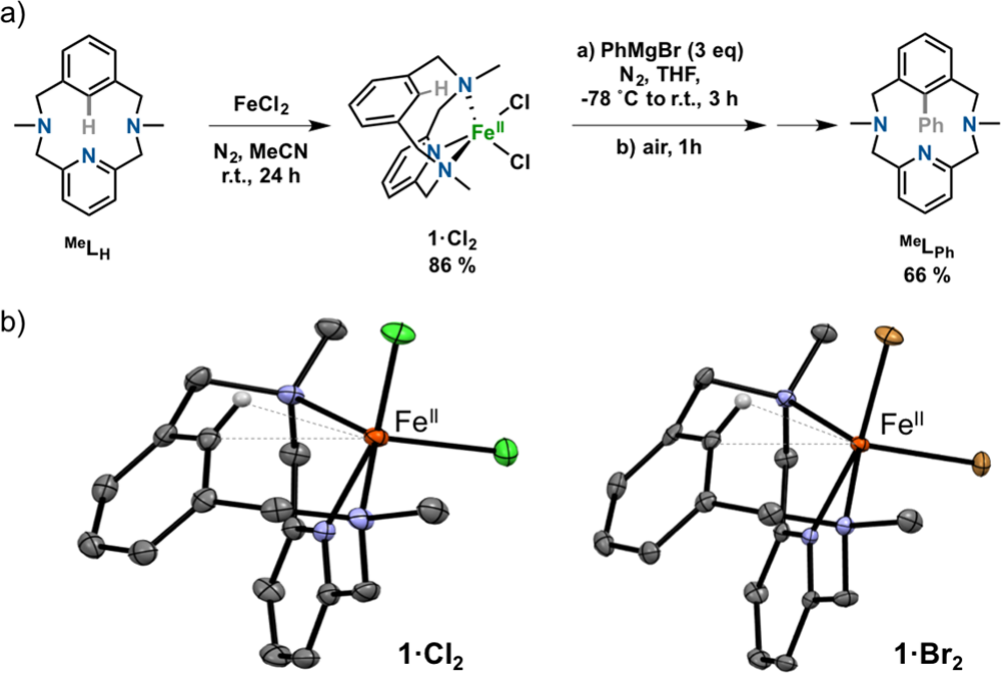
(a) Synthesis of the Fe^II^ complex **1·Cl**_**2**_ and subsequent reactivity with PhMgBr to obtain the biaryl C–C coupling product (^**Me**^**L**_**Ph**_). (b) Crystal structures of **1·Cl**_**2**_ and **1·Br**_**2**_ (ellipsoids set at 50% probability and H atoms removed for clarity, except for inner Ar–H).

These structures suggested that an octahedral geometry featuring an organometallic aryl–Fe bond was feasible, provided the C_Ar_–H activation could be executed. At this point we explored the reactivity of the complex **1·Cl**_**2**_ with PhMgBr Grignard reagent, seeking for a biaryl coupling product. By performing the reaction in THF at low temperature (−78 °C) for 1 h and warming up the mixture to room temperature for an additional 2 h, we obtained a 66% yield of the C_sp^2^_–C_sp^2^_ biaryl coupling product (^**Me**^**L**_**Ph**_) after workup under aerobic conditions (Figure 1a). The product was fully characterized by NMR and HR-ESI-MS (see the Supporting Information). Despite no organoiron species derived from C–H activation could be isolated, the intermediacy of an aryl-iron species is clearly inferred by the obtained coupling product. Whether C–H activation proceeds via σ-bond metathesis or concerted metalation–deprotonation (CMD) at the iron(II) center is difficult to establish.^32−34,50^ This prompted us to attempt another synthetic strategy to stabilize and isolate relevant aryl-iron species via aryl-halide oxidative addition at Fe^0^. Thus, we prepared aryl-Br ligand analogues (^**R**^**L**_**Br**_, R = H, Me, *t*Bu; Figure 2a) and reacted them with Fe^0^(CO)_5_. In the case of ^**Me**^**L**_**Br**_, upon overnight photoirradiation (254 nm) at 50 °C, the oxidative addition aryl-Fe^II^ product was obtained. The compound [Fe^II^(Br)(^**Me**^**L**)(CO)] (**1**^**Me**^, Figure 2a) was characterized as a low-spin Fe^II^ species and displayed diamagnetic NMR spectra (Figures S19–S23), which was directly related to the coordination of the strong-field carbonyl ligand. The crystal structure of **1**^**Me**^ confirmed a distorted-octahedral structure of the Fe^II^ center, featuring a short Fe–aryl bond (1.904(3) Å) and a long Fe–Br bond (2.571(2) Å) *trans* to the aryl moiety, with a CO ligand completing the coordination sphere (Figure 2b). This *trans* disposition indicated that the reaction must entail an aryl-Br oxidative addition concomitant with a *cis* to *trans* rearrangement.^51,52^ Indeed, the analogous [Fe^II^(^**H**^**L**)(CO)_2_]Br complex (**1**^**H**^) featured two CO ligands coordinated to the Fe^II^ center and a noncoordinating Br^–^ anion, clearly indicating that Br^–^ and CO ligands can easily exchange. Indeed, the *trans* effect of the aryl moiety is visualized by a longer Fe–CO bond *trans* to the aryl (1.837(3) Å) compared to the Fe–CO bond *trans* to the pyridine (1.759(3) Å). To evaluate the electronic effects of the tertiary amines, we also prepared the analogous complex [Fe^II^(^**tBu**^**L**)(CO)_2_]Br (**1**^**tBu**^) (Figure 2a), which was characterized by NMR and HR-ESI-MS.

**Figure 2:**
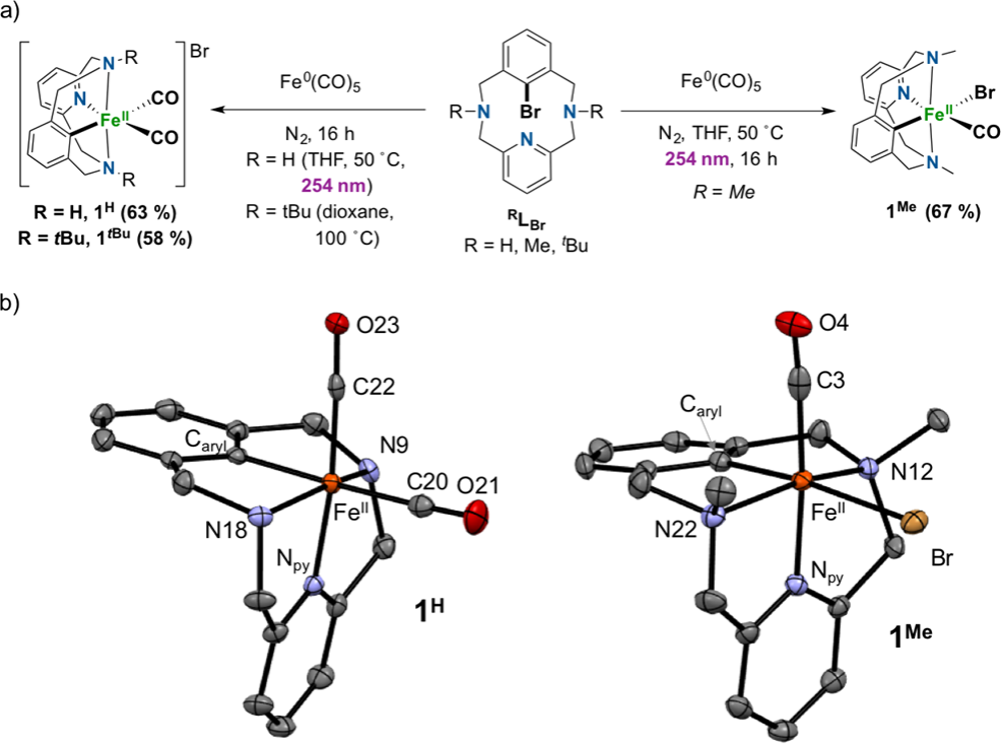
(a) Experimental conditions for the synthesis of **1**^**tBu**^, **1**^**Me**^, and **1**^**H**^ via aryl-Br oxidative addition at Fe^0^. (b) Crystal structures of **1**^**Me**^ and **1**^**H**^ (monocation shown) (ellipsoids set at 50% probability and H atoms removed for clarity). Selected bond distances (Å): for **1**^**H**^, Fe–C_aryl_ 1.925(2), Fe–N_py_ 1.928(2), Fe–N9 2.030(2), Fe–N18 2.034(2), Fe–C20 1.837(3), Fe–C22 1.759(3); for **1**^**Me**^, Fe–C_aryl_ 1.904(3), Fe–N_py_ 1.935(3), Fe–N12 2.095(3), Fe–N22 2.102(3), Fe–Br 2.571(2), Fe–C3 1.785(4).

At this point, we centered our efforts on investigating the intrinsic reactivity of [Fe^II^(Br)(^**Me**^**L**)(CO)] (**1**^**Me**^) as a reference compound for well-defined low-spin aryl-Fe^II^ species. In order to determine whether this species could be involved in the reaction of the complex **1·Cl**_**2**_ with the Grignard reagent, we reacted **1**^**Me**^ with PhMgBr under experimental conditions and workup analogous to those described above for **1·Cl**_**2**_, obtaining a relevant 38% yield of the ^**Me**^**L**_**COPh**_ product (Figure 3, top). NMR and HR-ESI-MS confirmed the nature of the coupling product, which stemmed from a putative [Fe^II^(^**Me**^**L**)(Ph)(CO)] (**1**^**Me**^**-Ph**) followed by a CO migratory insertion and reductive elimination to form the aryl–COPh bond in ^**Me**^**L**_**COPh**_.

**Figure 3:**
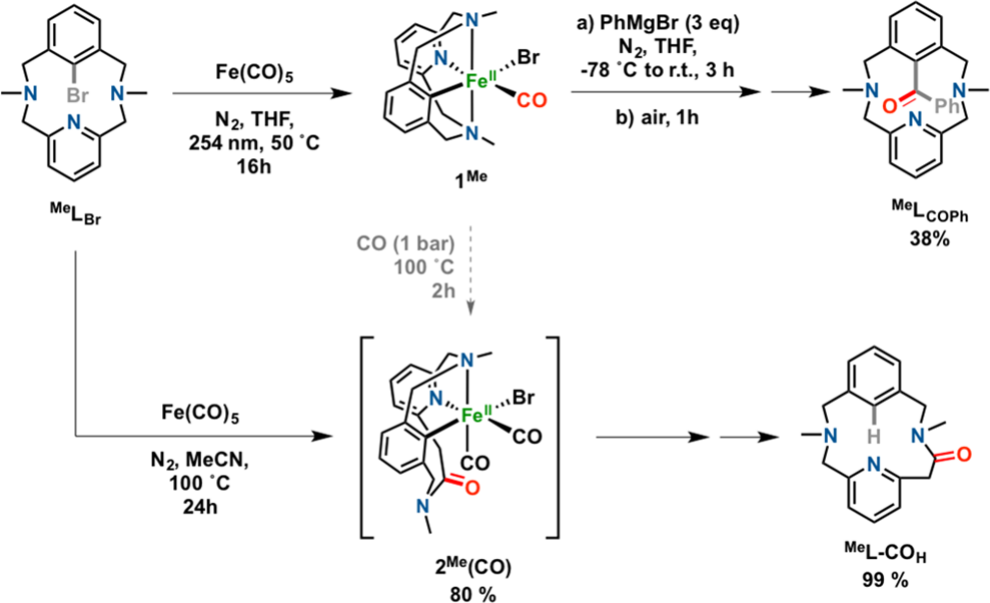
Synthesis of ^**Me**^**L**_**COPh**_ from well-defined aryl-Fe^II^ (top) and synthesis of ^**Me**^**L-CO**_**H**_ from ^**Me**^**L**_**Br**_ via **2**^**Me**^**(CO)** in an unprecedented amine-to-amide transformation (bottom).

It is worth noting here that the products ^**Me**^**L**_**Ph**_ (derived from **1·Cl**_**2**_) and ^**Me**^**L**_**COPh**_ (derived from **1**^**Me**^) are obtained presumably via exposure of [Fe^II^(^**Me**^**L**)(Ph)] and [Fe^II^(^**Me**^**L**)(Ph)(CO)] to O_2_ (or air) and a subsequent acid/base workup. An evident change in color (UV–vis monitoring, Figures S1 and S2) from dark green to reddish brown was observed upon contact with air, suggesting an oxidation to an Fe^III^ species that triggered the C–C reductive elimination, as reported in other examples.^26,53^ Despite cryo-MS analysis at −40 °C of the mixture immediately after exposure to O_2_, the decay was so fast that only the final coupling product ^**Me**^**L**_**Ph**_ was detected as a single peak in the mass spectrum (Figure S2b). Moreover, when the crude mixture containing [Fe^II^(^**Me**^**L**)(Ph)] was quenched with HCl prior to air exposure, ^**Me**^**L**_**H**_ was solely obtained (85%) with no signs of biaryl coupling. Finally, since 1,2- dichloroisobutane (DCIB) is generally used as an oxidant in Fe-catalyzed C–H activations,^26^ the addition of 2 equiv of DCIB under N_2_ to the green species [Fe^II^(^**Me**^**L**)(Ph)] afforded ^**Me**^**L**_**Ph**_ in 45% yield, a value slightly lower than that with O_2_ exposure (66%) (section 7.3 in the Supporting Information). Interestingly, DCIB addition at the beginning of the reaction only afforded a 9% yield of ^**Me**^**L**_**Ph**_, suggesting that oxidation to Fe^III^ at the initial stages is detrimental to the observed chemistry. In line with the latter, catalytic attempts have been unfruitful.

On the basis of all these experimental observations, feasible mechanistic proposals are outlined in Figure 4a for the synthesis of ^**Me**^**L**_**Ph**_ and in Figure 4b for the synthesis of ^**Me**^**L**_**COPh**_. The reaction of **1·Cl**_**2**_ with PhMgBr (Figure 4a) affords species **A**, which undergoes C–H activation, presumably via σ-bond metathesis, to give species **B**. A second equivalent of the Grignard reagent generates species **C**, which undergoes oxidative reductive elimination via **C**^**+**^ upon exposure to O_2_. With regard to the reactivity of **1**^**Me**^ with PhMgBr (Figure 4b), first the Br^–^ ligand is exchanged by Ph^–^ to afford **D**, and then a CO migratory insertion occurs to give **E-1** or **E-2**. Both species would form the final product ^**Me**^**L**_**COPh**_ via reductive elimination. To discern between the two possibilities, the crude compound was treated with HCl(aq) prior to air exposure, and ^**Me**^**L**_**H**_ was obtained as the product in 95% yield. This supports the idea that **E-1** is the most plausible intermediate, which is backed by DFT studies (Gibbs energies with respect to **D** are 6.19 kcal/mol for **E-1** and 9.72 kcal/mol for **E-2**; Figure 4b and the Supporting Information).

**Figure 4:**
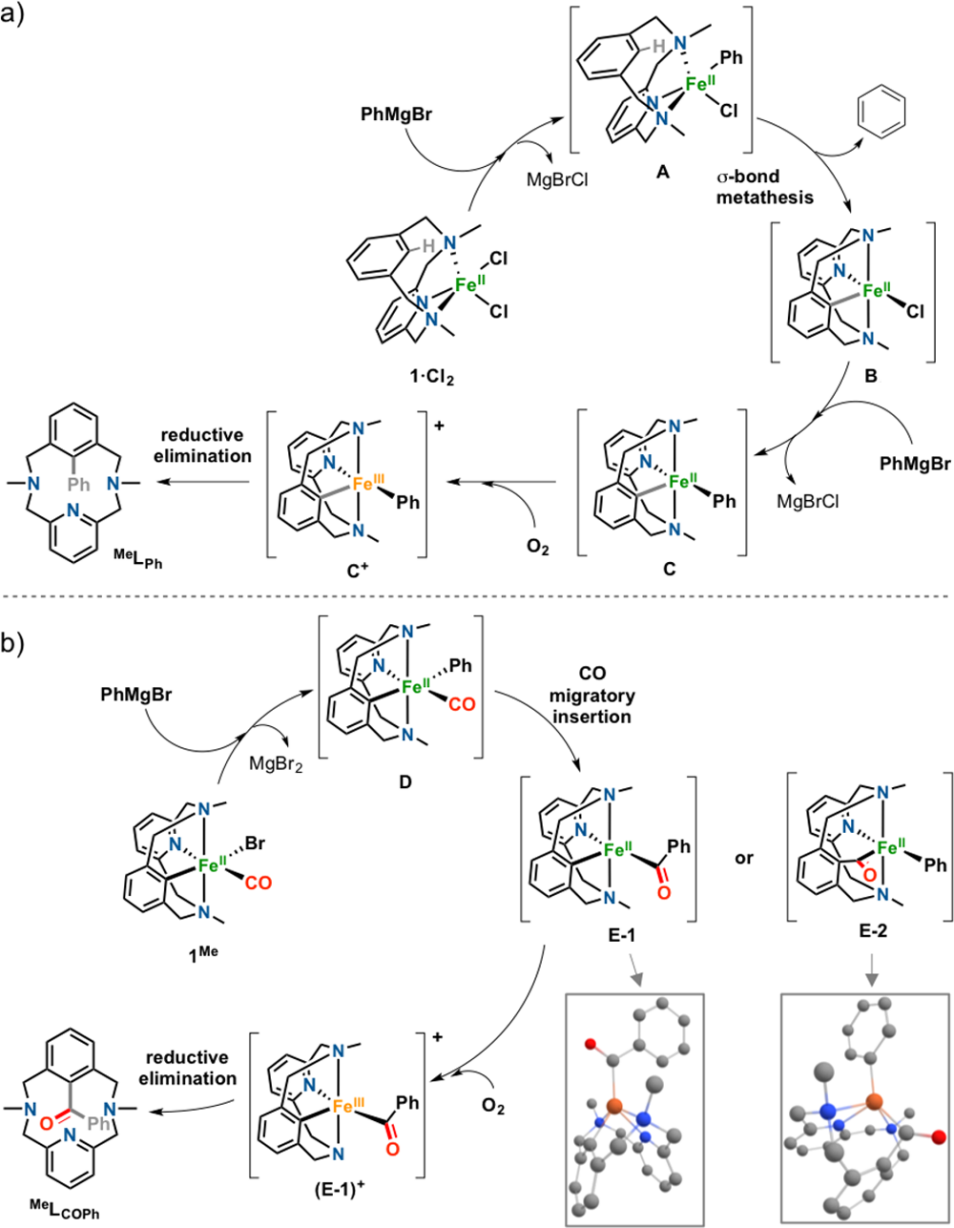
(a) Proposed mechanism for the synthesis of ^**Me**^**L**_**Ph**_ via Fe^II^-mediated C–H activation. (b) Proposed mechanism for the synthesis of ^**Me**^**L**_**COPh**_ via the reaction of **1**^**Me**^ with PhMgBr (**E-1** and **E-2** quintuplet DFT optimized structures shown as insets; see the Supporting Information).

While exploring the reactivity of ^**Me**^**L**_**Br**_ with Fe(CO)_5_, we also performed the reaction under thermal conditions (100 °C) instead of via photoirradiation (Figure 3, bottom). Strikingly, the nature of the low-spin Fe^II^ complex **2**^**Me**^**(CO)** obtained after 24 h was completely unexpected. A detailed diamagnetic 1D/2D NMR and FT-IR characterization concluded that a formal CO insertion occurred by amine to amide conversion at a pyridine-benzylic position, still holding the organoiron aryl-Fe^II^ moiety: i.e., [Fe^II^(Br)(^**Me**^**L-CO**)(CO)_2_] (**2**^**Me**^**(CO)**) (Figure 3). ^13^C NMR integration of the coordinated CO signal and the lack of a HR-ESI-MS peak clearly points toward the coordination of two CO and one Br^–^ ligand to the Fe^II^ center, leaving the amide moiety uncoordinated. The amide moiety was corroborated upon protodemetalation, affording ^**Me**^**L-CO**_**H**_ as the resulting macrocyclic compound (see the Supporting Information for characterization). To our knowledge, carbonylation into the ligand backbone to transform a tertiary amine to a tertiary amide is unprecedented and is reminiscent of an unreported inverse Curtius-like rearrangement.^54^ Although it is not the same transformation, Cantat recently reported the iron-catalyzed amine to amide transformation of an *N*,*N*-dimethylaniline substrate by taking advantage of the acylation of a tertiary amine followed by the extrusion of Me^+^ as MeI.^55^ Also, the participation of Fe in Curtius-like rearrangements has only a few precedents, such as the work from Xia, forming isocyanates from hydroxamates through an Fe^II^-nitrenoid complex.^56^

In order to gain insight into the mechanism of this unprecedented reactivity, the well-defined **1**^**Me**^ complex was heated under a CO atmosphere (1 bar). The reaction was monitored by ^1^H NMR, and formation of **2**^**Me**^**(CO)** was observed (14%) just after 2 h, together with the starting **1**^**Me**^ and protodemetalation byproduct (^**Me**^**L**_**H**_), thus suggesting that aryl-Br oxidative addition at Fe^0^ takes place prior to the amine to amide conversion. Also, the nature of the tertiary amine is crucial, since a *t*Bu-N-substituted ligand (^**tBu**^**L**_**Br**_) did not undergo the amine to amide transformation, whereas ^**H**^**L**_**Br**_ afforded ^**H**^**L-CO**_**H**_ in a sluggish manner (section 8 in the Supporting Information).

In conclusion, model macrocyclic aryl-Fe^II^ species have been studied in detail by taking advantage of the stabilizing effect imposed by the macrocyclic N_3_C-type ligands ^**X**^**L**_**Y**_ (X = H, Me; Y = H, Br). The system affords the C–C biaryl cross-coupling products through C–H activation at a Fe^II^ complex using ArMgX reagents and the phenylcarbonylation cross-coupling products when well-defined aryl-Fe^II^ species are used, featuring C–C coupling with Grignard reagents, concomitantly with CO insertion. Furthermore, the overstabilized **1**^**Me**^ species undergoes at high temperatures an unprecedented CO insertion–carbonylation into the tertiary amine ligand backbone, rendering a tertiary amide quantitatively. Such model aryl-Fe^II^ complexes provide a neat mechanistic picture for C–H arylation and cross-coupling reactions that should inspire others in the design of improved Fe-catalyzed bond forming transformations.

## Supporting Information Available

Spectroscopic characterization of all compounds, crystallographic data for **1·Cl**_**2**_, **1·Br**_**2**_, **1**^**Me**^, and **1**^**H**^, and DFT results for **D**, **E-1**, and **E-2** (PDF)

## Accession Codes

CCDC 2046155–2046158 contain the supplementary crystallographic data for this paper. These data can be obtained free of charge via www.ccdc.cam.ac.uk/data_request/cif, or by emailing data_request@ccdc.cam.ac.uk, or by contacting The Cambridge Crystallographic Data Centre, 12 Union Road, Cambridge CB2 1EZ, UK; fax: +44 1223 336033.
