# Supplementary material for: Well-Defined Aryl-FeII Complexes in Cross-Coupling and C–H Activation Processes
Source: Organometallics. 2021 Mar 9;40(9):1195–200. doi: 10.1021/acs.organomet.1c00100 (PMC9490821; doi:10.1021/acs.organomet.1c00100)
Supplement: Supplementary file 1 — om1c00100_si_001.pdf [file om1c00100_si_001.pdf]

Supporting Information for

Well-Defined Aryl-Fe^{II} Complexes in Cross-Coupling and C-H Activation Processes

Carla Magallon, Oriol Planas, Steven Roldán-Gómez, Josep M. Luis, Anna Company,* Xavi Ribas*

†Institut de Química Computacional i Catàlisi (IQCC) and Departament de Química, Universitat de Girona, Campus Montilivi, Girona, E-17003, Catalonia, Spain

Table of contents

1. General considerations **S1**
2. Synthesis of ligand $^{Me}L_{Br}$ **S2**
2.1. Synthesis of 2,6-bis(azidomethyl)pyridine (i) S2
2.2. Synthesis of 2,6-bis(aminomethyl)pyridine (ii) S3
2.3. Synthesis of 2,6-bis(tosylaminomethyl)pyridine (iii) S3
2.4. Synthesis of $^{Ts}L_{Br}$ S4
2.5. Synthesis of $^{H}L_{Br}$ S4
2.6. Synthesis of $^{Me}L_{Br}$ S5
3. Synthesis of Iron(II) complexes: $1{\cdot}Cl_2$ and $1{\cdot}Br_2$ **S5**
4. Reactivity of $1{\cdot}Cl_2$ towards PhMgBr reagent **S6**
5. Synthesis of Aryl-Iron(II) complexes **S7**
5.1. Synthesis of 1^{Me} S7
5.2. Synthesis of 1^{H} S7
5.3. Synthesis of 1^{tBu} S8
6. Reactivity of 1^{Me} with phenyl Grignard (PhMgBr) **S8**
7. Mechanistic insights **S9**
7.1. UV-vis monitoring of $1{\cdot}Cl_2$ with PhMgBr S9
7.2. Inert atmosphere work-up for $1{\cdot}Cl_2$ and 1^{Me} towards phenyl Grignard S12
7.3. Use of DCIB as oxidant for the reaction of $1{\cdot}Cl_2$ with PhMgBr S13
7.4. Use of CO atmosphere for the reaction of $1{\cdot}Cl_2$ with PhMgBr S13
7.5. Reaction of 1^{Me} with PhMgBr under photoirradiation (254 nm) S14
7.6. Attempts to isolate species C' ([Fe^{II}(MeL)(Ph)(PPh_3)]) S14
8. Amine-to-amide CO insertion reactivity **S15**
8.1. Synthesis of $2^{Me}(CO)_H$ and $^{Me}L\text{-}CO_H$ S15
8.2. Mechanistic insights S16
8.3. Amine-to-amide CO insertion reactivity using $^{H}L_{Br}$ S16
9. Spectroscopic characterization **S18**
10. X-ray Diffraction Analysis **S46**
11. DFT Modelling **S50**
11.1. Computational details S50
11.2. Relative electronic and Gibbs energy values of D, E-1 and E-2 ...... S50
11.3. XYZ coordinates of DFT optimized structures S50
12. References **S51**

1. General considerations

Materials and methods

All reagents and solvents were purchased from Sigma Aldrich, Fisher Scientific or Fluorochem and used without further purification. NMR data concerning product identity were collected with a Bruker Ultrashield AVANCE III400 and a Bruker Ultrashield ASCEND Nanobay 400MHz (Serveis Tècnics, Universitat de Girona) spectrometers ($CDCl_3$, THF-d_8, CD_3CN and DMSO-d_6) and calibrated relative the residual protons of the solvent. All NMR experiments (^1H, $^{13}C^1$, COSY, HSQC, HMBC, NOESY and TOCSY) were recorded and processed using standard parameters and no more details are given, unless otherwise stated. Quantification of reaction yields through integration of peaks was performed using an internal standard (1,3,5-trimethoxybenzene). Preparation and handling of air-sensitive materials were carried out in a N_2 drybox with O_2 and H_2O concentrations < 1 ppm. High resolution mass spectra (HRMS) were recorded on a Bruker MicrOTOF-Q IITM instrument using ESI as ionization source or CMS (cryospray ionization, for low temperature experiments) at Serveis Tècnics University of Girona. IR Spectra (FTIR) were recorded on a FT-IR Alpha spectrometer from Bruker with a PLATINUM-ATR attachment using OPUS software to process the data. UV-vis spectroscopy was performed with an Agilent 8453 UV-vis spectrophotometer with 1 cm quartz cells. Low temperature control was achieved with a cryostat from Unisoku Scientific Instruments, Japan. Monocrystal X-Ray diffraction was performed with a Bruker D8 QUEST ECO diffractometer. Ligands $^{Me}\mathbf{L}_H$ [2] and $^{tBu}\mathbf{L}_{Br}$ [3-4] were synthesized following previously described procedures. For $^{Me}\mathbf{L}_{Br}$ ligand, the synthesis was carried out following a slightly modified previously reported procedure.[2, 5-7]

2. Synthesis of ligand $^{Me}L_{Br}$

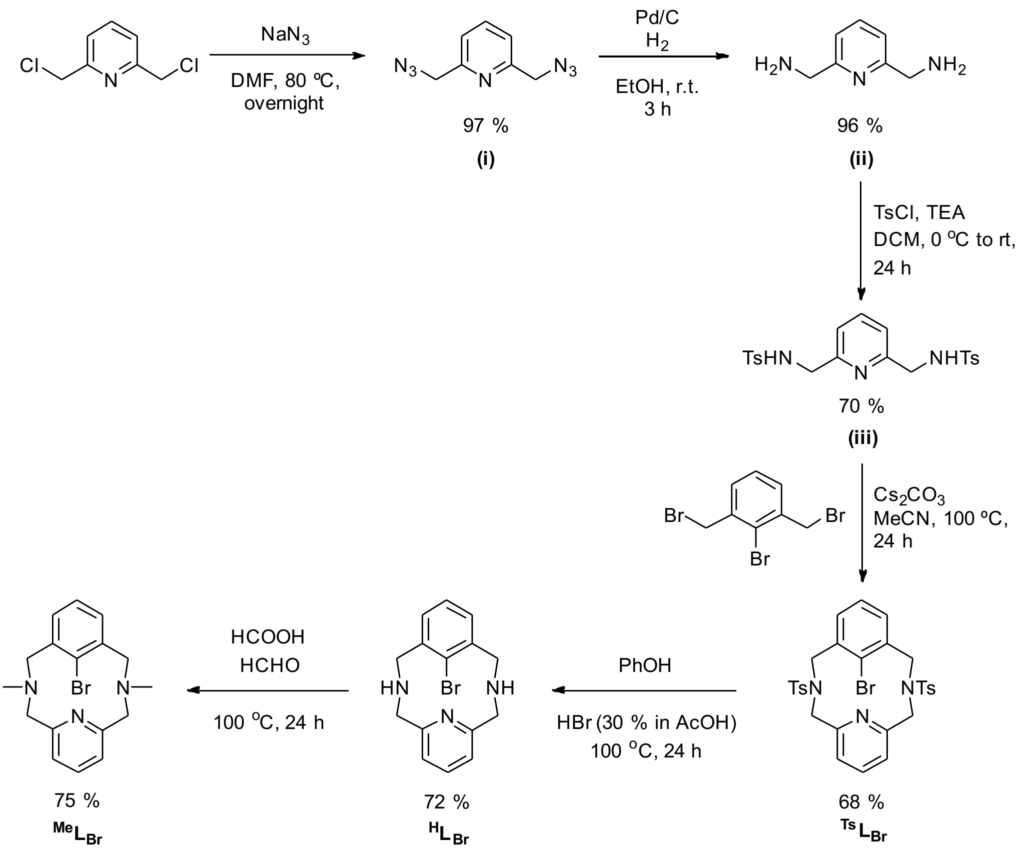

Scheme S1. Synthesis protocol for $^{Me}L_{Br}$ macrocyclic ligand.

2.1. Synthesis of 2,6-bis(azidomethyl)pyridine (i)

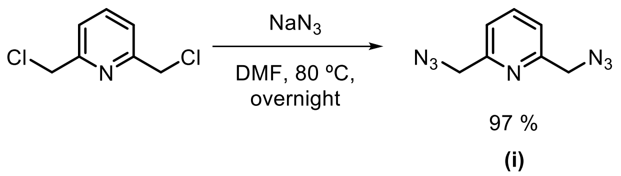

Scheme S2. Synthesis of 2,6-bis(azidomethyl)pyridine (**i**).

Sodium azide (9.59 g, 146.0 mmol) and 2,6-bis(chloromethyl)pyridine (2.5 g, 14.0 mmol), were dissolved in DMF (130 ml) and refluxed overnight at 80 °C. After that, the solvent was removed under reduced pressure, then the residue was extracted using water (4x60 ml) and EtOAc (100 ml). The organic layer was washed with brine (3x60 ml), dried over $MgSO_4$, filtered and the solvent was removed under vacuum to yield a colorless oil corresponding to 2,6-bis(azidomethyl)pyridine (**i**) (2.56 g, 13.5 mmol, 97 %).

^1H-NMR ($CDCl_3$, 400 MHz, 298 K) δ (ppm): 7.75 (t, *J* = 8 Hz, 1 H**a**), 7.30 (d, *J* = 8 Hz, 2 H**b**), 4.48 (s, 4 H**c**).

2.2. Synthesis of 2,6-bis(aminomethyl)pyridine (ii)

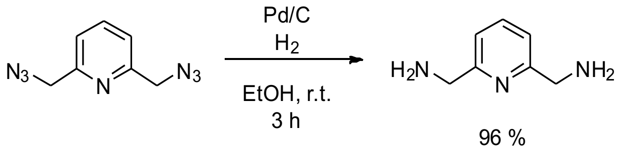

(i) **(ii)**

Scheme S3. Synthesis of 2,6-bis(aminomethyl)pyridine (**ii**).

Under N_2, a 1 L round flask was charged with 2,6-bis(azidomethyl)pyridine (2.50 g, 13.2 mmol) and Pd/C (0.25 g, 10%) and then diluted in ethanol (280 ml). After that, the reaction was purged with H_2 to remove N_2. The reaction was left stirring at room temperature for 3 hours under a H_2 atmosphere. The reaction solution was filtered using a celite® pad and rinsed with ethanol (the solid was discarded) and the filtrates were dried under reduced pressure to yield an orange oil corresponding to 2,6-bis(aminomethyl)pyridine (**ii**) (1.75 g, 12.7 mmol, 96%).

^{1}H-NMR (CD_3OD, 400 MHz, 298 K) δ (ppm): 7.75 (t, *J* = 8 Hz, 1 H**a**), 7.28 (d, *J* = 8 Hz, 2 H**b**), 3.92 (s, 4 H**c**).

2.3. Synthesis of 2,6-bis(tosylaminomethyl)pyridine (iii)

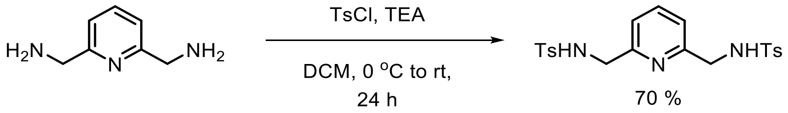

(ii) **(iii)**

Scheme S4. Synthesis of 2,6-bis(tosylaminomethyl)pyridine (**iii**).

Triethylamine (4.0 ml, 28.3 mmol) and 2,6-bis(aminomethyl)pyridine (1.85 g, 13.5 mmol) were dissolved in DCM (75 ml) and chilled to 0 °C. To this solution, TsCl (5.56 g, 28.6 mmol) diluted in DCM (100 ml) was added dropwise. During addition, the reaction mixture was vigorously stirred and maintained at 0 °C in an ice-bath. Upon completion of the addition, the reaction mixture was left stirring for an additional 24 hours allowing the solution to attain room temperature. The organic layer was washed with water and brine (2x175 ml each), dried with anhydrous $MgSO_4$, and solvent was removed under reduced pressure to yield a brownish oil. The crude product was purified by silica gel column chromatography (DCM:EtOAc, 95:5) to obtain 2,6-bis(tosylmethylamine)pyridine (**iii**) as white powder (4.21 g, 9.4 mmol, 70 %).

^{1}H-NMR ($CDCl_3$, 400 MHz, 298 K) δ (ppm): 7.72 (d, *J* = 12 Hz, 4 H**d**), 7.53 (t, *J* = 8 Hz, 1 H**a**), 7.25 (d, *J* = 12 Hz, 4 H**e**), 7.06 (d, *J* = 8 Hz, 2 H**b**), 5.50 (t, *J* = 6 Hz, 2 **N**H), 4.17 (d, *J* = 4 Hz, 4 H**c**), 2.41 (s, 6 H**f**).

2.4. Synthesis of $^{Ts}L_{Br}$

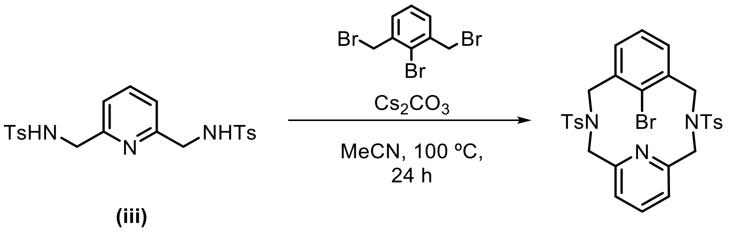

68 %
$^{Ts}L_{Br}$

Scheme S5. Synthesis of ^{Ts}**L**$_{Br}$.

Cs_2CO_3 (4.16 g, 12.6 mmol) and 2,6-bis(tosylaminomethyl)pyridine (2.60 g, 5.83 mmol) were dissolved in MeCN (150 ml) in a 500 ml 2-necked round-bottom flask. Once the mixture started refluxing, then a solution of 2-bromo-1,3-bis(bromomethyl)benzene (2.19 g, 6.25 mmol) in MeCN (100 ml) was slowly added dropwise. After heating at 100 ºC for 24 hours, the crude was cooled down to room temperature and filtered. The filtrates were evaporated under reduced pressure and the resulting pale-brown solid was purified by recrystallization in $CHCl_3$:EtOH (1:3) in the freezer. ^{Ts}**L**$_{Br}$ (2.5 g, 3.99 mmol, 68 % yield) was obtained.

^{1}H-NMR ($CDCl_3$, 400 MHz, 298 K) δ (ppm): 7.79 (d, *J* = 8 Hz, 4 H**d**), 7.39 (d, *J* = 8 Hz, 4 H**e**), 7.37 (t, *J* = 8 Hz, 1 Hi), 7.21 (d, *J* = 8 Hz, 2 H**h**), 7.17 (d, *J* = 8 Hz, 2 H**b**), 6.86 (t, *J* = 8 Hz, 1 H**a**), 4.88 (d, *J* = 20 Hz, 2 H**g**), 4.83 (d, *J* = 20 Hz, 2 H**g**), 4.49 (d, *J* = 16 Hz, 2 H**c**), 3.92 (d, *J* = 16 Hz, 2 H**c**), 2.48 (s, 6 H**f**).

2.5. Synthesis of $^{H}L_{Br}$

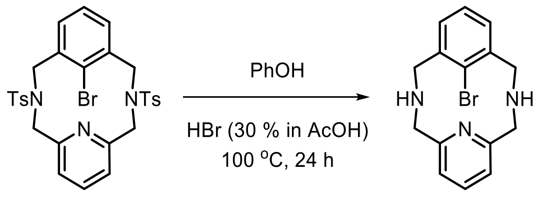

72 %
$^{Ts}L_{Br}$
$^{H}L_{Br}$

Scheme S6. Synthesis of ^{H}**L**$_{Br}$.

$^{Ts}L_{Br}$ (2.10 g, 3.35 mmol) and phenol (7.88 g, 82.9 mmol) were added as solid to a 250 ml round-bottom flask, then HBr (30 wt % in AcOH, 100 ml) was added, the resulting solution was stirred and heat up to 100 ºC for 24 hours. The crude was concentrated as much as possible and then 40 ml of H_2O were added. Extractions using $CHCl_3$ (3 x 40 ml) were performed. The organic layer was discarded, and the aqueous layer was basified with 50 % NaOH (~40 ml) until pH 14. After that, extractions with $CHCl_3$ (3 x 40 ml) were done again. The organic layer was then dried with $MgSO_4$, filtered and the solvent removed under reduced pressure. ^{H}**L**$_{Br}$ (0.77 g, 2.42 mmol, 72 % yield) was obtained as a white solid.

^{1}H-NMR ($CDCl_3$, 400 MHz, 298 K) δ (ppm): 7.20 (t, *J* = 8 Hz, 1H**f**), 6.62 – 6.54 (m, 5H**a,b,e**), 4.60 (d, *J* = 16 Hz, 2H**d**), 4.22 (d, *J* = 16 Hz, 2H**d**), 3.81 (d, *J* = 16 Hz, 2H**c**), 3.58 (d, *J* = 16 Hz, 2H**c**), 1.66 (br.s, **NH**).

2.6. Synthesis of $^{Me}L_{Br}$

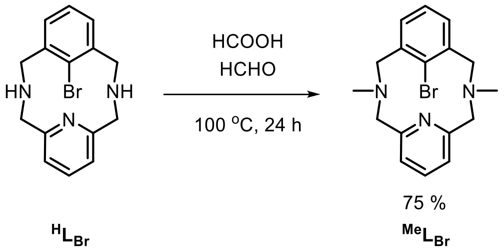

Scheme S7. Synthesis of ^{Me}**L**$_{Br}$.

$^{H}L_{Br}$ (770 mg, 2.42 mmol), formaldehyde (58 ml, 774 mmol) and formic acid (10 ml, 242 mmol) were mixed in a 100 ml round-bottom flask and heat up to 100 °C for 24 hours. Then the crude mixture was cooled down to room temperature and the solvent was removed under reduced pressure. After that, NaOH(aq) 30% (50 ml) was added to the crude and the aqueous phase was extracted using DCM (3 x 40 ml). The organic layer was washed with NaOH(aq) 30% (50 ml) and the solvent removed under reduced pressure. The brownish solid obtained was further purified by stirring overnight in hexane. The colorless solution was decanted and dried to obtain ^{Me}**L**$_{Br}$ (630 mg, 1.81 mmol, 75 % yield) as a white solid.

^{1}H-NMR ($CDCl_3$, 400 MHz, 298 K) δ (ppm): 7.15 (t, *J* = 8 Hz, 1H**g**), 6.78 (d, *J* = 8 Hz, 2H**f**), 6.69 (d, *J* = 8 Hz, 2H**b**), 6.61 (t, *J* = 8 Hz, 1H**a**), 4.26 (d, *J* = 12 Hz, 2H**e**), 3.94 (d, *J* = 12 Hz, 2H**e**), 3.65 (d, *J* = 12 Hz, 2H**c**), 3.53 (d, *J* = 12 Hz, 2H**c**), 2.70 (s, 6H**d**).
HR-ESI-MS: calcd. for $C_{17}H_{20}N_3Br$ $[M+H]^+$ 346.0913; exp 346.0914.

3. Synthesis of Iron(II) complexes: 1·Cl$_2$ and 1·Br$_2$

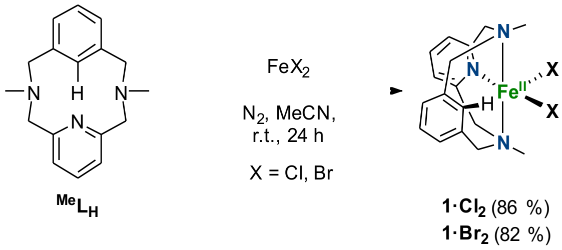

Scheme S8. Synthesis of **1·X$_2$** (X = Cl, Br).

In the glovebox, ^{Me}**L**$_H$ (75.3 mg, 0.28 mmol) and $FeCl_2$ (36.1 mg, 0.28 mmol) were dissolved in dry MeCN (2.0 ml). The mixture was left stirring 24 hours at room temperature. After that, the mixture was filtered over celite®. Slow Et_2O diffusion over a concentrated solution in MeCN/DCM at room temperature afforded yellow crystals corresponding to $[(^{Me}L_H)Fe^{II}(Cl)_2]$ (94.9 mg, 0.24 mmol, 86 % yield).

^{1}H-NMR (CD_3CN, 400 MHz, 298 K) δ (ppm): 139.33, 90.10, 65.63, 41.64, 32.26, 18.29, -5.72, -7.82, -39.45.
Evans' method: μ_{eff} = 4.81 MB (3.92 unpaired electrons).
HR-ESI-MS: calcd. for $C_{17}H_{21}N_3FeCl^+$, $[M-Cl^-]^+$ 358.0768; exp 358.0787.
EA: calcd. for $C_{17}H_{21}N_3FeCl_2$, C 51.10, N 10.66, H 5.37 %; exp. C 51.74, N 10.40, H 5.09 %.

FT-IR (ATR) ῡ (cm^{-1}) = 2877 (C_{sp3}-H str), 1604 (C_{sp2}-C_{sp2} str), 1446 (C_{sp3}-H bend), 1001 (C_{sp3}-N str), 797 (Fe-Cl str).

The analogous procedure using $FeBr_2$ was followed for the synthesis of **1·Br$_2$** (82% yield). HR-ESI-MS: calcd. for $C_{17}H_{21}N_3FeBr^+$, $[M\text{-}Br^-]^+$ 402.0264; exp 402.0267.

4. Reactivity of 1·Cl$_2$ towards PhMgBr reagent

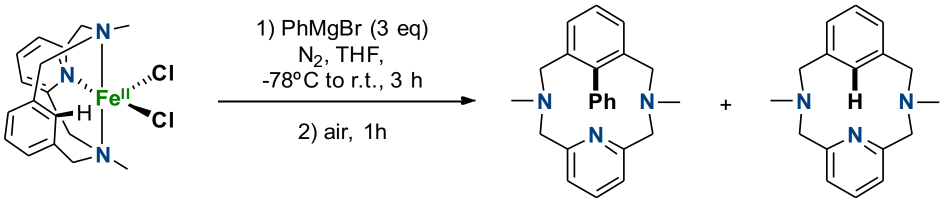

1·Cl$_2$ — 66 % $^{Me}L_{Ph}$ — 33 % $^{Me}L_{H}$

Scheme S9. Synthesis of ^{Me}**L**$_{Ph}$.

In the Schlenk line, complex **1·Cl$_2$** (31.10 mg, 0.1 mmol) was dissolved in anhydrous THF (1.5 ml). The yellow suspension was stirred below -78 °C and 3 equivalents of PhMgBr (236 µl of a 1.0 M solution in THF) were dropwise added via syringe. The mixture was left stirring below 78 °C for 30 min and then the temperature was increased to 0 °C for 1 hour. After that, the reaction was let to attain room temperature for 1 more hour and finally it was stirred under air for an additional hour. Next, concentrated HCl was added (together with the internal standard) and solvent was removed under reduced pressure. Ammonium hydroxide was used until pH > 14 was reached, and extractions were performed using DCM. The product was dried over a $MgSO_4$ plug and the solvent was removed. The final organic product was purified by silica gel chromatography using DCM:MeOH:NH_3 (95:5:1) as eluent. ^{Me}**L**$_{Ph}$ was obtained in a 66 % yield (^{1}H-NMR calculated using TMB as internal standard, 33% ^{Me}**L**$_{H}$ corresponding to the protodemetallation product).

^{1}H-NMR ($CDCl_3$, 400 MHz, 298 K) δ (ppm): 9.46 (d, *J* = 8 Hz, 1H**f**), 7.63 (td, *J* = 8, 1.5 Hz, 1H**g**), 7.41 (tt, *J* = 7.4, 1.4 Hz, 1H**h**), 7.32 (td, *J* = 7.4, 1.2 Hz, 1H**i**), 7.22 (t, *J* = 7.7 Hz, 1H**p**), 6.87 (d, *J* = 7.5 Hz, 2H**b**), 6.76 (t, *J* = 7.0 Hz, 3H**j,o**), 6.66 (t, *J* = 7.5 Hz, 1H**a**), 3.71 (d, *J* = 4 Hz, 4H**m**), 3.45 (s, 4H**k**), 2.53 (d, 6H**l**).
^{13}C-NMR ($CDCl_3$, 400 MHz, 298 K) δ (ppm): 158.90 (C**n**), 144.53 (C**d**), 140.47 (C**e**), 135.76 (C**c**), 135.08 (C**p**), 132.78 (C**f**), 131.61 (C**j**), 130.51 (C**b**), 127.23 (C**g**), 127.19 (C**i**), 126.67 (C**h**), 126.43 (C**a**), 120.63 (C**o**), 64.64 (C**m**), 61.83 (C**k**), 49.00 (C**l**).
HR-ESI-MS: calcd. for $C_{23}H_{25}N_3$ $[M+H]^+$ 344.2121; exp 344.2133.

5. Synthesis of Aryl-Iron(II) complexes

5.1. Synthesis of 1^{Me}

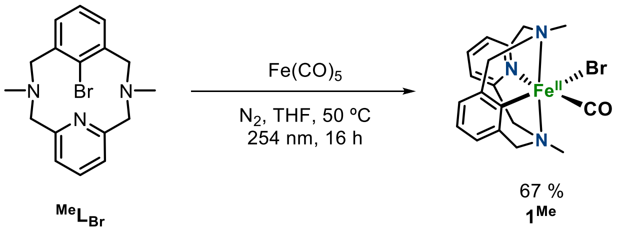

Scheme S10. Synthesis of $\mathbf{1^{Me}}$.

In the glovebox, $^{Me}\mathbf{L_{Br}}$ (53.2 mg, 0.15 mmol) and $Fe^0(CO)_5$ (20.21 µl, 0.15 mmol) were dissolved in dry THF (1.5 ml). The mixture was left stirring overnight at 50 ºC under UV light (254 nm) in a 8-lamp photoreactor. After that, rapidly, vacuum was applied to remove any CO present in the atmosphere and to remove the solvent from the reaction mixture. The mixture was filtered over celite® and slow Et_2O diffusion over a concentrated solution in THF at room temperature afforded green crystals corresponding to $\mathbf{1^{Me}}$ (44.3 mg, 0.10 mmol, 67 %).

^{1}H-NMR (THF-d_8, 400 MHz, 298 K) δ (ppm): 7.57 (t, *J* = 8 Hz, 1H**j**), 7.16 (d, *J* = 4 Hz, 2H**i**), 6.47 (s br, 3H**a,b**), 4.06 (d, *J* = 16 Hz, 2H**d**), 3.60 (m, 4H**g,d**), 3.50 (d, *J* = 16 Hz, 2H**g**), 2.42 (s, 6H**e**).
^{13}C-NMR (THF-d_8, 400 MHz, 298 K) δ (ppm): 223.59 (C**k**), 183.99 (C**f**), 160.09 (C**h**), 146.86 (C**c**), 136.67 (C**j**), 120.89 (C**a**), 118.76(C**i**), 118.35(C**b**), 76.46 (C**g**), 72.30 (C**d**), 54.89 (C**e**).
HR-ESI-MS: calcd. for $C_{19}H_{20}N_3O_2Fe^+$ 378.0900; exp 378.0905. Calcd for $C_{18}H_{20}N_3OFe^+$ 350.0950; exp 350.0888.
EA: calcd. for $C_{18}H_{20}N_3OFeBr{\cdot}0.25C_{17}H_{21}N_3$, C 53.77, N 10.57, H 5.12 %; exp. C 54.05, N 10.10, H 5.14 %.
FT-IR (ATR) ῡ (cm^{-1})= 2846 (C_{sp3}-H str), 1899 (Fe-CO str), 1447 (C_{sp3}-H bend), 1015 (C_{sp3}-N str), 761 (Fe-Br str).

5.2. Synthesis of 1^{H}

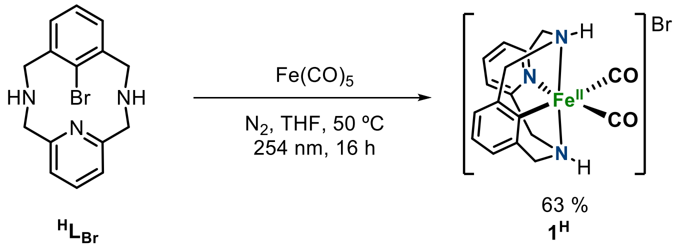

Scheme S11. Synthesis of $\mathbf{1^{H}}$.

In the glovebox, $^{H}L_{Br}$ (53.0 mg, 0.17 mmol) and $Fe^0(CO)_5$ (22.00 µl, 0.17 mmol) were dissolved in dry THF (1.5 ml). The mixture was left stirring overnight at 50 ºC under UV light (254 nm). After that, a green solid was formed and vacuum was applied to remove any CO present in the atmosphere. The solvent was decanted-off and the solid residue was redissolved in MeCN. Slow pentane diffusion over a

concentrated solution in MeCN afforded pale green crystals corresponding to **1**^{H} (46.1 mg, 0.11 mmol, 63 %).

^{1}H-NMR (DMSO-d$_6$, 400 MHz, 298 K) δ (ppm): 7.76 (t, *J* = 8 Hz, 1H**i**), 7.33 (d, *J* = 8 Hz, 2H**h**), 6.94-6.87 (m, 3H**a,b**), 5.63 (br, 2**NH**), 4.32 (d, *J* = 16 Hz, 2H**f**), 4.16 (m, 4H**f,d**), 3.93 (d, *J* = 16 Hz, 2H**d**).
^{13}C-NMR (DMSO-d$_6$, 400 MHz, 298 K): 184.73 (C**e**), 158.41 (C**g**), 145.70 (C**c**), 138.59 (C**i**), 124.43 (C**a**), 121.09 (C**b**), 120.23 (C**h**), 67.34 (C**d**), 64.47 (C**f**).
HR-ESI-MS: calcd. for $C_{16}H_{16}N_3OFe^+$ 322.0637; exp 322.0643.
EA: calcd. for $C_{17}H_{16}N_3O_2FeBr{\cdot}1.5H_2O$, C 44.67, N 9.19, H 4.19 %; exp. C 44.52, N 9.32, H 3.29 %.
FT-IR (ATR) ῡ (cm^{-1})= 3072 (N-H str), 2883 (C_{sp3}-H str), 2019 (Fe-CO str), 1963 (Fe-CO str), 1446 (C_{sp3}-H bend), 1234 (C_{sp3}-N str).

5.3. Synthesis of 1^{tBu}

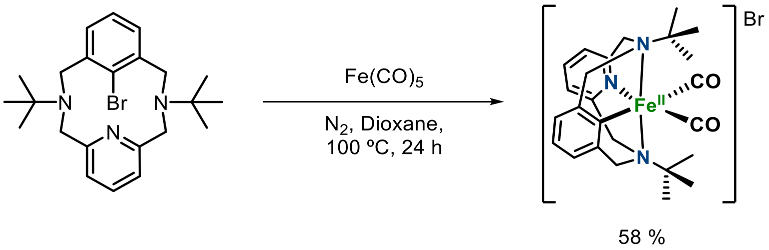

tBuLBr 1tBu

Scheme S12. Synthesis of **1**^{tBu}.

In the glovebox, ^{tBu}L$_{Br}$ (31.3 mg, 0.07 mmol) and Fe^{0}(CO)$_5$ (9.6 µl, 0.07 mmol) were dissolved in dry dioxane (1.0 ml). The mixture was left stirring for 24 hours at 100 ºC. After that, rapidly, vacuum was applied to remove any CO present in the atmosphere and to remove the solvent from the reaction mixture. **1**^{tBu} (20.9 mg, 0.04 mmol, 58 %) was obtained as a greenish foam.

^{1}H-NMR (DMSO-d$_6$, 400 MHz, 298 K) δ (ppm): 8.02 (t, *J* = 8 Hz, 1H**g**), 7.52 (d, *J* = 8 Hz, 2H**f**), 7.08 (s, 3H**a,b**), 4.44 (d, *J* = 16 Hz, 2H**c**), 4.31 (d, *J* = 16 Hz, 2H**c**), 3.91 (d, *J* = 16 Hz, 2H**e**), 3.63 (d, *J* = 16 Hz, 2H**e**), 1.23 (br, 18H**d**).
HR-ESI-MS: calcd. for $C_{24}H_{32}N_3OFe^+$ 434.1890; exp 434.1905.

6. Reactivity of 1^{Me} with phenyl Grignard (PhMgBr)

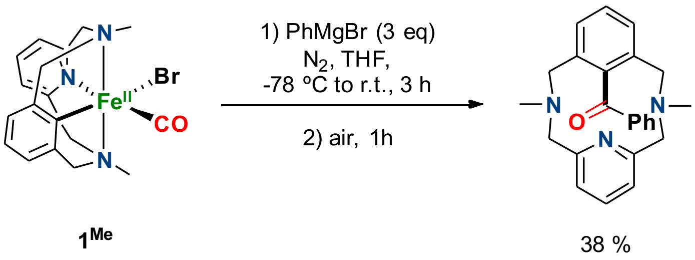

1Me MeLPh

Scheme S13. Synthesis of ^{Me}L$_{COPh}$.

In the Schlenk line, complex **1**^{Me} (26.0 mg, 0.06 mmol) was dissolved in anhydrous THF (1.5 ml). The yellow suspension was stirred below -78 ºC and 3 equivalents of PhMgBr (181 µl of a 1.0 M solution

inTHF) were dropwise added via syringe. The mixture was left stirring below 78 ºC for 30 min and then the temperature was increased to 0 ºC for 1 hour. After that, the reaction was let to attain room temperature for 1 more hour and finally it was stirred under air for an additional hour. Next, concentrated HCl was added (together with the internal standard) and solvent was removed under reduced pressure. Ammonium hydroxide was used until pH > 14 was reached, and extractions were performed using DCM. The product was dried over a $MgSO_4$ plug and the solvent was removed. The final organic product was purified by silica gel chromatography using DCM:MeOH:NH_3 (95:5:1) as eluent. $^{Me}\mathbf{L}_{COPh}$ was obtained in a 38 % yield (^{1}H-NMR calculated using TMB as internal standard, 7% $^{Me}\mathbf{L}_{Ph}$ and 5% $^{Me}\mathbf{L}_{H}$ corresponding to the protodemetallation product).

^{1}H-NMR ($CDCl_3$, 400 MHz, 298 K) δ (ppm): 7.58 (d, *J* = 8 Hz, 2H**g**), 7.47 (t, *J* = 8, Hz, 1H**i**), 7.35 (t, *J* = 8 Hz, 2H**h**), 7.14 (t, *J* = 8 Hz, 1H**o**), 6.85 (br, 3H**a,b**), 6.67 (d, *J* = 8 Hz, 2H**n**), 4.26 (br, 2H**l**), 3.68 (br, 2H**j**), 3.53 (br, 2H**l**), 3.31 (br, 2H**j**), 2.46 (s, 6H**k**).
^{13}C-NMR ($CDCl_3$, 400 MHz, 298 K) δ (ppm): 195.00 (C**e**), 158.12 (C**m**), 140.49 (C**f**), 139.00 (C**d**), 137.61 (C**c**), 135.32 (C**o**), 132.04 (C**i**), 130.46 (C**b**), 129.05 (C**g**), 128.75 (C**a**), 128.22 (C**h**), 121.58 (C**n**), 64.86 (C**l**), 61.38 (C**j**), 48.91 (C**k**).

HR-ESI-MS: calcd. for $C_{24}H_{25}N_3O$, $[M+H]^+$ 372.2071; exp 372.2070.

FT-IR (ATR) ῡ (cm^{-1})= 2922 (C_{sp3}-H str), 1672 (C_{sp2}-O str), 1447 (C_{sp3}-H bend), 1122 (C_{sp3}-N str).

7. Mechanistic insights

7.1. UV-vis monitoring of 1·Cl₂ with PhMgBr

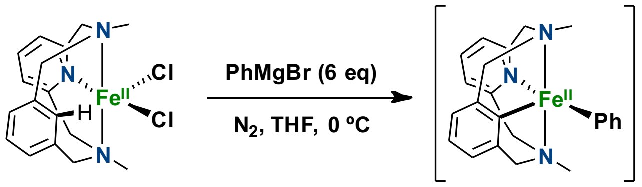

1·Cl$_2$ **C**

Scheme S14. Reaction of **1·Cl$_2$** towards PhMgBr monitored by UV-vis spectroscopy at 0 ºC.

A. A UV-vis cell was charged with 2.2 ml of a 0.5 mM solution of **1·Cl$_2$** in anhydrous THF prepared in the glovebox. The quartz cell was capped with a septum, taken out of the glovebox, and placed in a Unisoku thermostated cell holder designed for low-temperature experiments at 273 K. Once the thermal equilibrium was reached, a UV-vis spectrum of the starting complex was recorded. PhMgBr (10 equiv) was injected into the cell through the septum causing immediate formation of two bands at λ_{max} = 520 nm and λ_{max} = 635 nm corresponding to the generation of the green (C) species proposed (Figure S1). Maximum formation of such species is formed after ca. 13 min.

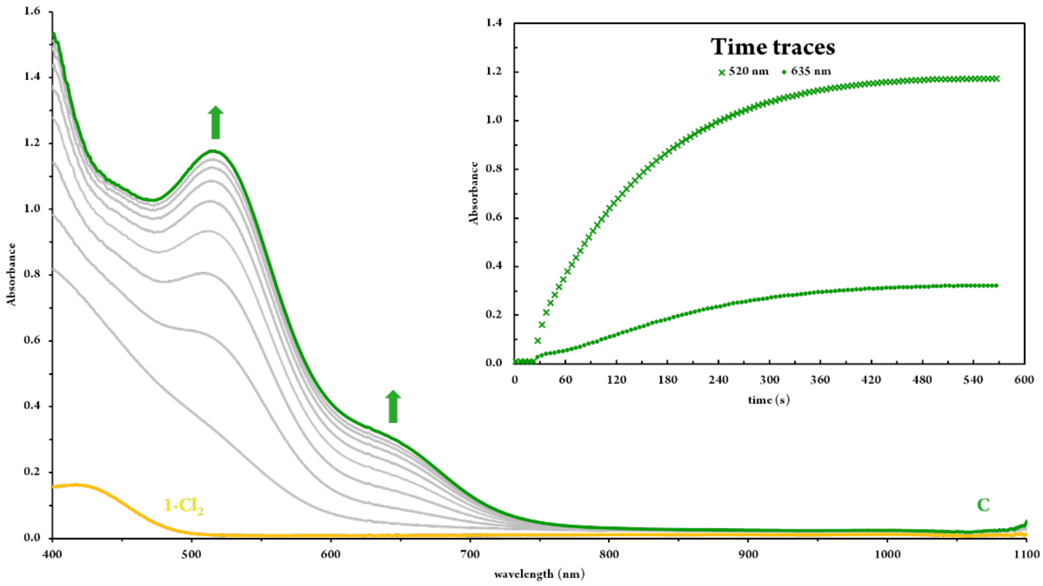

Figure S1. UV-vis spectra of the reaction of **1·Cl$_2$** (yellow) towards PhMgBr at 0 °C to form species **C** (green) with the corresponding bands at 520 and 635 nm.

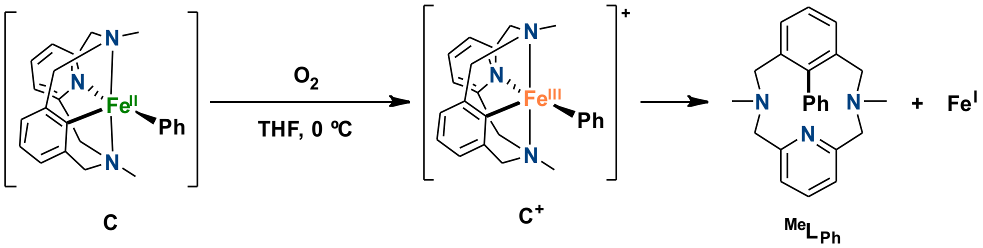

Scheme S15. Reaction of **C** towards dioxygen monitored by UV-vis spectroscopy at room temperature.

B. A first UV-vis spectrum of the new formed species **C** was recorded. Dioxygen was bubbled into the cell through the septum causing immediate decay of the two bands at λ_{max} = 520 nm and λ_{max} = 635 assigned to green species (C). Subsequent formation of new species was rapidly observed (< 20 seconds, orange trace in Figure S2a) corresponding to the putative C^+ species that rapidly evolves (< 10 seconds) to the final reaction mixture (purple trace in Figure S2a).

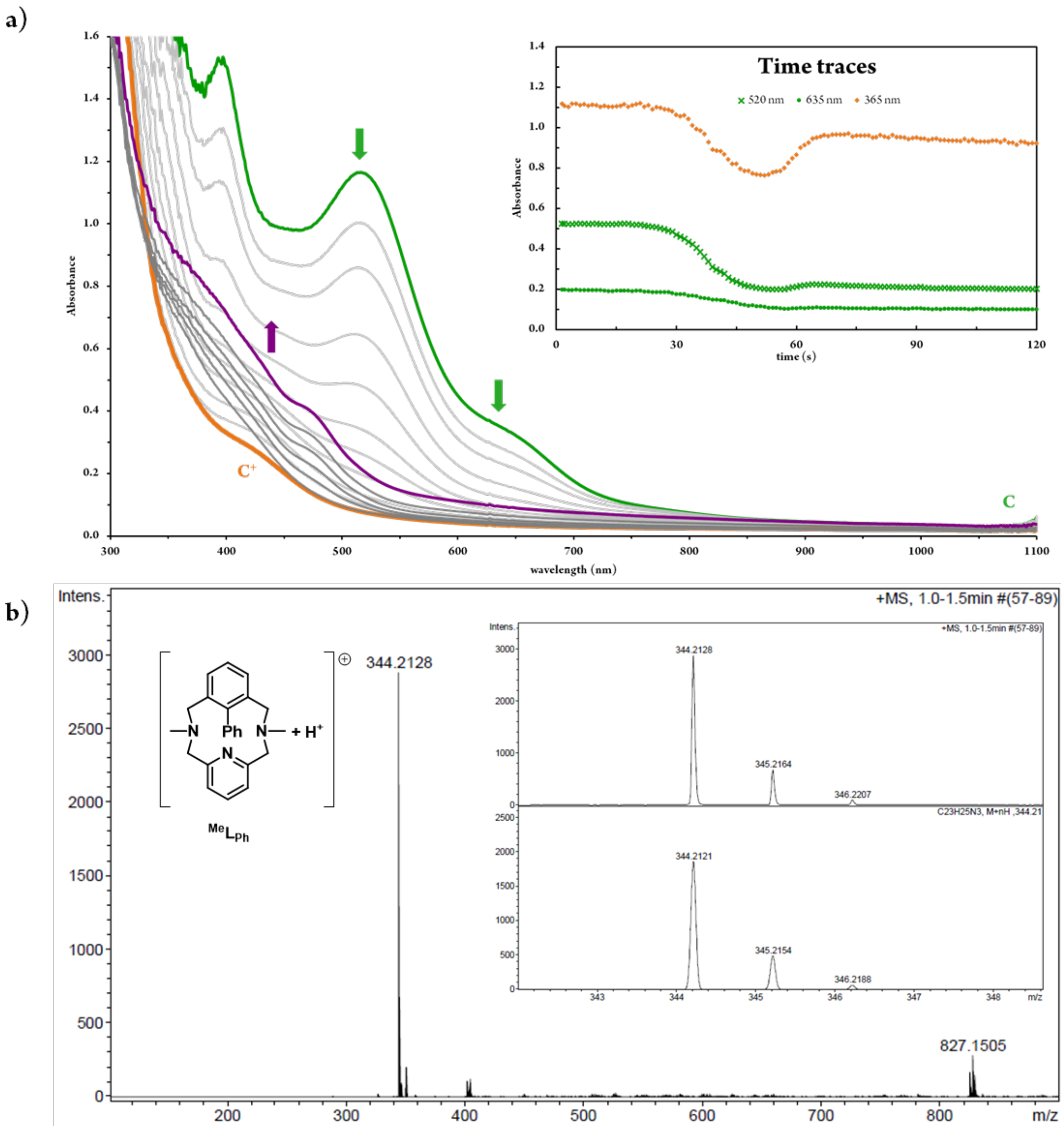

Figure S2. a) UV-vis spectra of the reaction of species **C** (green) towards dioxygen at 0 ºC. Decay of the bands at 520 and 635 nm to form unstable species **C**$^+$ (orange) and immediate formation of the new band at 365 nm (purple) corresponding to the final mixture. b) Cryo-HR-MS spectra of ^{Me}**L**$_{Ph}$. Inset: expanded view of the experimental peak at a m/z = 344.2128 corresponding to the monocharged [$C_{23}H_{25}N_3$+H]$^+$ (top) and the expanded view of the corresponding calculated spectrum for this molecular formula (bottom).

<u>Attempts to characterize **C**$^+$ by Cryospray ionization/HR-MS</u>

In order to perform HR-MS analysis, in a typical UV-vis experiment, the generation of species **C** was monitored at 0 ºC. Once it was fully formed the sample was cooled down to -45 ºC. On a separated vial containing DCM, previously cooled down and purged with dioxygen, an aliquot from the UV-vis cuvette was added and then it was immediately injected into the mass spectrometer equipped with cryospray ionization at -45 ºC. However, all attempts to gain information about the nature of species **C**$^+$ were unfruitful due to its extremely high reactivity. Nevertheless, a clean mass spectrum of the final coupling biaryl product ^{Me}**L**$_{Ph}$ was obtained (m/z = 344.2128 corresponding to [$C_{23}H_{25}N_3$+H]$^+$) as shown in Figure S2b.

7.2. Inert atmosphere work-up for $1{\cdot}Cl_2$ and 1^{Me} towards phenyl Grignard

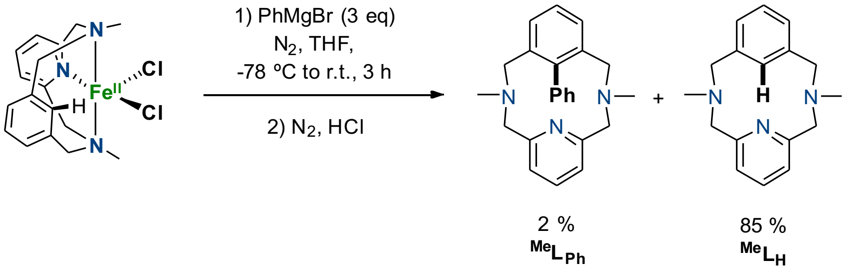

Scheme S16. Reaction of **1·Cl₂** towards PhMgBr in absence of oxidant.

In the Schlenk line, complex $\mathbf{1{\cdot}Cl_2}$ (12.00 mg, 0.03 mmol) was dissolved in anhydrous THF (1.0 ml). The yellow suspension was stirred at -78 ºC and 3 equivalents of PhMgBr (120 µl of a 1.0 M solution in THF) were dropwise added via syringe. The mixture was left stirring at -78 ºC for 30 min and then the temperature was increased to 0 ºC for 1 hour. After that, the reaction was let to attain room temperature for 1 more hour. Next, concentrated HCl was added (together with the internal standard) under N_2 atmosphere and solvent was removed under vacuum. Ammonium hydroxide was used until pH >14 was reached, and extractions were performed using DCM. The product was dried over a $MgSO_4$ plug, filtered and the solvent was removed. $^{Me}\mathbf{L}_{Ph}$ was obtained in a 2% yield (^{1}H-NMR calculated using TMB as internal standard, 85% $^{Me}L_H$ corresponding to the protodemetallation product).

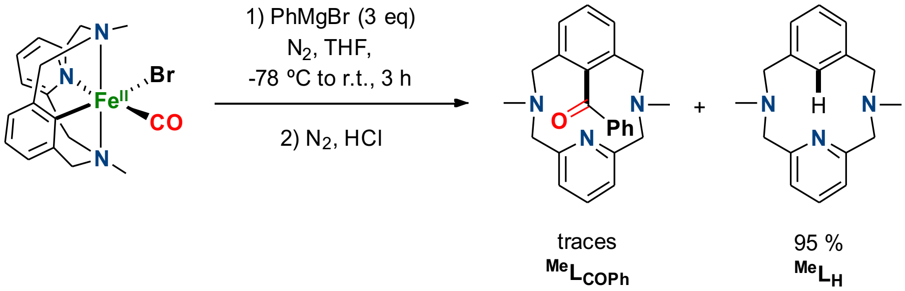

Scheme S17. Reaction of $\mathbf{1^{Me}}$ towards PhMgBr in absence of oxidant.

In the Schlenk line, complex $\mathbf{1^{Me}}$ (26.00 mg, 0.06 mmol) was dissolved in anhydrous THF (1.5 ml). The yellow suspension was stirred at -78 ºC and 3 equivalents of PhMgBr (181 µl of a 1.0 M solution in THF) were added dropwise. The mixture was left stirring at -78 ºC for 30 min and then the temperature was increased to 0 ºC for 1 hour. After that, the reaction was let to attain room temperature for 1 more hour. Next, concentrated HCl was added (together with the internal standard) under N_2 atmosphere and solvent was removed under vacuum. Ammonium hydroxide was used until pH >14 was reached, and extractions were performed using DCM. The product was dried over a $MgSO_4$ plug, filtered and the solvent was removed. $^{Me}\mathbf{L}_{COPh}$ was obtained in a trace amounts (^{1}H-NMR calculated using TMB as internal standard, 95% $^{Me}\mathbf{L}_H$ corresponding to the protodemetallation product).

7.3. Use of DCIB as oxidant for the reaction of 1·Cl_2 with PhMgBr

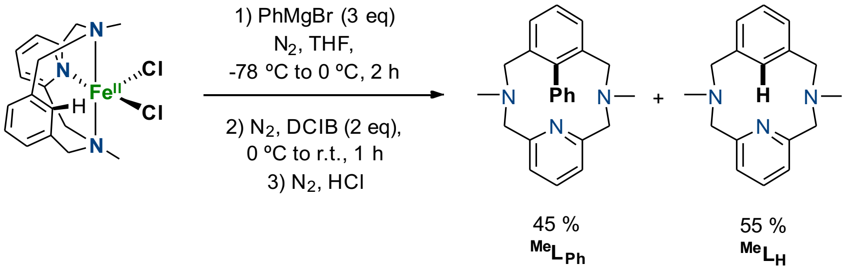

Scheme S18. Reaction of **1·Cl_2** towards PhMgBr in presence of DCIB as oxidant.

In a typical experiment, in the Schlenk line, complex **1·Cl_2** (32.90 mg, 0.08 mmol) was dissolved in anhydrous THF (1.6 ml). The yellow suspension was stirred below -78 ºC and 3 equivalents of PhMgBr (251 µl of a 1.0 M solution in THF) were added dropwise via syringe. The mixture was left stirring below 78 ºC for 30 min and then the temperature was increased to 0 ºC for 1.5 hour. After that, 2 equivalents of DCIB, 1,2-dichloroisobutane (20.4 µl), were added the reaction was let to attain room temperature for 1 more hour. Next, concentrated HCl was added (together with the internal standard) under N_2 atmosphere and solvent was removed under vacuum. Ammonium hydroxide was used until pH >14 was reached, and extractions were performed using DCM. The product was dried over a $MgSO_4$ plug, filtered and the solvent was removed. **$^{Me}L_{Ph}$** was obtained in a 45% yield (^{1}H-NMR calculated using TMB as internal standard, 55% **$^{Me}L_H$** corresponding to the protodemetallation product).

Noteworthy, addition of DCIB with the rest of reagents at the beginning of the reaction only afforded 9% yield of **$^{Me}L_{Ph}$**. Attempts to conduct a catalysis with DCIB under N_2 using 20 mol% of $FeCl_2$ were unfruitful, and only 4% yield of **$^{Me}L_{Ph}$** was obtained.

7.4. Use of CO atmosphere for the reaction of 1·Cl_2 with PhMgBr

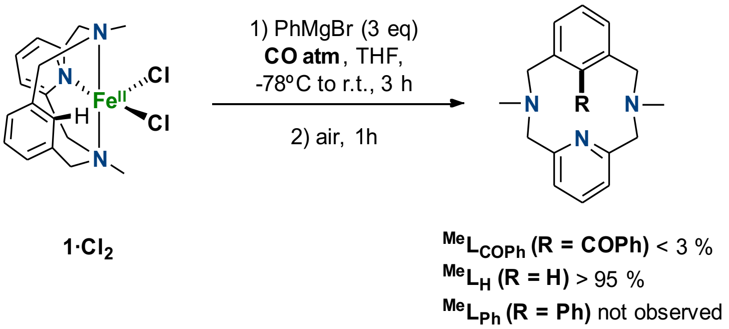

Scheme S19. Reaction of **1·Cl_2** towards PhMgBr in presence of CO atmosphere.

In a typical experiment, in the Schlenk line, complex **1·Cl_2** (38.90 mg, 0.10 mmol) was dissolved in anhydrous THF (1.5 ml). Once dissolved, a CO atm was provided. The yellow suspension was stirred below -78 ºC and 3 equivalents of PhMgBr (300 µl of a 1.0 M solution in THF) were added dropwise via syringe. The mixture was left stirring below 78 ºC for 30 min and then the temperature was increased to 0 ºC for 1 hour. After that, the reaction was let to attain room temperature for 1 more hour and finally it was stirred under air for an additional hour. Next, concentrated HCl was added (together with the internal standard) and solvent was removed under reduced pressure. Ammonium hydroxide was used until pH >14 was reached, and extractions were performed using DCM. The product was dried over a

$MgSO_4$ plug and the solvent was removed. ^{Me}**L**$_{COPh}$ was obtained in less than a 3 % yield (^{1}H-NMR calculated using TMB as internal standard, >95 % ^{Me}**L**$_H$ corresponding to the protodemetallation product and ^{Me}**L**$_{Ph}$ was not observed).

7.5. Reaction of 1^{Me} with PhMgBr under photoirradiation (254 nm).

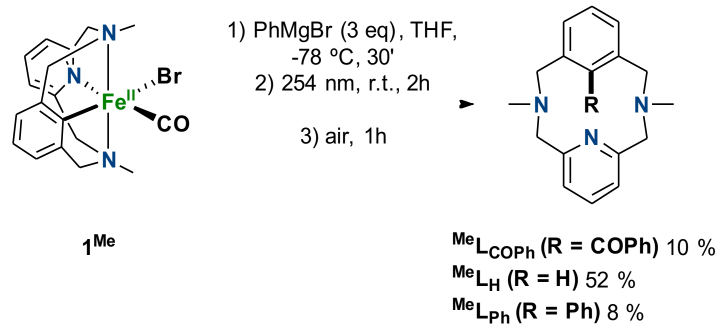

Scheme S20. Reaction of **1**^{Me} towards PhMgBr in presence of CO atmosphere.

In the Schlenk line, complex **1**^{Me} (31.9 mg, 0.07 mmol) was dissolved in anhydrous THF (1.5 ml). The solution was stirred at -78 °C and 3 equivalents of PhMgBr (230 µl of a 1.0 M solution in THF) were added dropwise. The mixture was left stirring at -78 °C for 30 min. After that, the mixture was irradiated with 254 nm lamps and then the temperature was increased to room temperature for 2 hours and opened to air for 1 more hour. Next, concentrated HCl was added (together with the internal standard) and solvent was removed under vacuum. Ammonium hydroxide was used until pH >14 was reached, and extractions were performed using DCM. The product was dried over a $MgSO_4$ plug, filtered and the solvent was removed. ^{Me}**L**$_{Ph}$ was obtained in an 8 % yield (^{1}H-NMR calculated using TMB as internal standard, 52 % ^{Me}**L**$_H$ corresponding to the protodemetallation product and a 10 % corresponding to ^{Me}**L**$_{COPh}$).

7.6. Attempts to isolate species C' ([Fe^{II}(MeL)(Ph)(PPh$_3$)])

Attempt 1

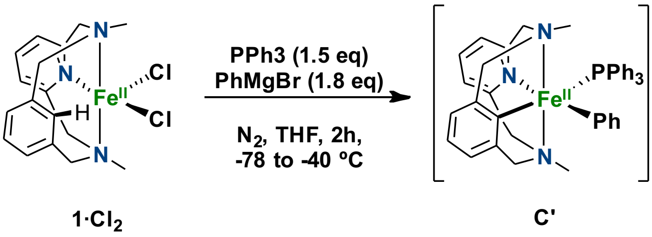

Scheme S21. Attempt to synthetize intermediate **C'** with PPh_3 at low temperature.

Under a nitrogen atmosphere, **1·Cl**$_2$ (37.4 mg, 0.09mmol) and THF (2 mL) were added to a 25 mL round bottom flask stirring at -78 °C. Once thermal equilibrium was reached, PhMgBr (170 µL, 1.8 eq) was added dropwise and stirred for 30 min. Then temperature was increased to -40 °C generating a red-colored solution. At this point 1.5 eq of PPh_3 (37.7 mg in 0.5 mL THF) were added. After that, the solution was cannulated at -40 °C to remove the precipitate salts. Crystallization by slow diffusion over hexane at -40 °C did not afford crystalline material but decomposition of the complex. HR-ESI-MS of the crude shows the 344.21 peak corresponding to the ^{Me}**L**$_{Ph}$ biaryl formation via C-C reductive elimination.

Attempt 2

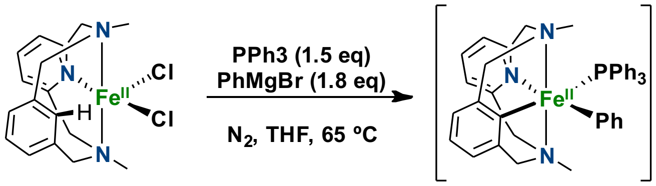

1·Cl2 **C'**

Scheme S22. Attempt to synthetize intermediate **C'** with PPh_3 at 65 °C.

Under a nitrogen atmosphere, **1·Cl2** (35.4 mg, 0.09mmol), PPh_3 (35.4 mg, 1.5 eq) and THF (3 mL) were added to a 25 mL round bottom flask stirring at RT for 1 hour. Temperature was lowered to -78°C. Once thermal equilibrium was reached, PhMgBr (150 µL, 1.8 eq) was added to the vial containing the iron/PPh_3 solution dropwise generating a red-colored solution after heating at 65 °C for 2 hours. After that, temperature was lowered to -40°C and the solution was cannulated to remove the precipitate salts. Crystallization by slow diffusion over hexane at -40 °C. Compound **C'** was neither detected, although PPh_4^+ was detected by HR-ESI-MS, suggesting a P-C reductive elimination from **C'**.

8. Amine-to-amide CO insertion reactivity

8.1. Synthesis of $2^{Me}(CO)_H$ and $^{Me}L\text{-}CO_H$

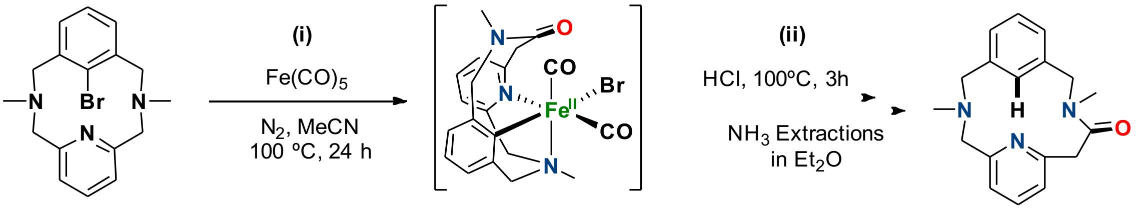

$^{Me}L_{Br}$ 80 % $2^{Me}(CO)$ 99 % $^{Me}L\text{-}CO_H$

Scheme S23. **(i)** Synthesis of 2^{Me}**(CO)**. **(ii)** Synthesis of ^{Me}**L-CO**$_H$

(i) In the glovebox, $^{Me}L_{Br}$ (31.8 mg, 0.09 mmol) and $Fe^0(CO)_5$ (12.13 µl, 0.09 mmol) were dissolved in dry MeCN (1 ml). The mixture was left stirring for 24 hours at 100 °C. After that, rapidly, vacuum was applied to remove any CO present in the atmosphere and to remove the solvent from the reaction mixture. 2^{Me}**(CO)** was obtained as a red-brown foam (33.7 mg, 0.07 mmol, 80 %).

^{1}H-NMR (DMSO-d$_6$, 400 MHz, 298 K) δ (ppm): 7.50 (t, *J* = 8 Hz, 1H**k**), 7.47 (d, *J* = 8 Hz, 1H**c**), 7.40 (t, *J* = 8 Hz, 1H**b**), 7.29 (d, *J* = 8 Hz, 1H**a**), 7.17 (d, *J* = 8 Hz, 1H**j**), 6.95 (d, *J* = 8 Hz, 1H**l**), 4.27 (s, 2H**q**), 4.00 (s, 2H**f**), 3.50 (s, 2H**h**), 2.90 (s, 3H**p**), 2.28 (s, 2H**n**), 2.01 (s, 3H**g**).
^{13}C-NMR (DMSO-d$_6$, 400 MHz, 298 K) δ (ppm): 210.78 (C**s**), 168.23 (C**o**), 159.04 (C**i**), 157.22 (C**m**), 142.41 (C**r**), 137.82 (C**d**), 137.19 (C**k**), 131.11 (C**b**), 129.88 (C**e**), 128.37 (C**c**), 121.96 (C**a**), 121.64 (C**l**), 119.65 (C**j**), 63.42 (C**h**), 54.77 (C**f**), 51.26 (C**q**), 43.96 (C**g**), 29.37 (C**p**), 24.44 (C**n**).

FT-IR (ATR) ῡ (cm^{-1})= 2837 (C_{sp3}-H str), 1890 (Fe-CO str), 1677 (C_{sp2}-O str), 1451 (C_{sp3}-H bend), 1024 (C_{sp3}-N str).

(ii) To $\mathbf{2^{Me}(CO)}$ HCl was added and the reaction was left stirring at 100 ºC for 3 additional hours. After that, NH_4OH was added until pH 14. Then extractions were performed in Et_2O. The organic layer was dried in $MgSO_4$, filtered and solvent was removed under vacuum to obtain $\mathbf{^{Me}L\text{-}CO_H}$ (99 %, NMR yield).

^{1}H-NMR ($CDCl_3$, 400 MHz, 298 K) δ (ppm): 7.68 (d, *J* = 8 Hz, 1H**a**), 7.53 (t, *J* = 8 Hz, 1H**l**), 7.47 (t, *J* = 8 Hz, 1H**b**), 7.39 (s, 1H**e**), 7.35 (d, *J* = 8 Hz, 1H**k**), 7.28 (d, *J* = 8 Hz, 1H**c**), 6.98 (d, *J* = 8 Hz, 1H**m**), 4.32 (s, 2H**f**), 4.27 (s, 2H**q**), 3.77 (s, 2H**i**), 3.16 (s, 3H**g**), 2.53 (s, 2H**o**), 2.29 (s, 3H**p**).
^{13}C-NMR ($CDCl_3$, 400 MHz, 298 K) δ (ppm): 169.21 (C**h**), 159.37 (C**j**), 157.66 (C**n**), 141.50 (C**d**), 138.88 (C**r**), 136.72 (C**l**), 130.94 (C**b**), 130.17 (C**c**), 128.88 (C**a**), 121.44 (C**m**), 121.06 (C**e**), 119.93 (C**k**), 63.85 (C**i**), 55.45 (C**q**), 51.60 (C**f**), 42.59 (C**p**), 29.45 (C**g**), 24.62 (C**o**).
HR-ESI-MS: calcd. for $C_{18}H_{21}N_3O$, $[M+H]^+$ 296.1757; exp 296.1775. (See Figure S57).

FT-IR (ATR) ῡ (cm^{-1})= 2942 (C_{sp3}-H str), 1679 (C_{sp2}-O str), 1453 (C_{sp3}-H bend), 1044 (C_{sp3}-N str).

8.2. Mechanistic insights

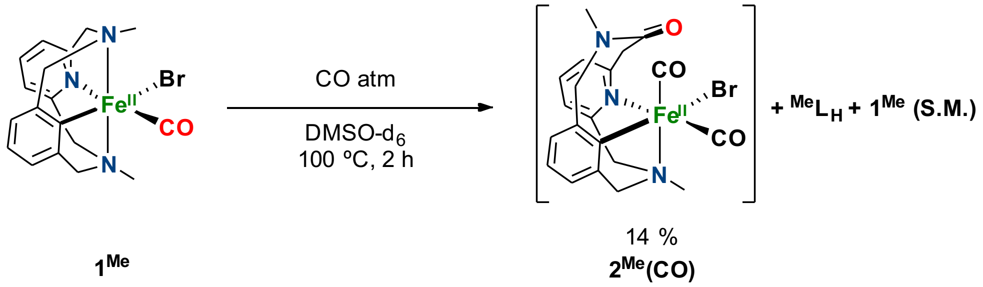

Scheme S24. Synthesis of $\mathbf{2^{Me}(CO)}$ starting from the well-defined $\mathbf{1^{Me}}$.

In the glovebox, $\mathbf{1^{Me}}$ (19.6 mg, 0.05 mmol) were dissolved in dry DMSO-d_6 (1 ml). The mixture was left stirring for 2 hours at 100 ºC under a CO atmosphere. After that, rapidly, vacuum was applied to remove any CO present in the atmosphere and the reaction crude was checked by ^{1}H-NMR. After only 2 hours of reaction a 14 % of $\mathbf{2^{Me}(CO)}$ could be observed together with a 14 % of $\mathbf{^{Me}L_H}$ (protodemetallation by-product) and 71 % of starting complex ($\mathbf{1^{Me}}$).

8.3. Amine-to-amide CO insertion reactivity using $^{H}L_{Br}$

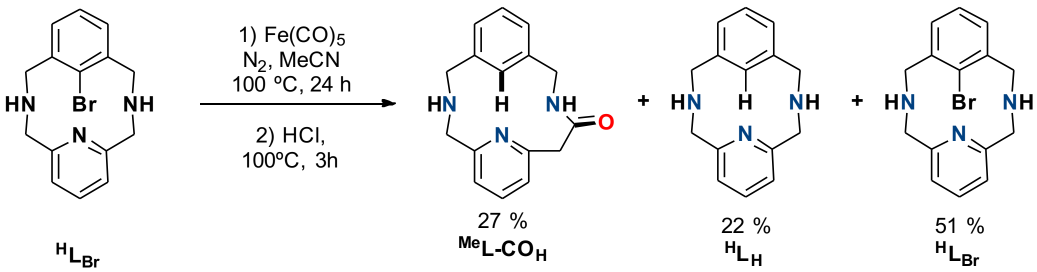

Scheme S25. Synthesis of ^{H}L-CO$_H$

In the glovebox, $^{H}L_{Br}$ (32.6 mg, 0.10 mmol) and $Fe^{0}(CO)_5$ (13.48 µl, 0.10 mmol) were dissolved in dry MeCN (1 ml). The mixture was left stirring for 24 hours at 100 ºC. After that, HCl was added and the reaction was left stirring at 100 ºC for 3 additional hours. Then, NH_4OH was added until pH 14 and extractions were performed in Et_2O. The organic layer was dried in $MgSO_4$, filtered and solvent was removed under vacuum to obtain $^{H}L\text{-}CO_H$ (27 %, ^{1}H NMR yield). HR-ESI-MS: calcd. for $C_{16}H_{17}N_3O$ $[M+H]^+$: 268.1450; exp: 268.1446 (See Figure S3).

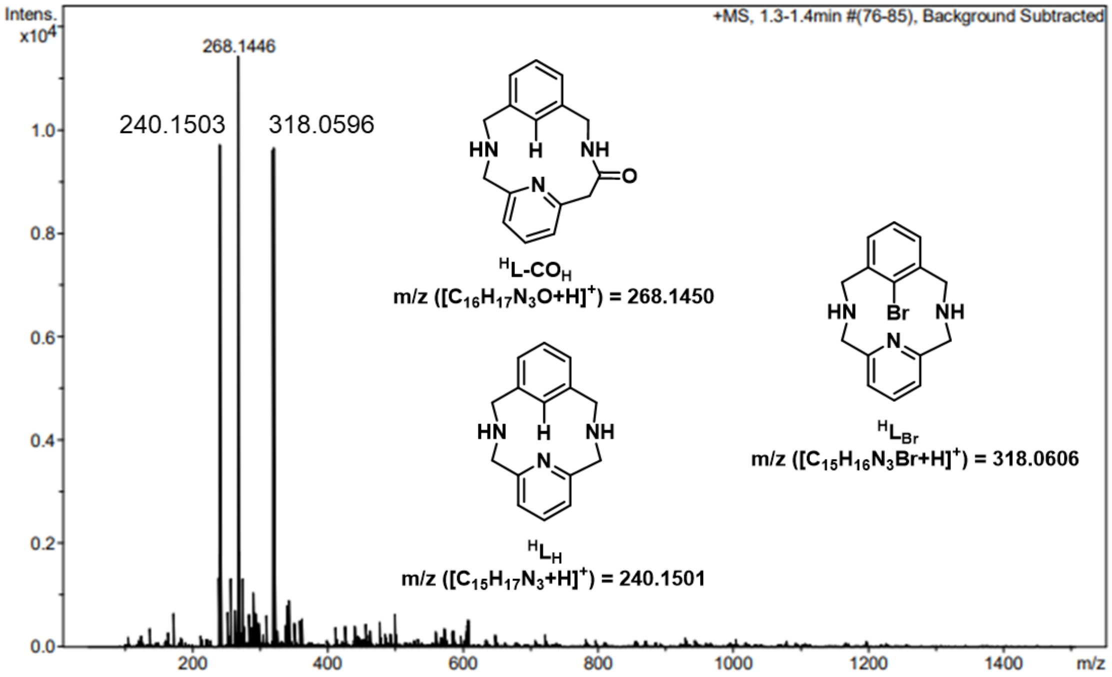

Figure S3. HR-ESI-MS of the reaction crude for the synthesis of ^{H}**L-CO**$_{H}$. Three main peaks could be observed. The experimental peak at m/z = 268.1446 with a mass value and an isotopic pattern fully consistent with the monocharged $[C_{16}H_{18}N_3O]^+$. The experimental peak at m/z = 240.1503 with a mass value and an isotopic pattern fully consistent with the monocharged $[C_{15}H_{18}N_3]^+$. The experimental peak at m/z = 318.0596 with a mass value and an isotopic pattern fully consistent with the monocharged $[C_{15}H_{17}N_3Br]^+$.

9. Spectroscopic characterization

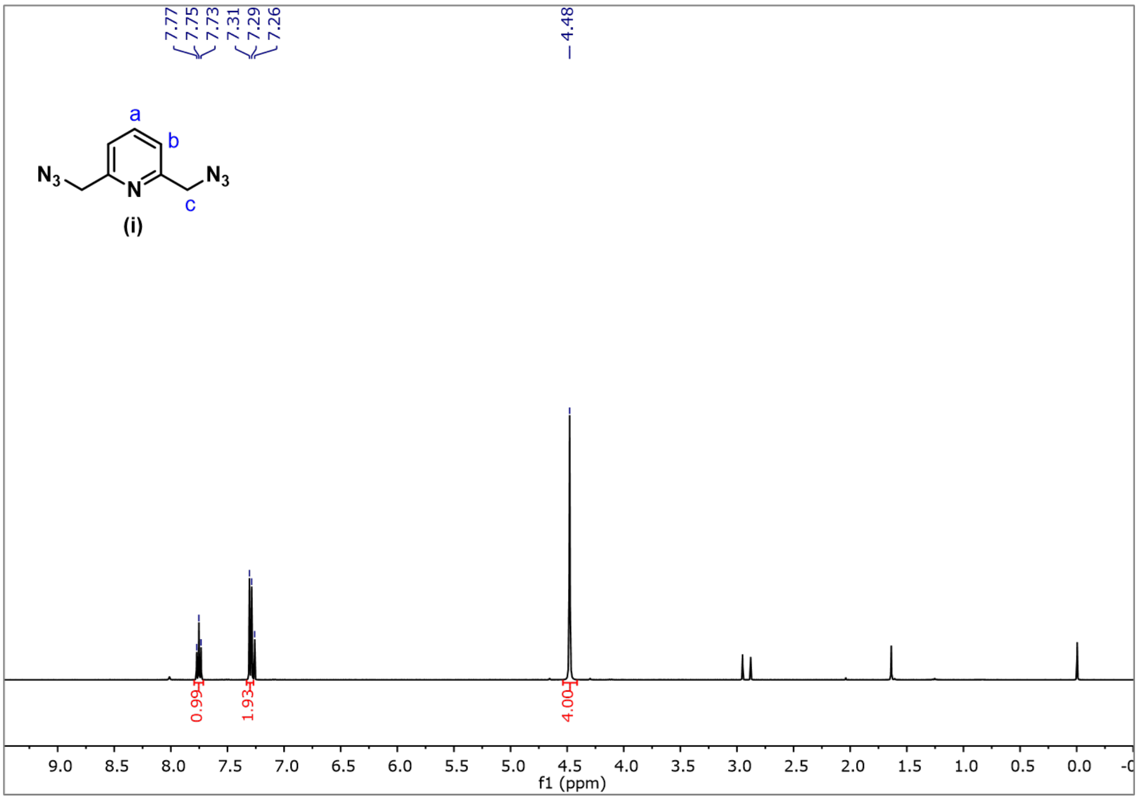

Figure S4. ^{1}H-NMR spectrum of **(i)** in $CDCl_3$ at room temperature (400 MHz).

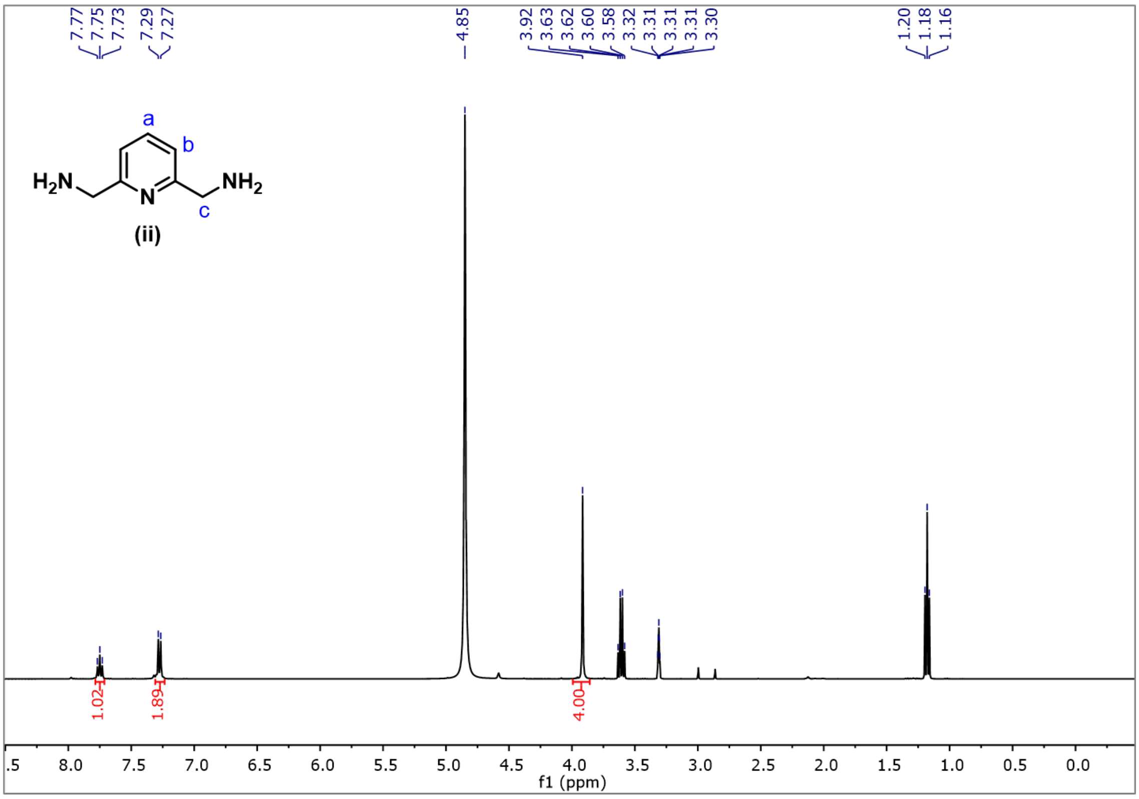

Figure S5. ^{1}H-NMR spectrum of **(ii)** in CD_3OD at room temperature (400 MHz).

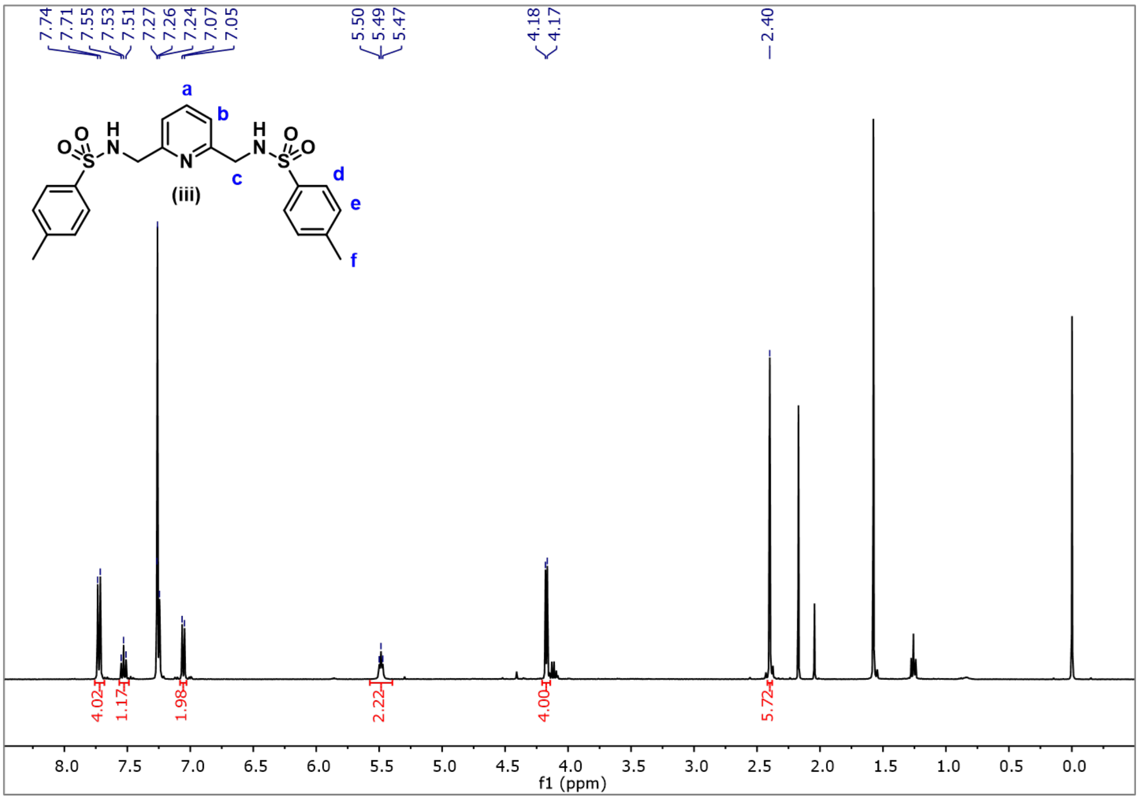

Figure S6. ^{1}H-NMR spectrum of **(iii)** in $CDCl_3$ at room temperature (400 MHz).

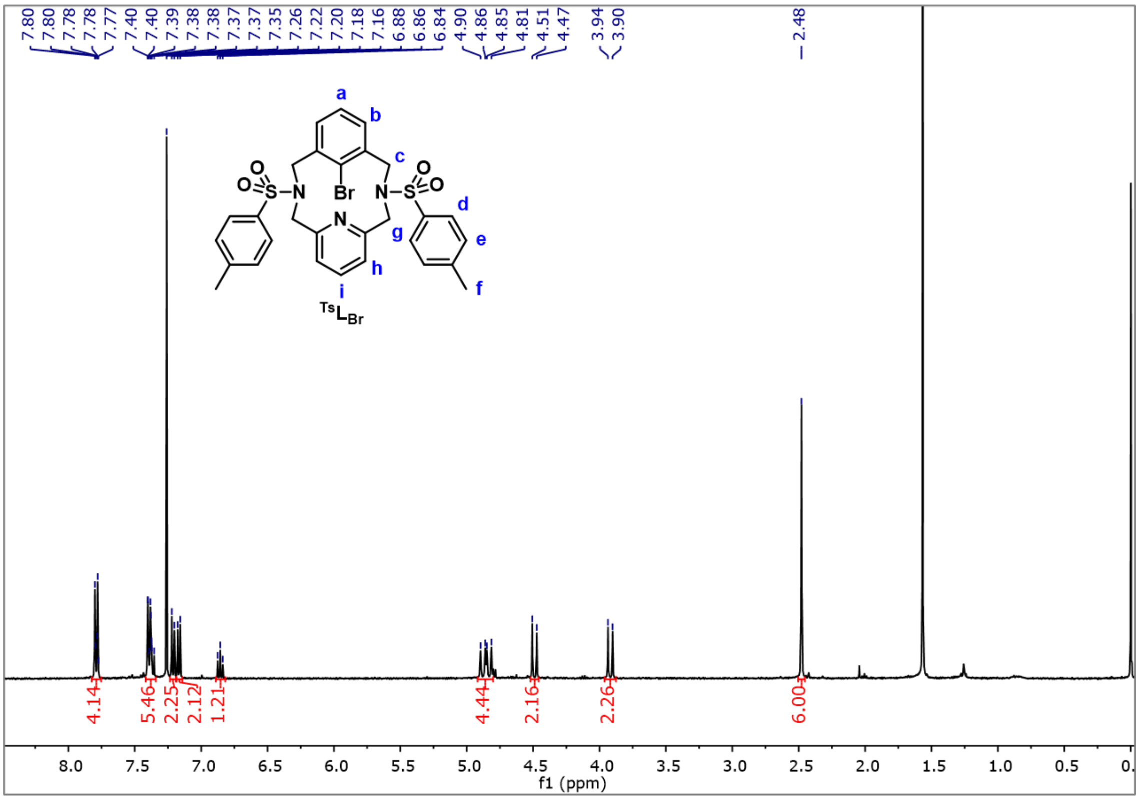

Figure S7. ^{1}H-NMR spectrum of ^{Ts}**L**$_{Br}$ in $CDCl_3$ at room temperature (400 MHz).

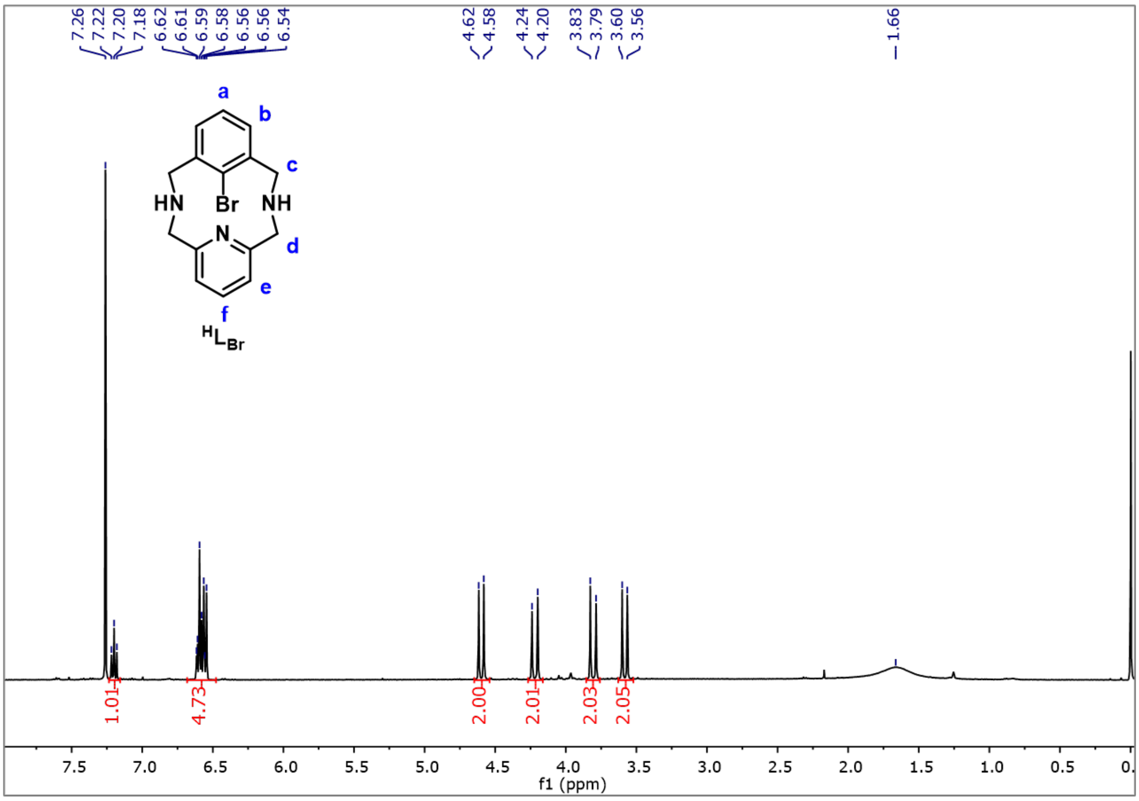

Figure S8. ^{1}H-NMR spectrum of $^{H}\mathbf{L}_{Br}$ in $CDCl_3$ at room temperature (400 MHz).

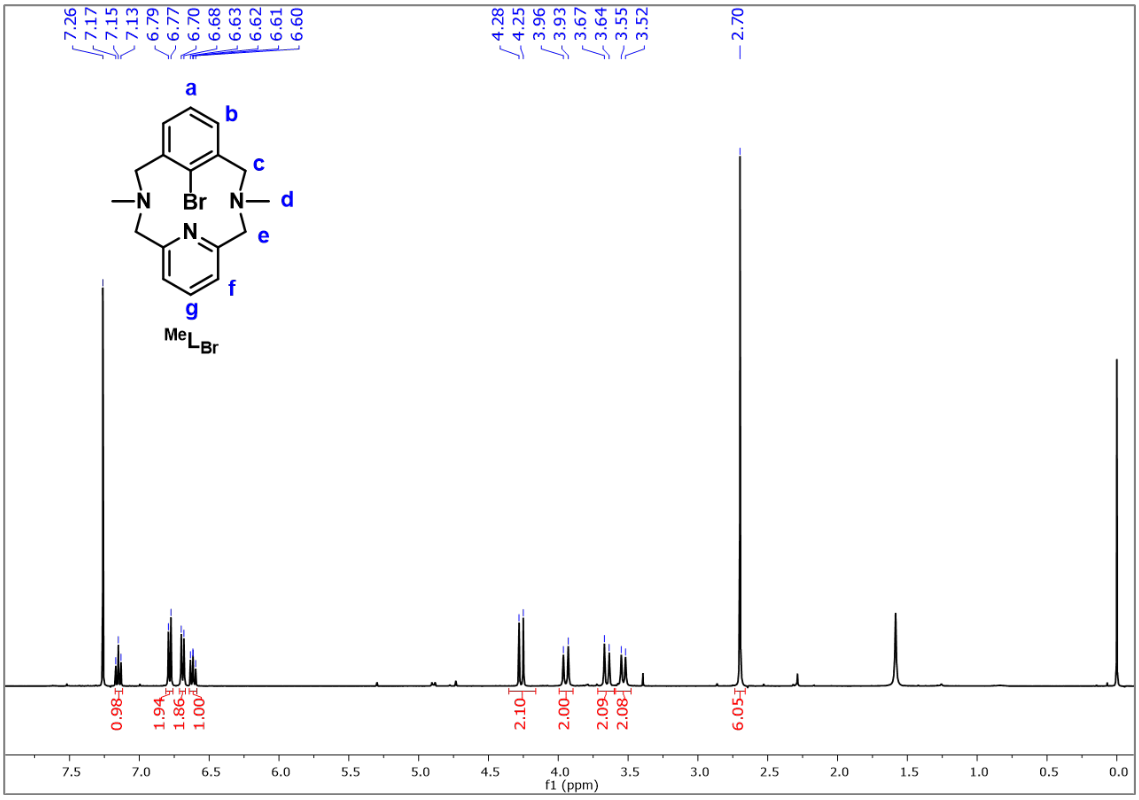

Figure S9. ^{1}H-NMR spectrum of $^{Me}\mathbf{L}_{Br}$ in $CDCl_3$ at room temperature (400 MHz).

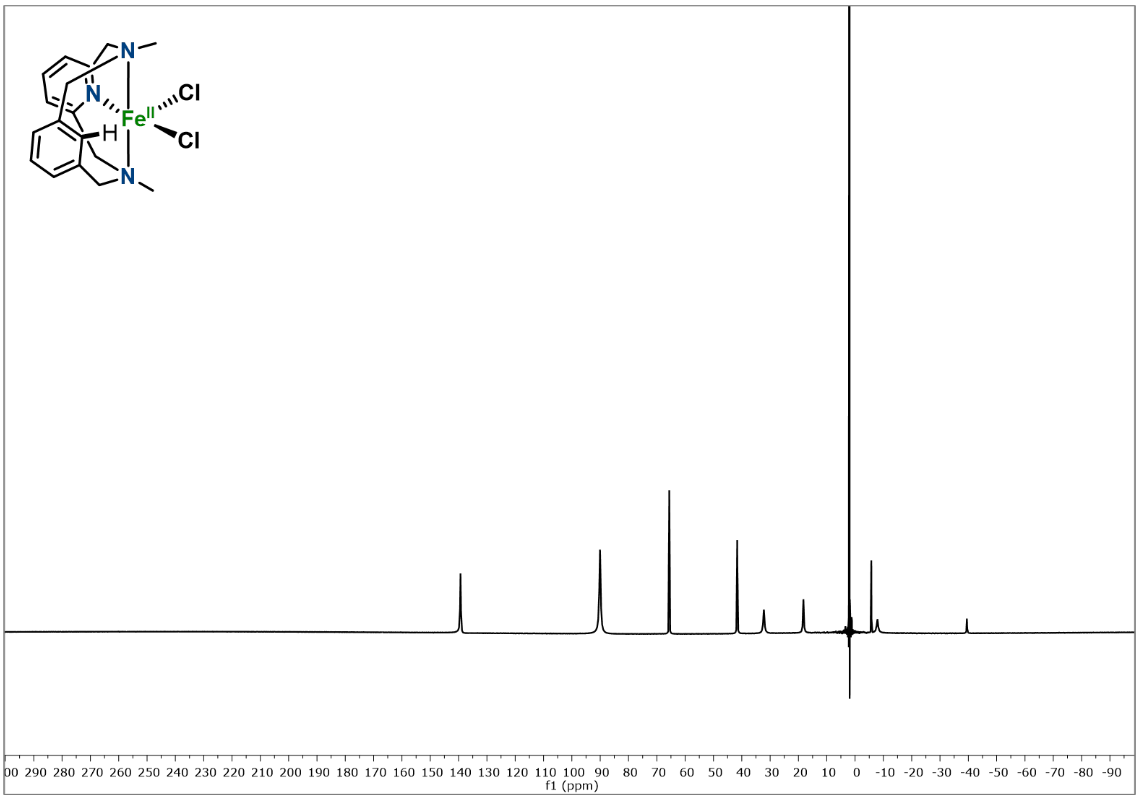

Figure S10. Paramagnetic ^{1}H-NMR spectrum of **1·Cl₂** in CD_3CN at room temperature (400 MHz).

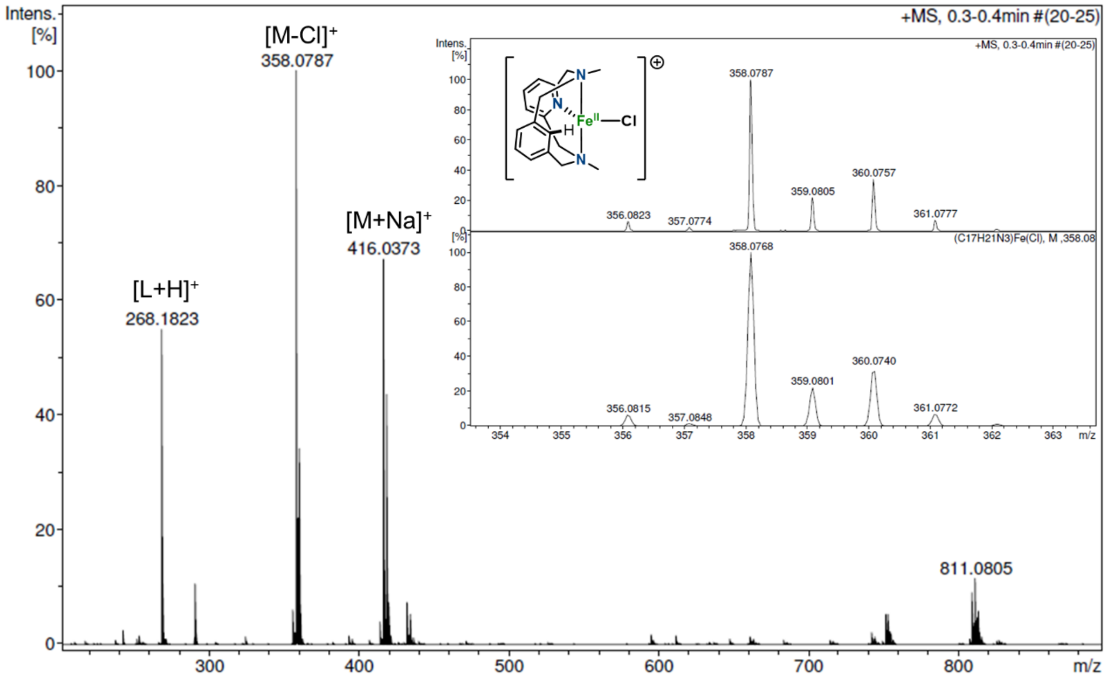

Figure S11. HR-ESI-MS of **1·Cl₂**. Inset: expanded view of the experimental peak at a m/z = 358.0787 with a mass value and an isotopic pattern fully consistent with the peak corresponding to the monocharged [M-Cl$^-$]$^+$ (top) and the expanded view of the corresponding calculated spectrum for this molecular formula (bottom). (M = $C_{17}H_{21}N_3FeCl_2$; L = $C_{17}H_{21}N_3$).

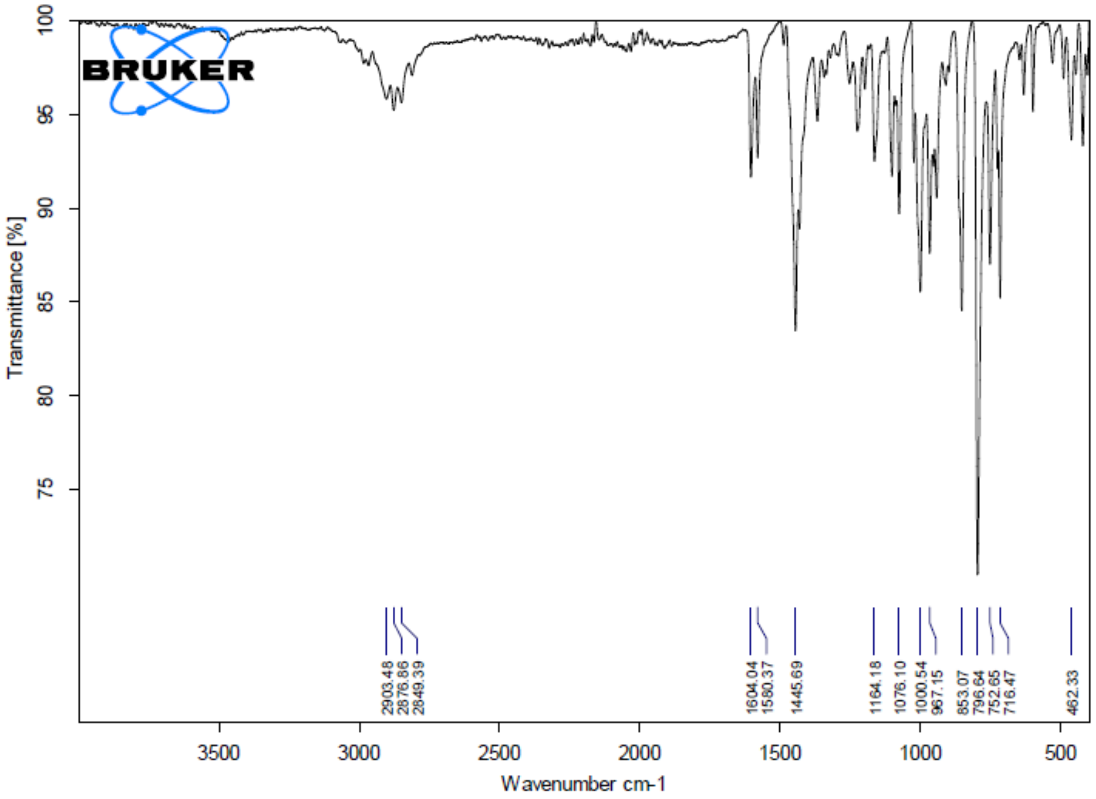

Figure S12. ATR-FT-IR spectrum of **1·Cl₂** at room temperature.

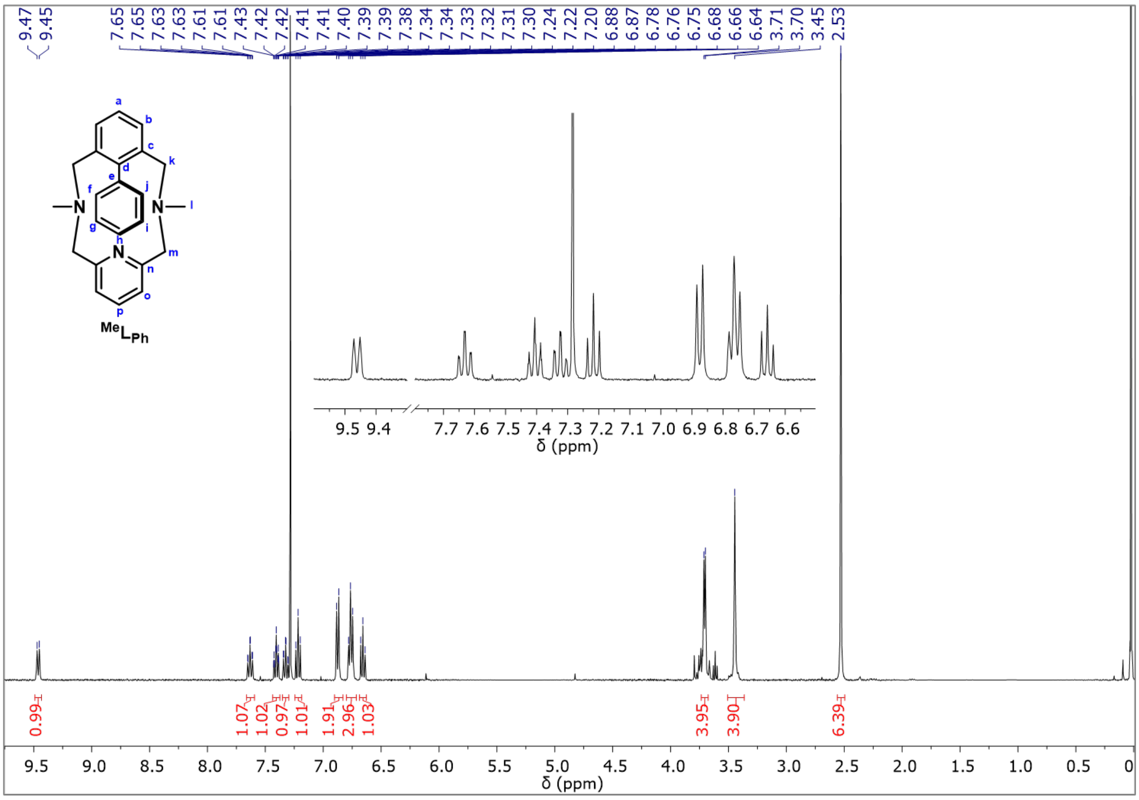

Figure S13. ^{1}H-NMR spectrum of ^{Me}**L**$_{Ph}$ in $CDCl_3$ at room temperature (400 MHz).

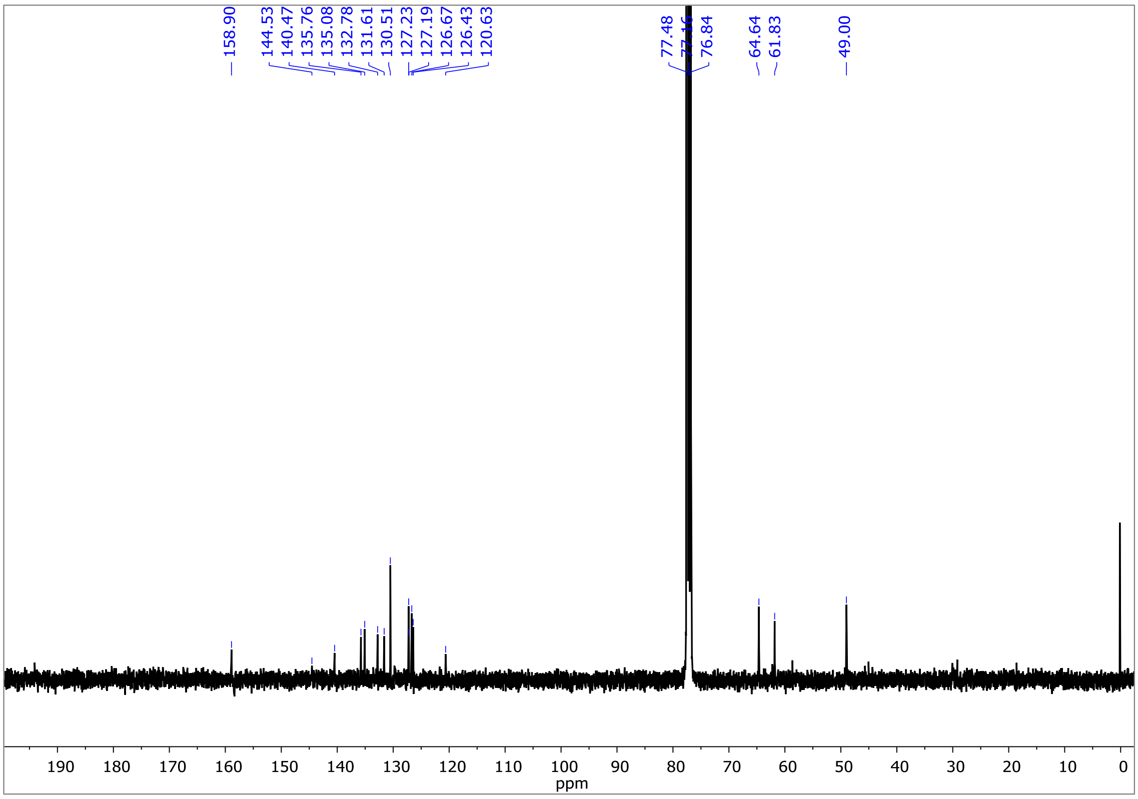

Figure S14. ^{13}C-NMR spectrum of ^{Me}**L**$_{Ph}$ in $CDCl_3$ at room temperature (100 MHz).

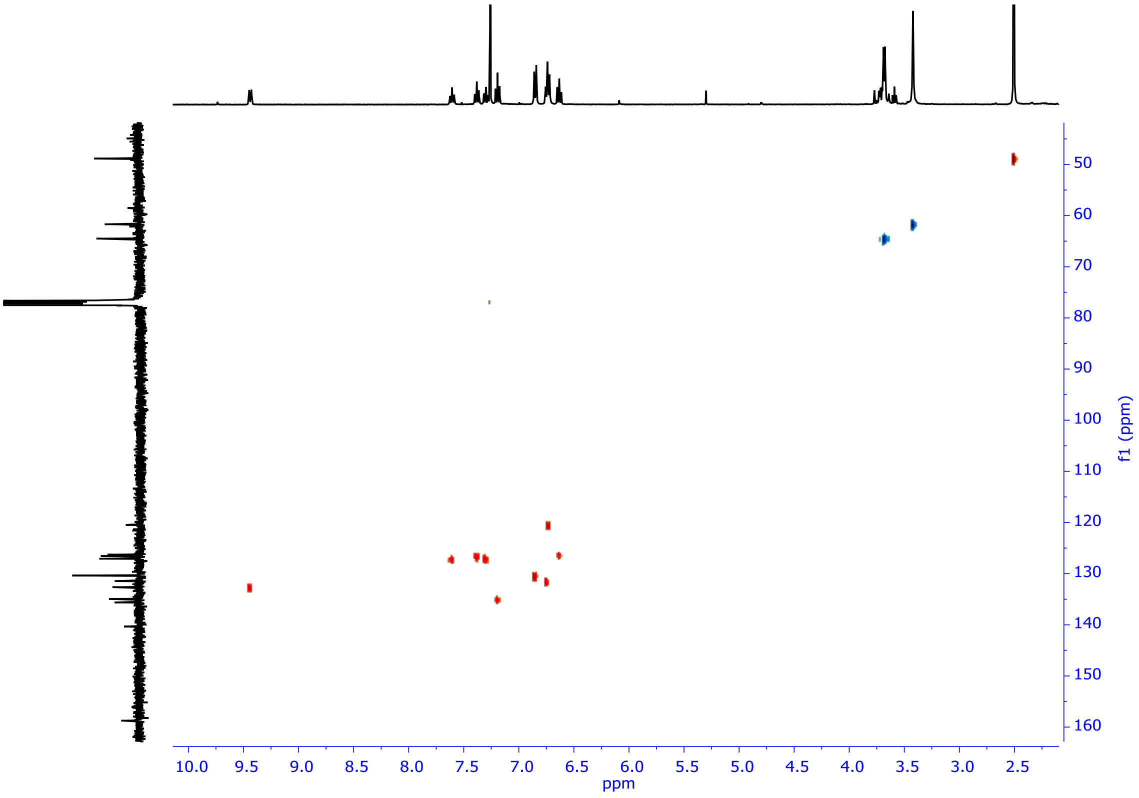

Figure S15. ^{1}H-13C HSQC spectrum of ^{Me}**L**$_{Ph}$ in $CDCl_3$ at room temperature (400 MHz).

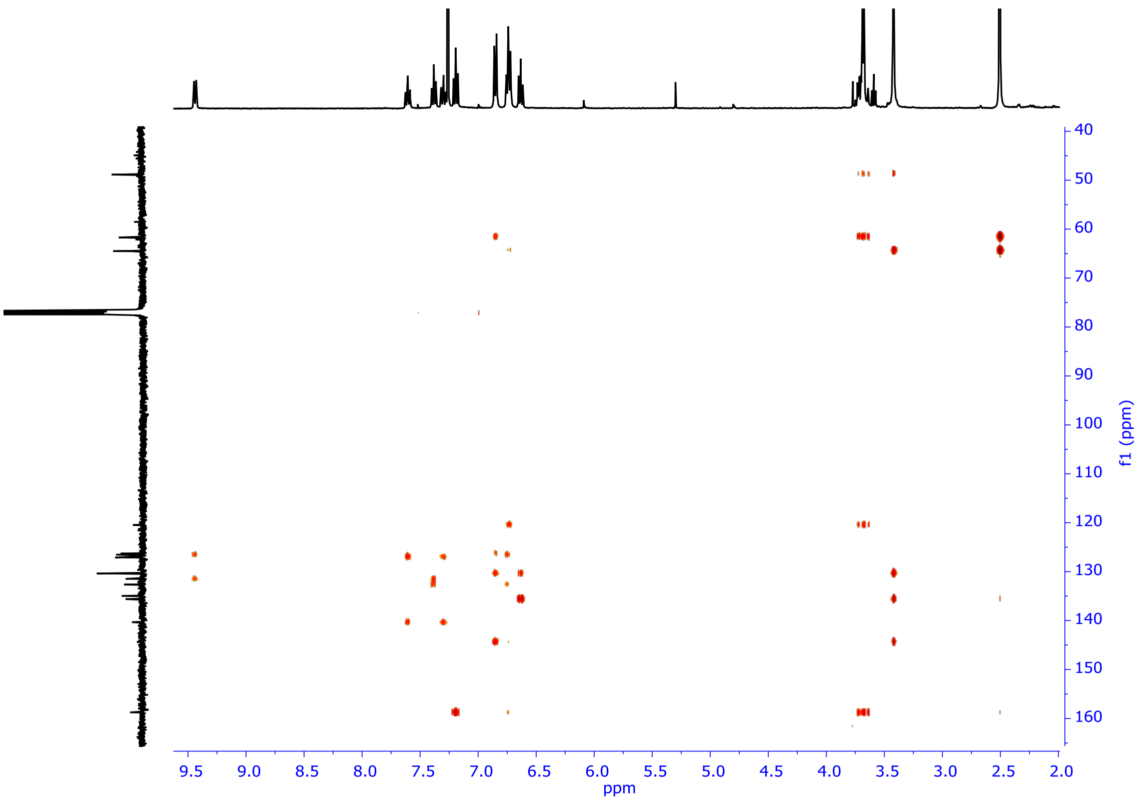

Figure S16. ^{1}H-^{13}C HMBC spectrum of $^{Me}\mathbf{L}_{Ph}$ in $CDCl_3$ at room temperature (400 MHz).

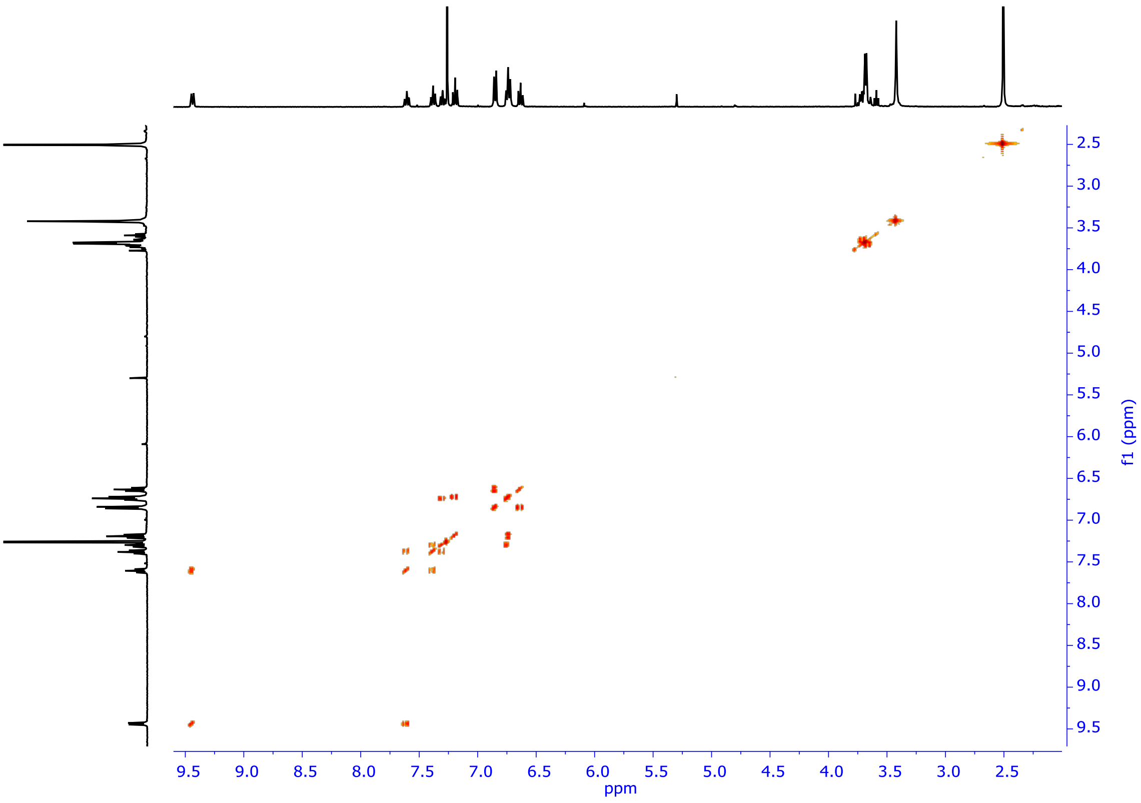

Figure S17. ^{1}H-^{1}H COSY spectrum of $^{Me}\mathbf{L}_{Ph}$ in $CDCl_3$ at room temperature (400 MHz).

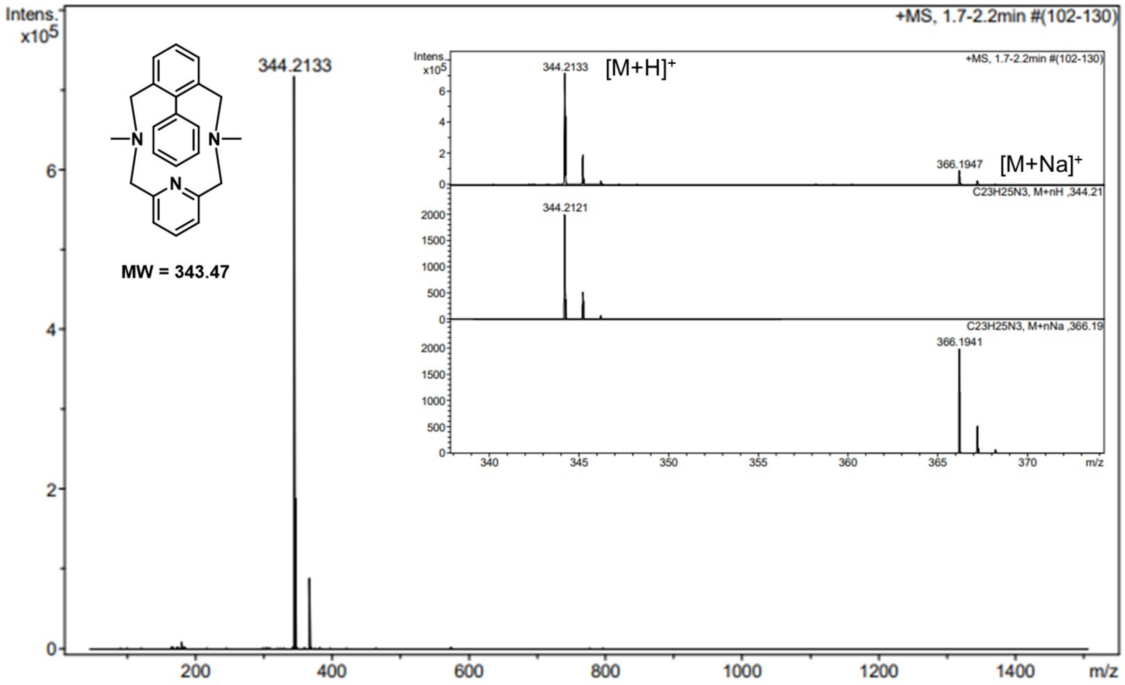

Figure S18. HR-ESI-MS of $^{Me}L_{Ph}$. Inset: expanded view of the experimental peak at a m/z = 344.2133 with a mass value and an isotopic pattern fully consistent with the monocharged $[M+H/Na]^+$ (top) and the corresponding calculated for these molecular formulas (middle $[M+H]^+$, bottom $[M+Na]^+$). (M = $C_{23}H_{25}N_3$).

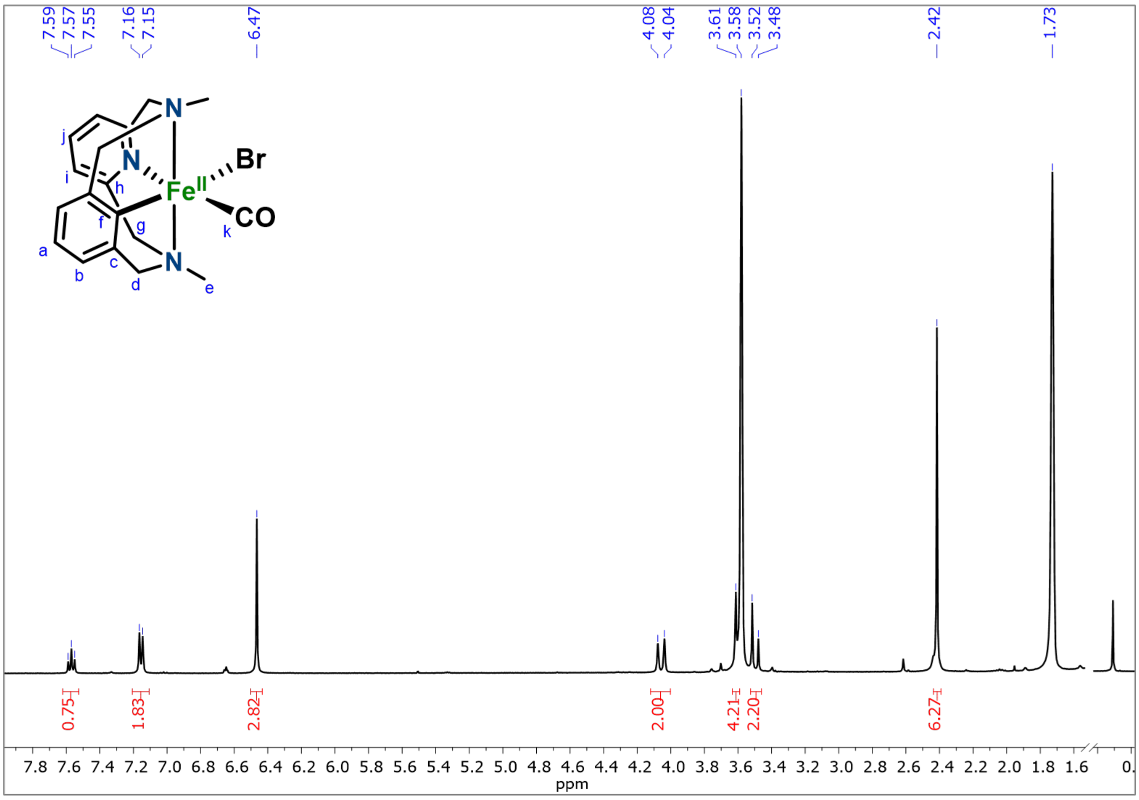

Figure S19. ^{1}H-NMR spectrum of $\mathbf{1^{Me}}$ in THF-d$_8$ at room temperature (400 MHz).

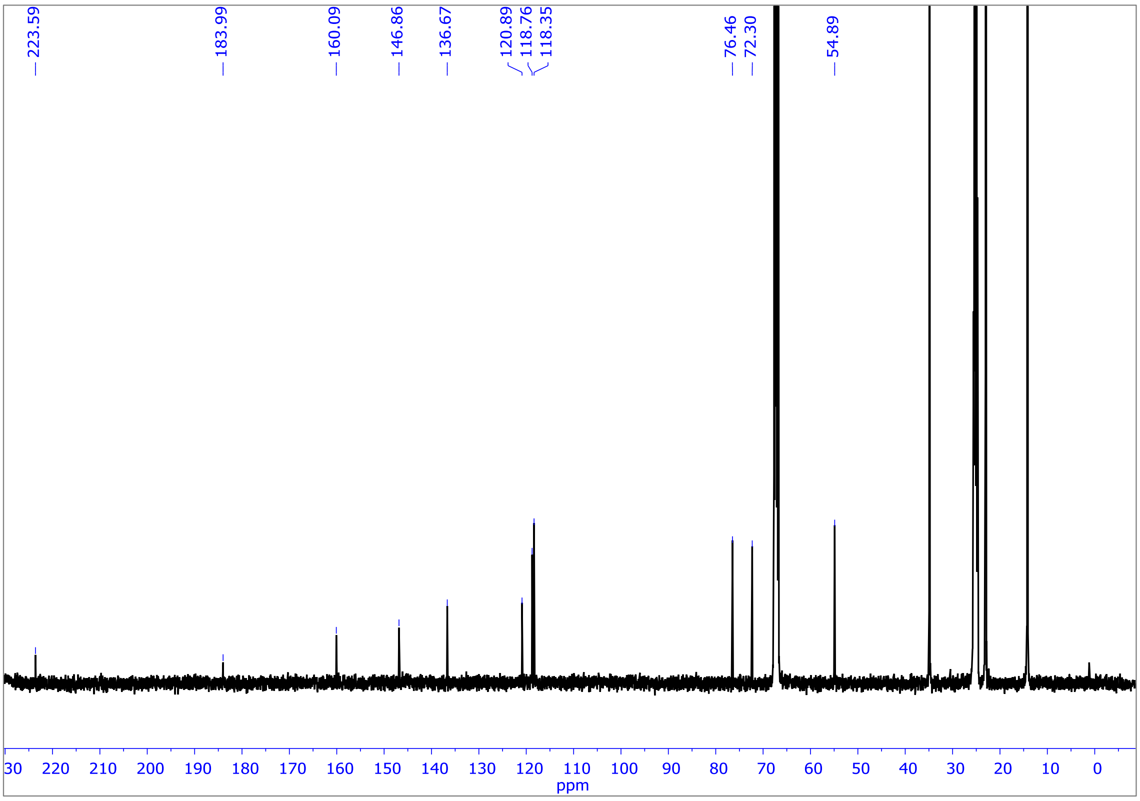

Figure S20. ^{13}C-NMR spectrum of $\mathbf{1}^{Me}$ in THF-d_8 at room temperature (100 MHz).

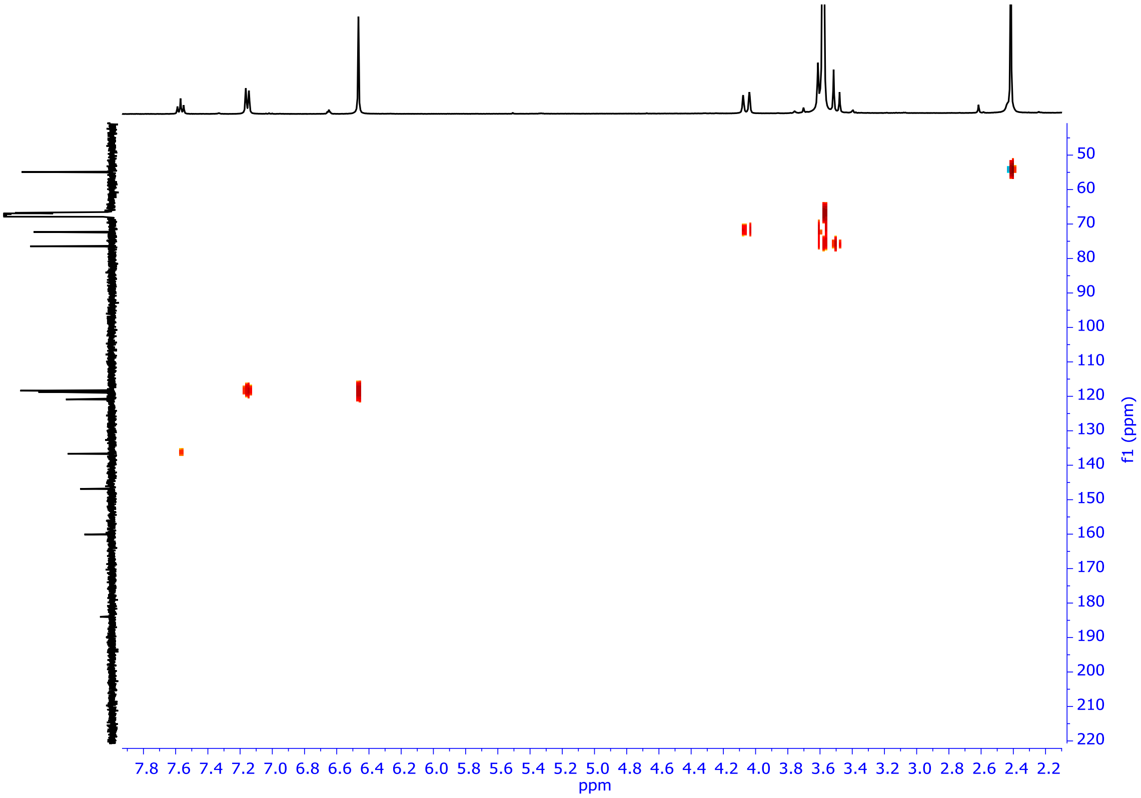

Figure S21. ^{1}H-^{13}C HSQC spectrum of $\mathbf{1}^{Me}$ in THF-d_8 at room temperature (400 MHz).

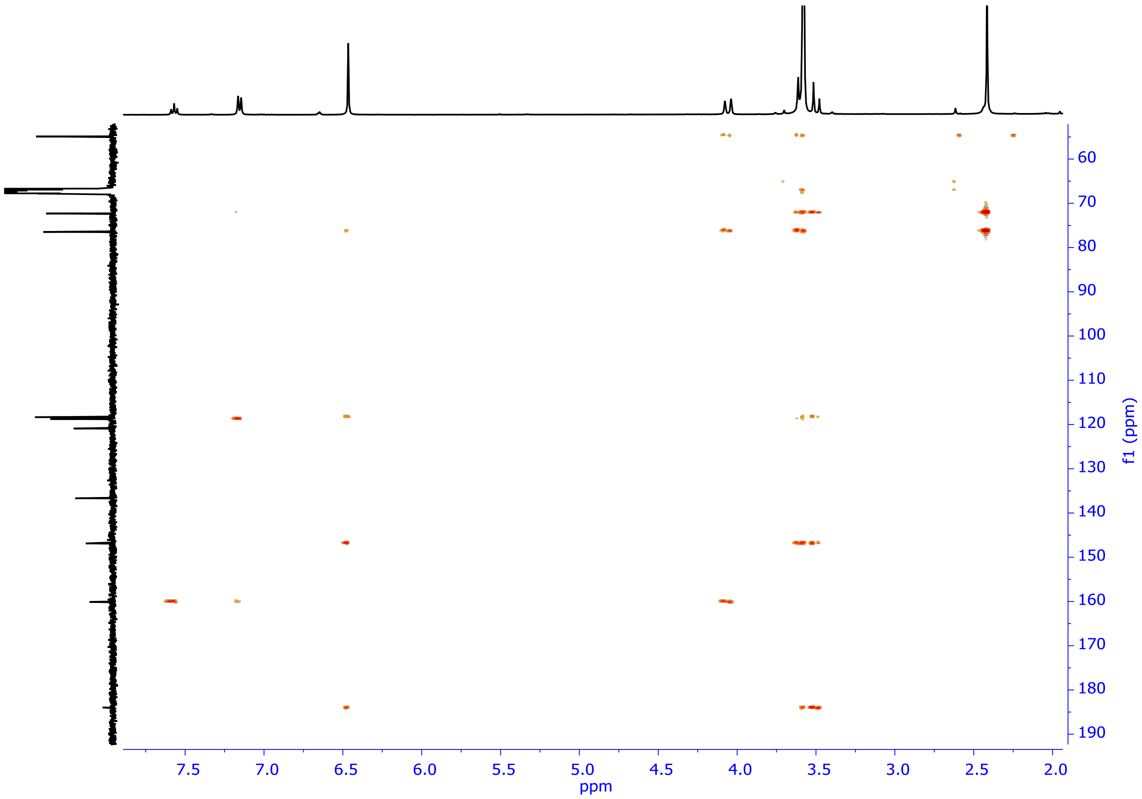

Figure S22. ^{1}H-^{13}C HMBC spectrum of $\mathbf{1}^{Me}$ in THF-d_8 at room temperature (400 MHz).

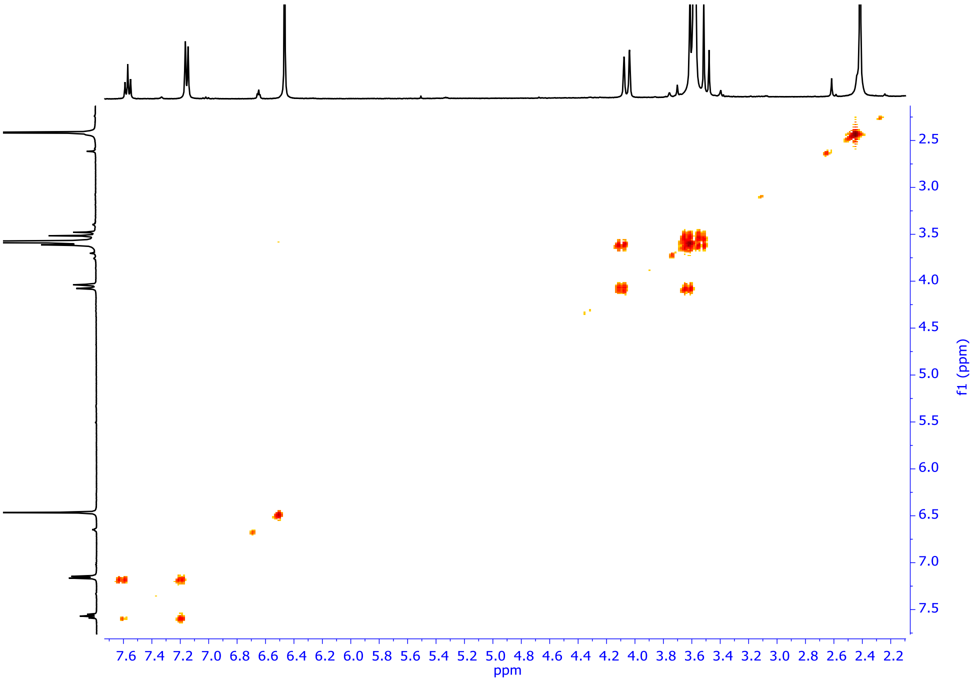

Figure S23. ^{1}H-^{1}H COSY spectrum of $\mathbf{1}^{Me}$ in THF-d_8 at room temperature (400 MHz).

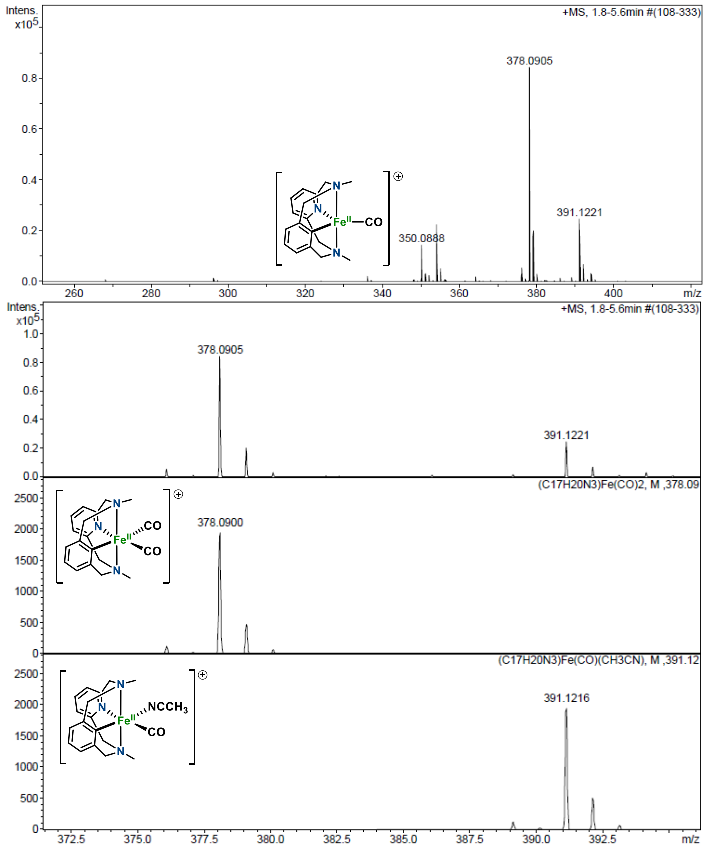

Figure S24. HR-ESI-MS of $\mathbf{1}^{\mathbf{Me}}$. Expanded view of the experimental peaks with mass value and isotopic pattern fully consistent with the monocharged $[C_{19}H_{20}N_3O_2Fe]^+ = 378.0905$ and $[C_{20}H_{23}N_4OFe]^+ = 391.1221$ (top) and the corresponding calculated for these molecular formulas (middle $[C_{19}H_{20}N_3O_2Fe]^+$, bottom $[C_{20}H_{23}N_4OFe]^+$).

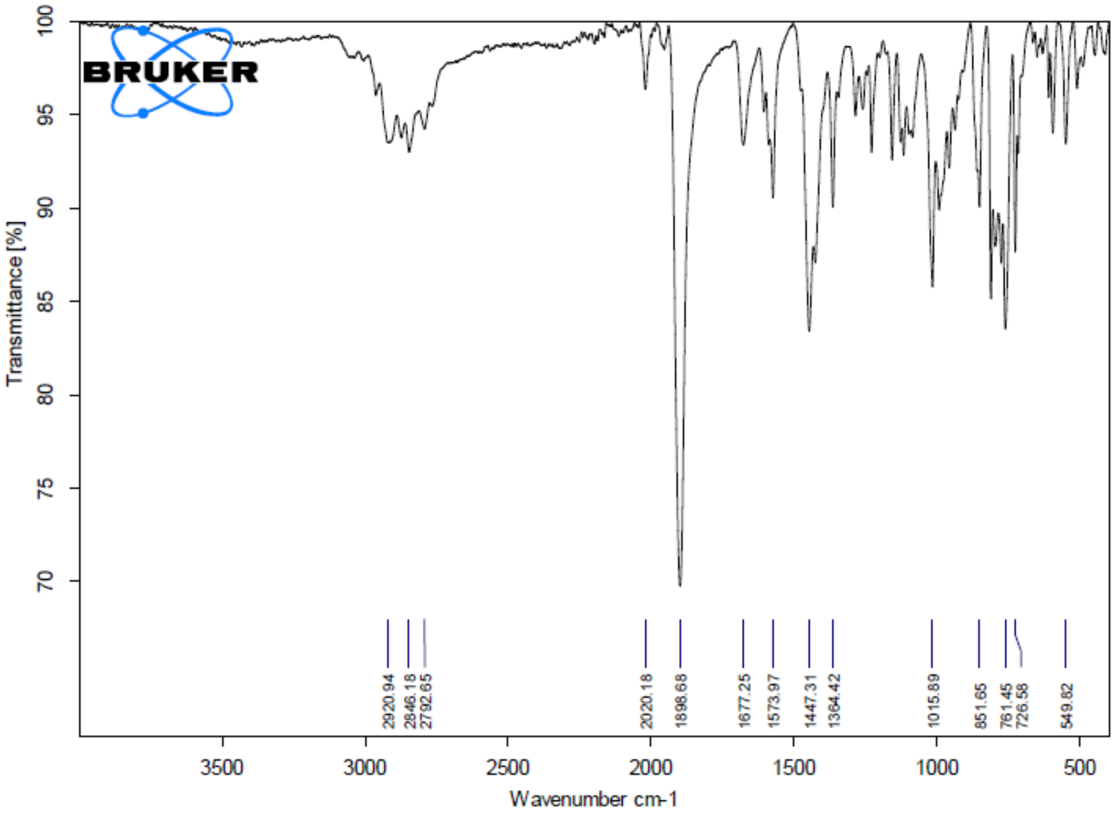

Figure S25. ATR-FT-IR spectrum of $\mathbf{1}^{\mathbf{Me}}$ at room temperature.

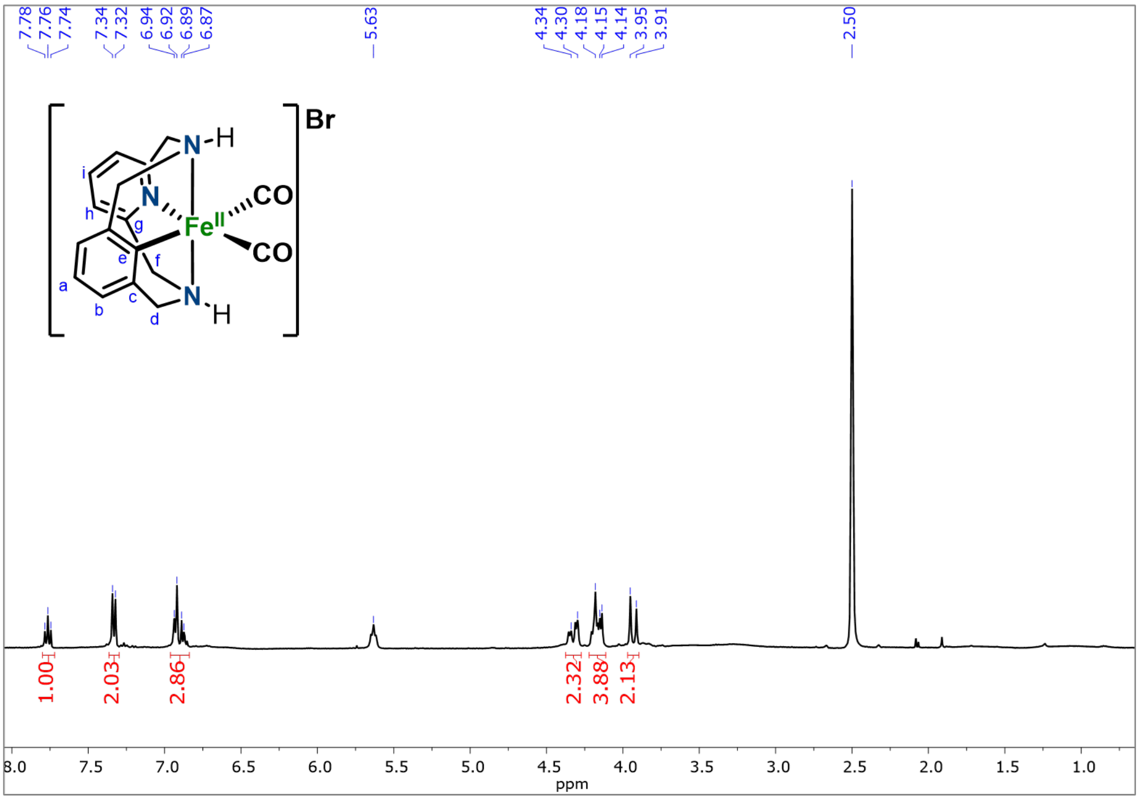

Figure S26. ^{1}H-NMR spectrum of $\mathbf{1^H}$ in DMSO-d_6 at room temperature (400 MHz).

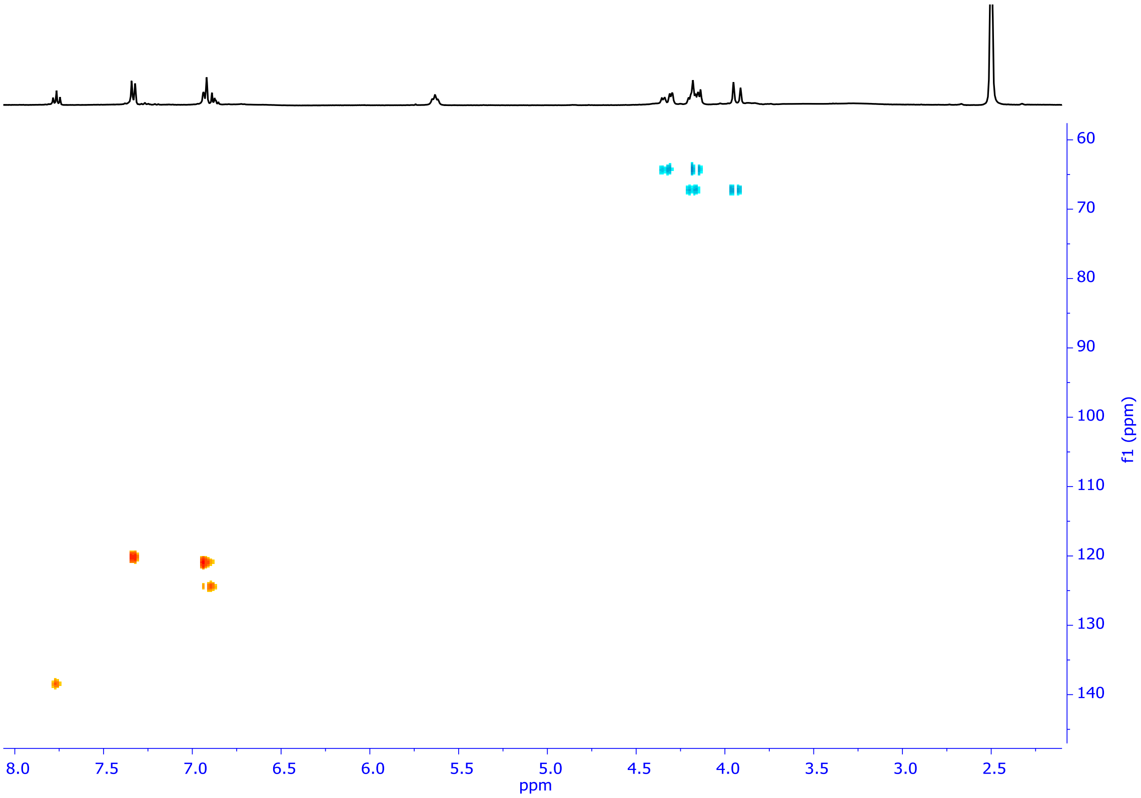

Figure S27. ^{1}H-^{13}C HSQCed spectrum of $\mathbf{1^H}$ in DMSO-d_6 at room temperature (400 MHz).

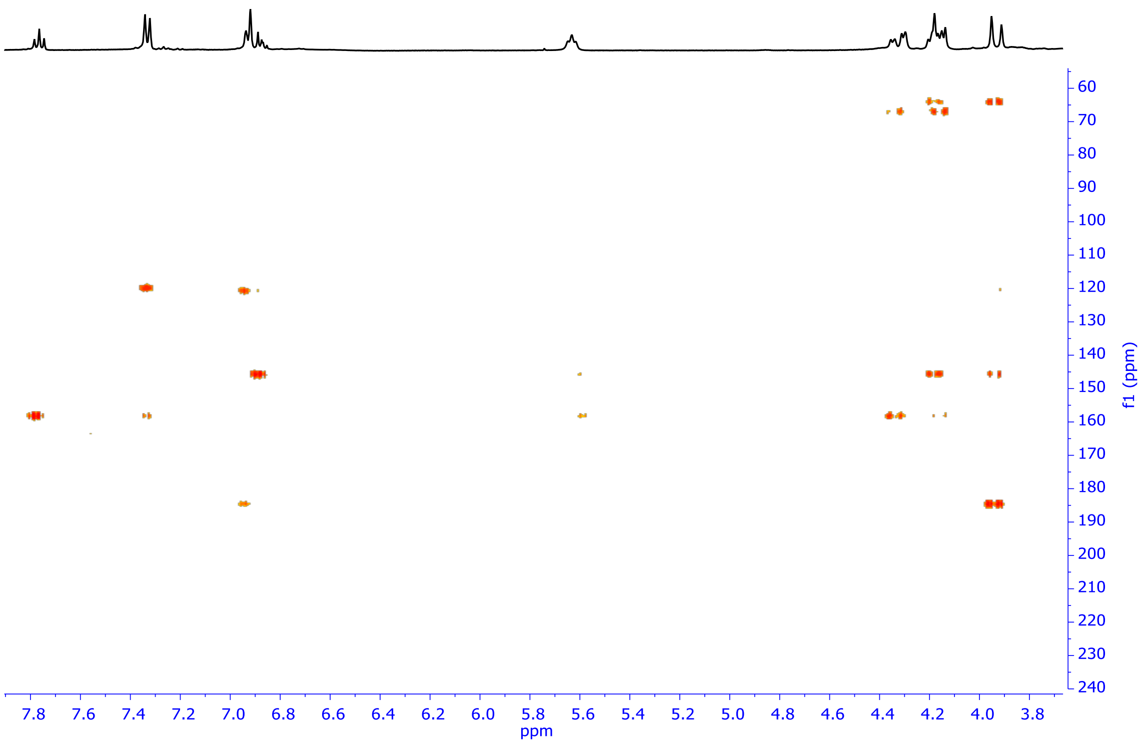

Figure S28. ^{1}H-^{13}C HMBC spectrum of 1^{H} in DMSO-d_6 at room temperature (400 MHz).

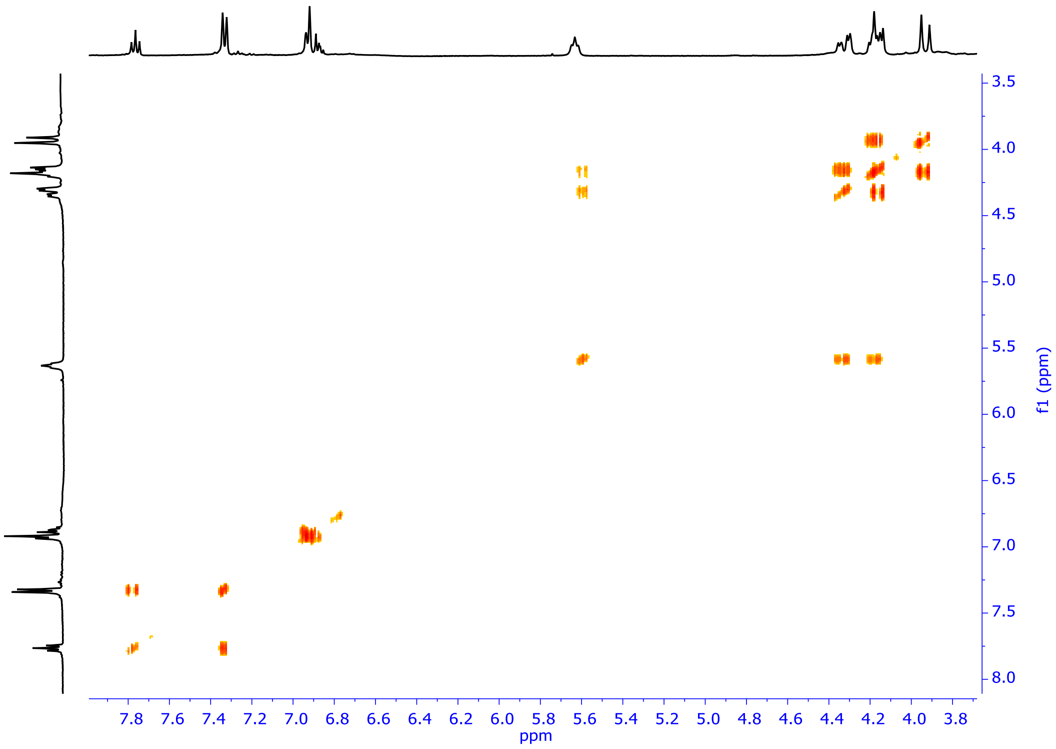

Figure S29. ^{1}H-^{1}H COSY spectrum of 1^{H} in DMSO-d_6 at room temperature (400 MHz).

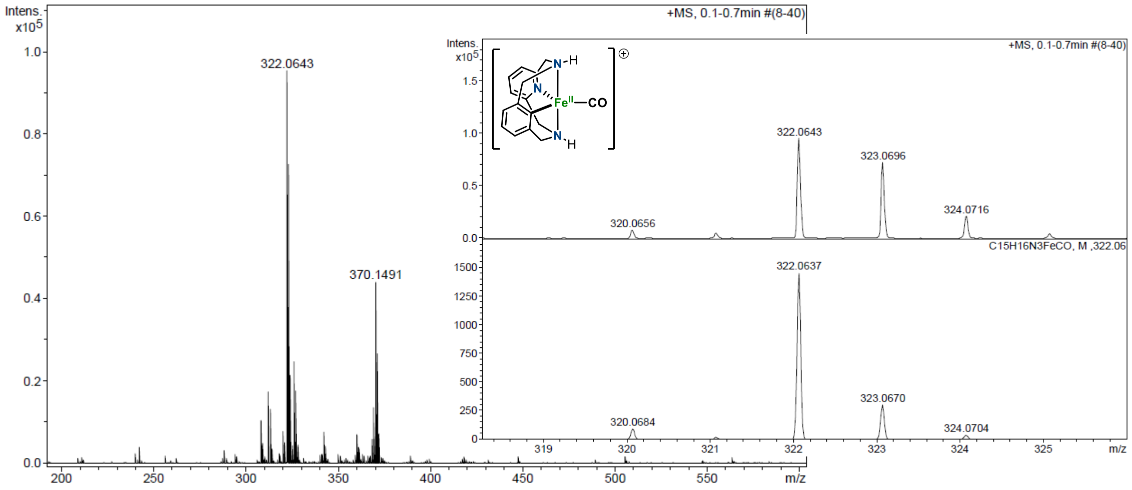

Figure S30. HR-ESI-MS of $\mathbf{1}^{\mathrm{H}}$. Inset: expanded view of the experimental peak with mass value and isotopic pattern fully consistent with the monocharged $[C_{16}H_{16}N_3OFe]^+$ = 322.0643 (top) and the corresponding calculated for this molecular formula (bottom).

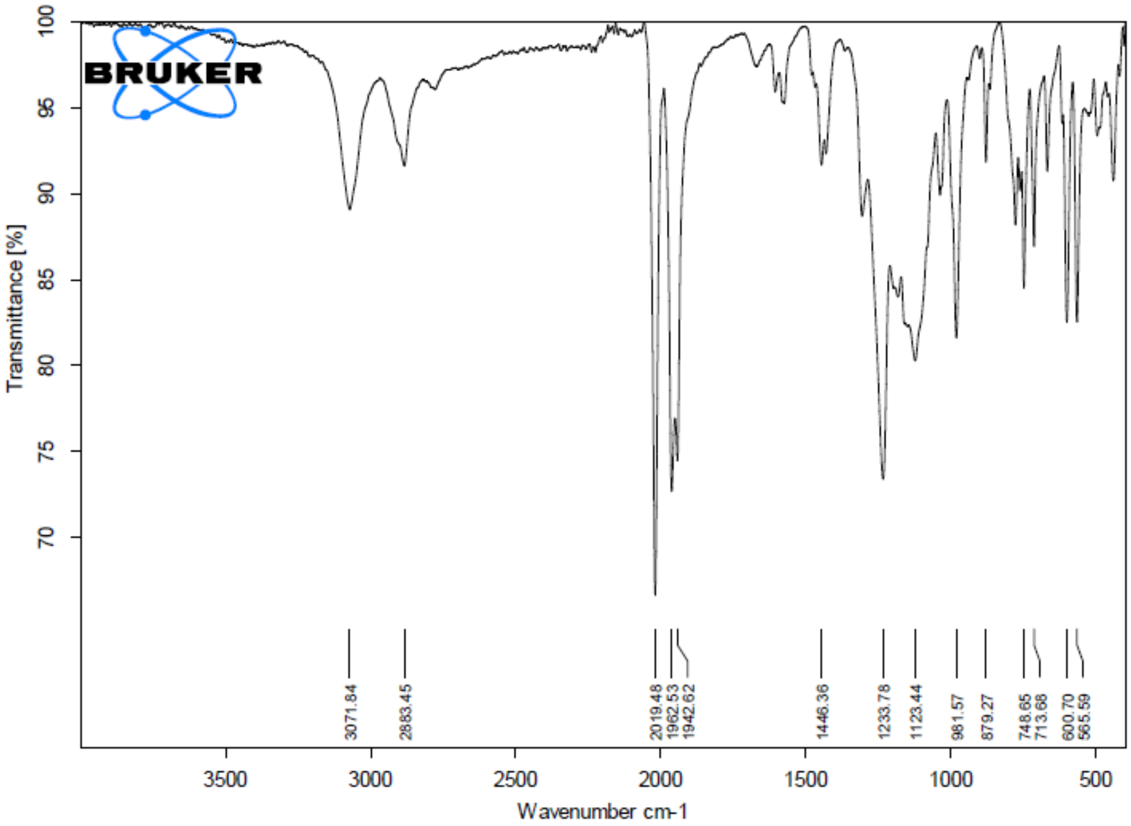

Figure S31. ATR-FT-IR spectrum of $\mathbf{1}^{\mathrm{H}}$ at room temperature.

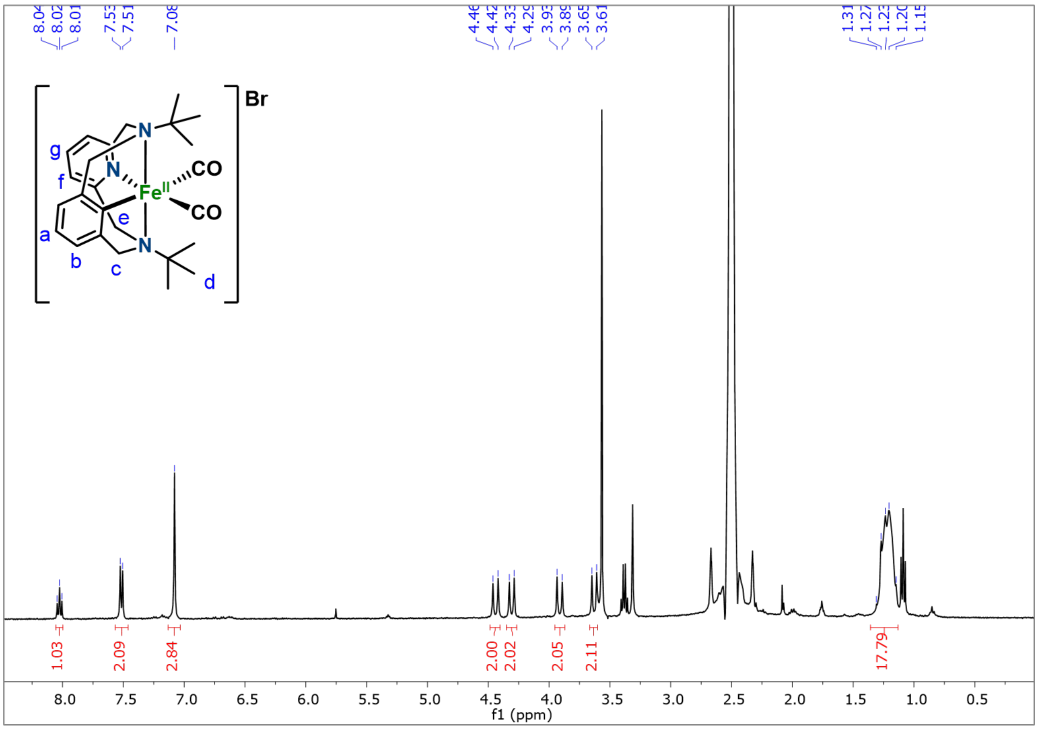

Figure S32. ^{1}H-NMR spectrum of $\mathbf{1}^{tBu}$ in DMSO-d$_6$ at room temperature (400 MHz).

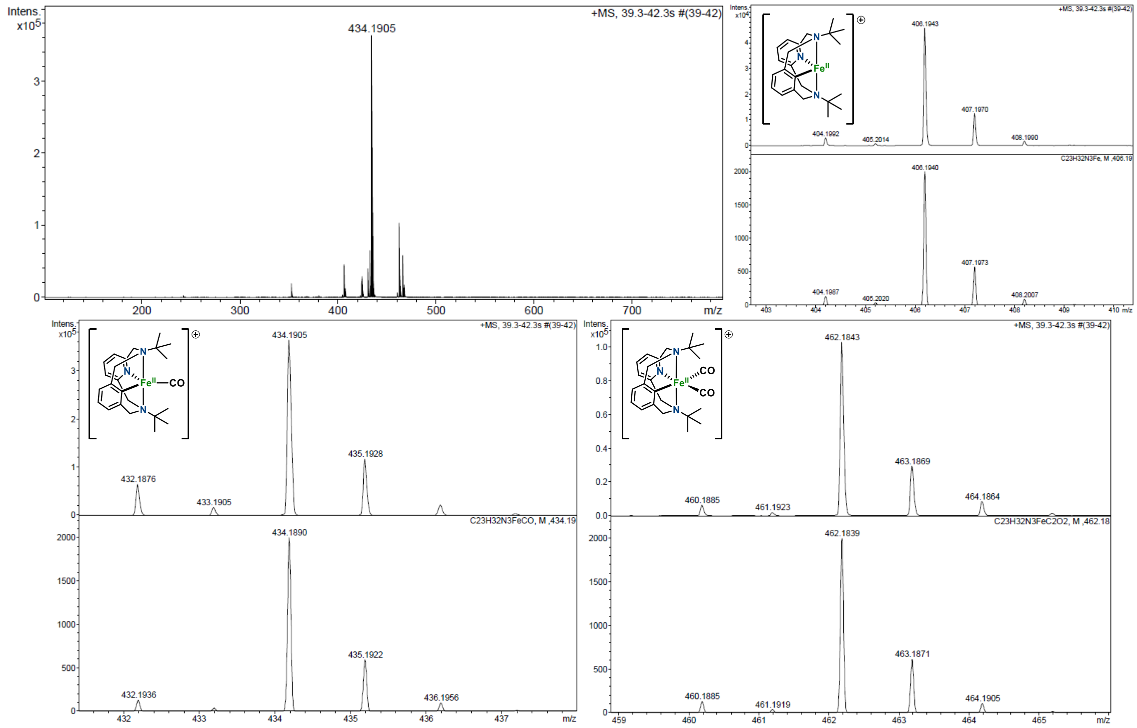

Figure S33. Top left: HR-ESI-MS of $\mathbf{1}^{tBu}$. Top right: expanded view of the experimental peak at m/z = 406.1943 with a mass value and an isotopic pattern fully consistent with the monocharged $[C_{23}H_{32}N_3Fe]^+$ (top) and the corresponding calculated for this molecular formula (bottom). Bottom left: expanded view of the experimental peak at m/z = 434.1905 with a mass value and an isotopic pattern fully consistent with the monocharged $[C_{24}H_{32}N_3OFe]^+$ (top) and the corresponding calculated for this molecular formula (bottom). Bottom right: expanded view of the experimental peak at m/z = 462.1843 with a mass value and an isotopic pattern fully consistent with the monocharged $[C_{25}H_{32}N_3O_2Fe]^+$ (top) and the corresponding calculated for this molecular formula (bottom).

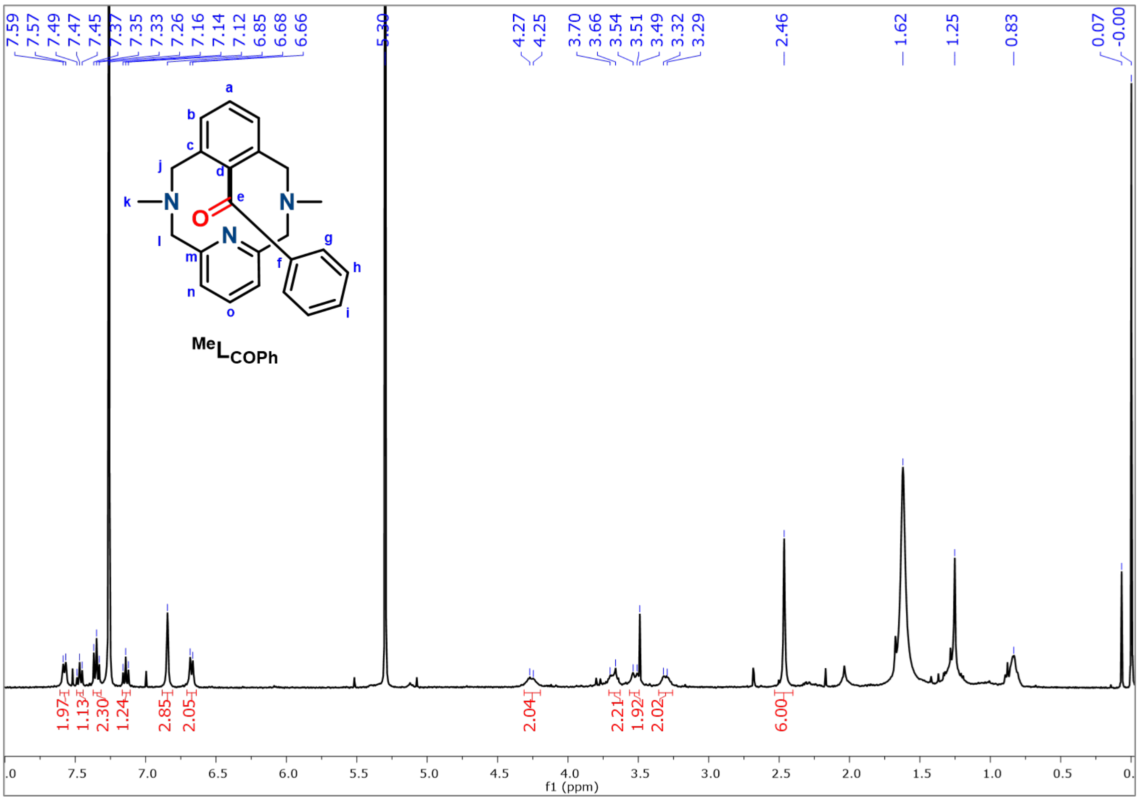

Figure S34. ^{1}H-NMR spectrum of ^{Me}L$_{COPh}$ in $CDCl_3$ at room temperature (400 MHz).

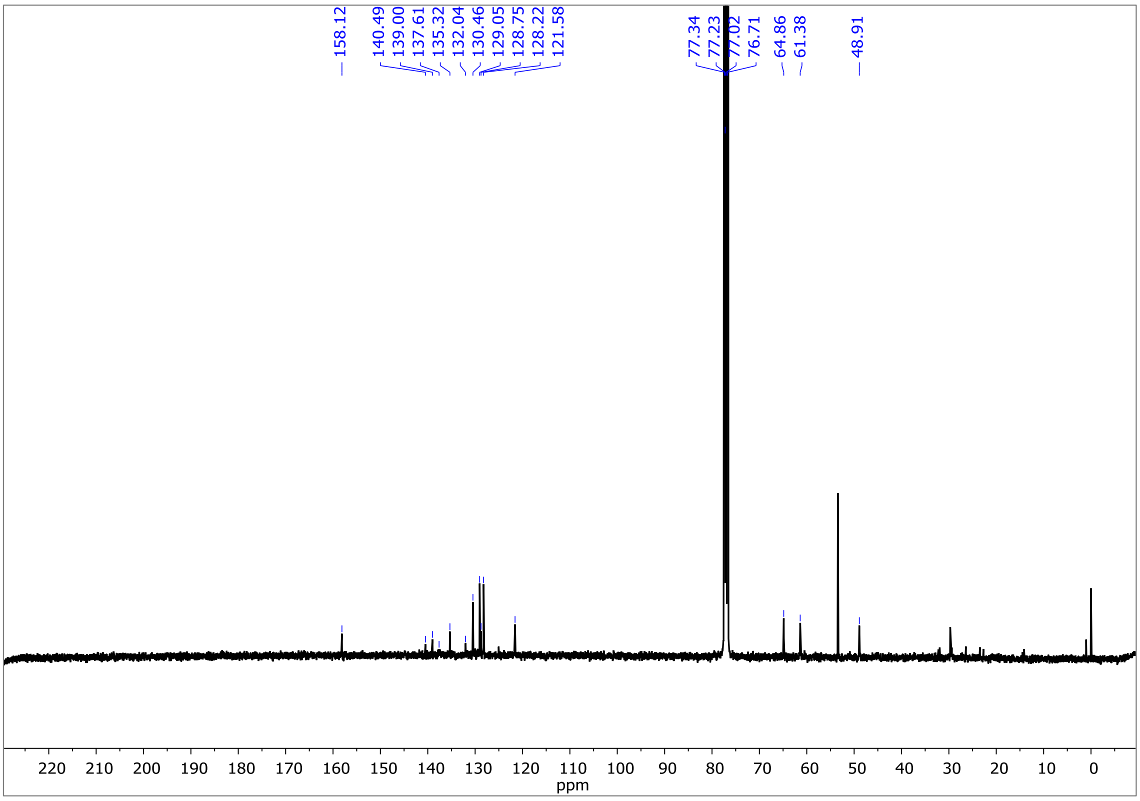

Figure S35. ^{13}C-NMR spectrum of ^{Me}L$_{COPh}$ in $CDCl_3$ at room temperature (400 MHz).

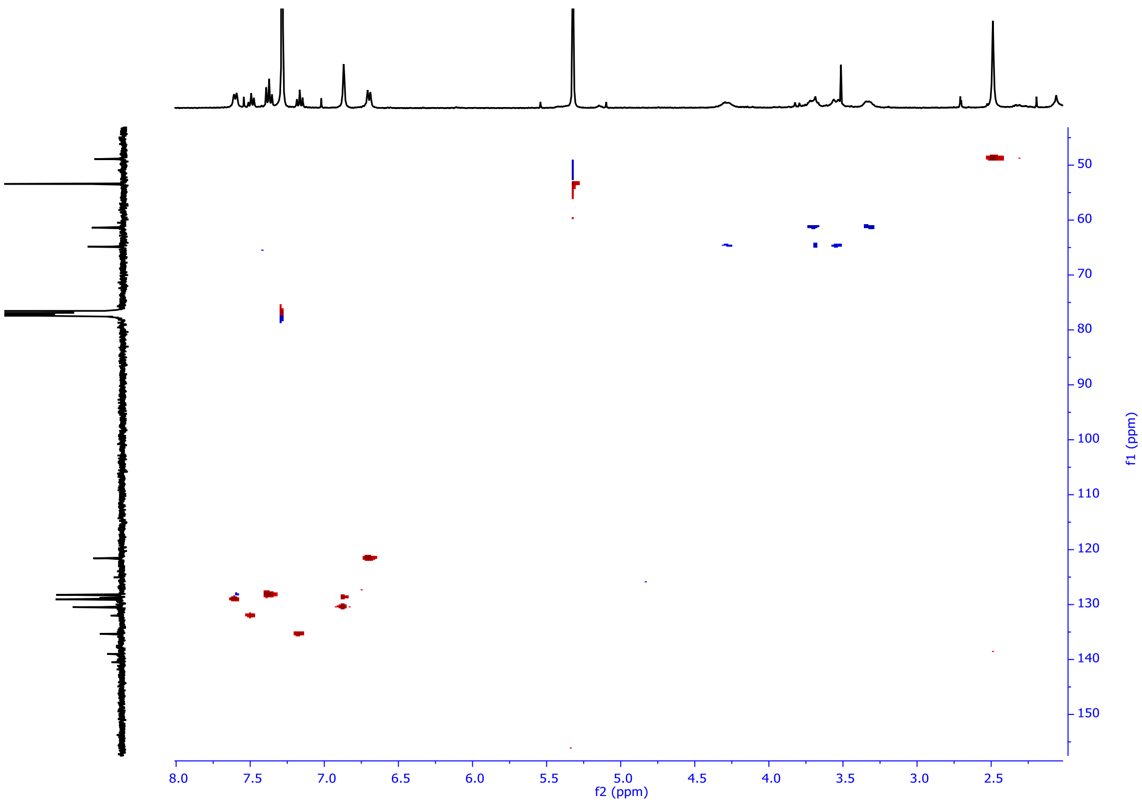

Figure S36. ^{1}H-^{13}C HSQCed spectrum of $^{Me}\mathbf{L}_{COPh}$ in $CDCl_3$ at room temperature (400 MHz).

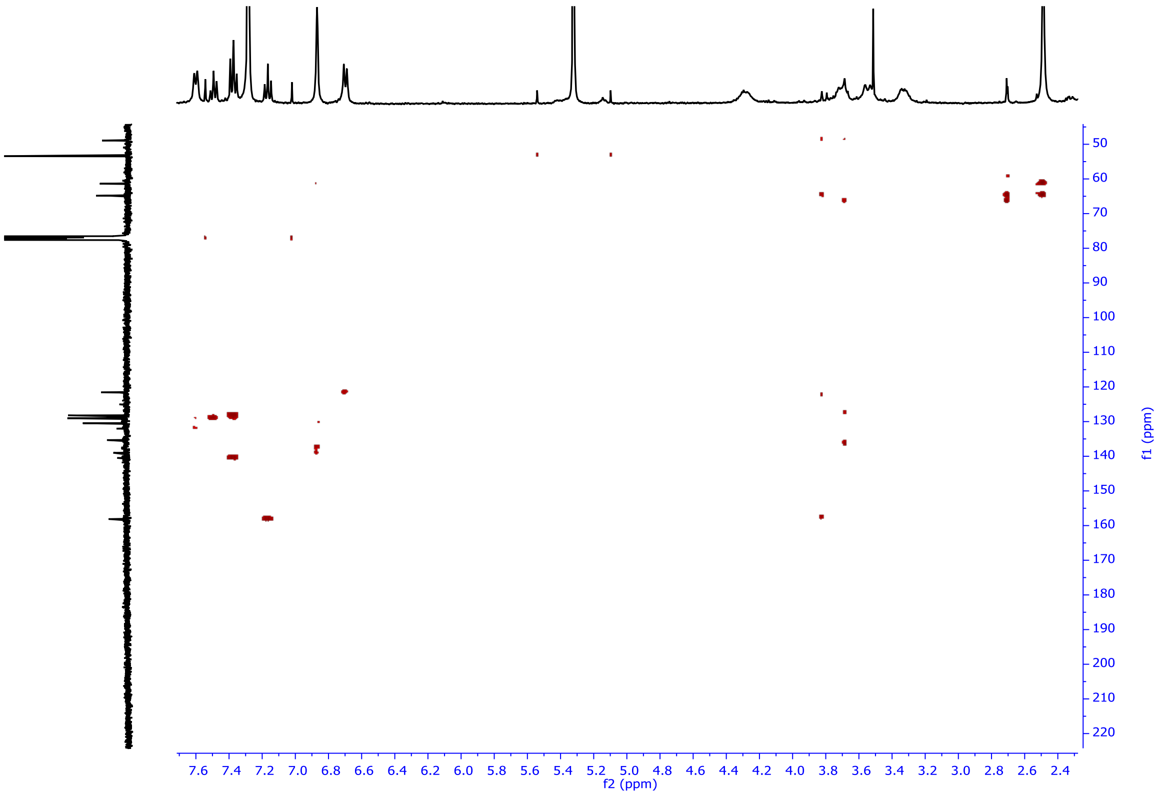

Figure S37. ^{1}H-^{13}C HMBC spectrum of $^{Me}\mathbf{L}_{COPh}$ in $CDCl_3$ at room temperature (400 MHz).

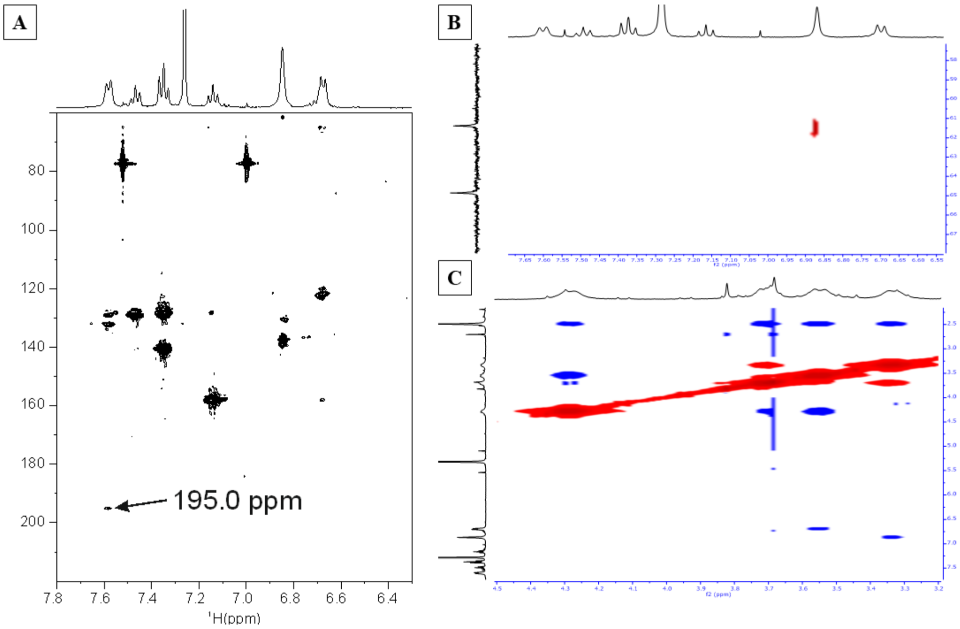

Figure S38. A: ^{1}H-^{13}C HMBC spectrum of ^{Me}**L**$_{COPh}$ in $CDCl_3$ at room temperature (400 MHz), ampliation of the aromatic region for detection of the ketone CO. The presence of chemical exchange dynamics in combination with relaxation properties of carbonyl nuclei do not allow to detect CO with proper sensitivity. The presence of CO band in IR spectrum enforce us to optimize a HMBC spectra to improve the sensitivity provided by ^{13}C NMR spectra and be able to assign the carbonyl nucleus using conventional HMBC without low-pass filter spectrum optimized with a interscan delay of 3.5s. B: ^{1}H-^{13}C HMBC spectrum of ^{Me}**L**$_{COPh}$ in $CDCl_3$ at room temperature (400 MHz), ampliation of the aromatic (^{1}H) and aliphatic (^{13}C) regions to assign the benzylic positions. C: ^{1}H-^{1}H NOESY spectrum of ^{Me}**L**$_{COPh}$ in $CDCl_3$ at room temperature (400 MHz), ampliation of the aliphatic (f1) region to assign and further confirm the benzylic positions.

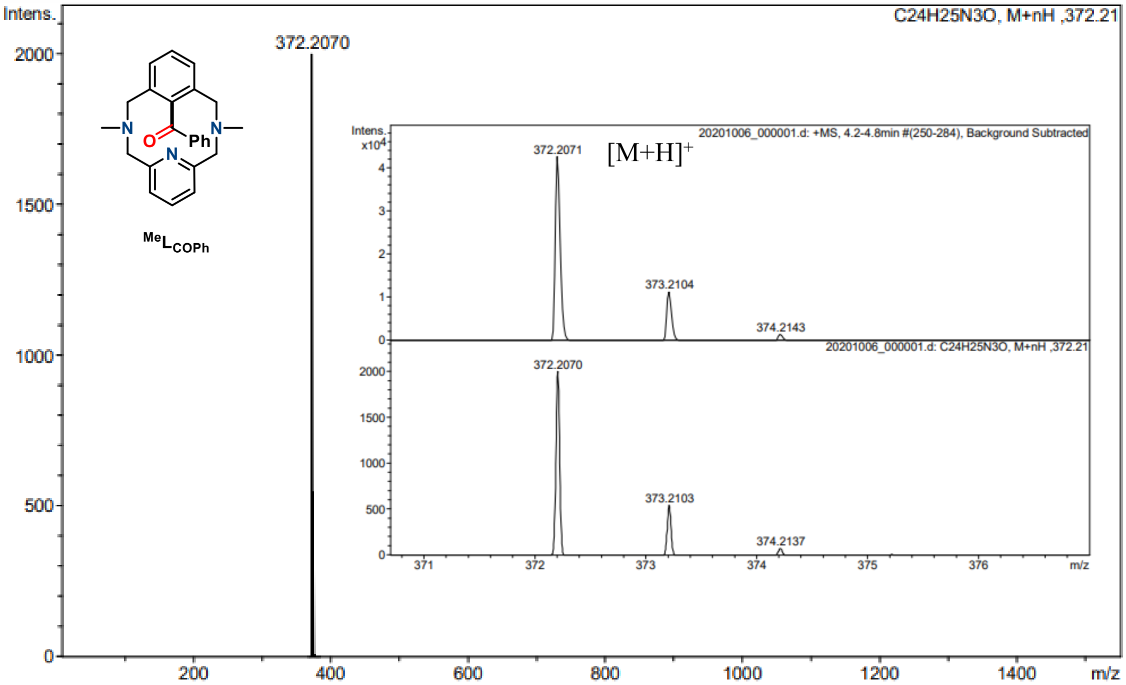

Figure S39. HR-ESI-MS of ^{Me}**L**$_{COPh}$. Inset: expanded view of the experimental peak at m/z = 372.2070 with a mass value and an isotopic pattern fully consistent with $[M+H]^+$ (top) and the corresponding calculated for this molecular formula (bottom). (M = $C_{24}H_{25}N_3O$).

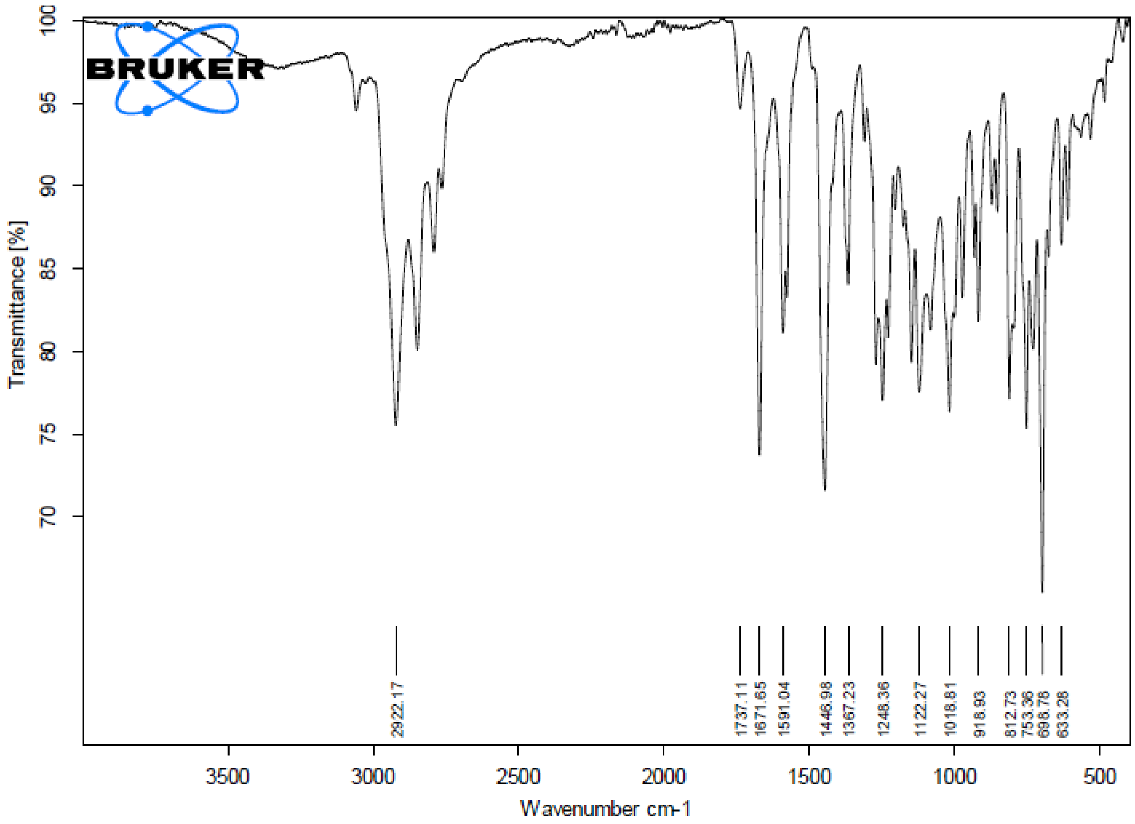

Figure S40. ATR-FT-IR spectrum of $^{Me}L_{COPh}$ at room temperature.

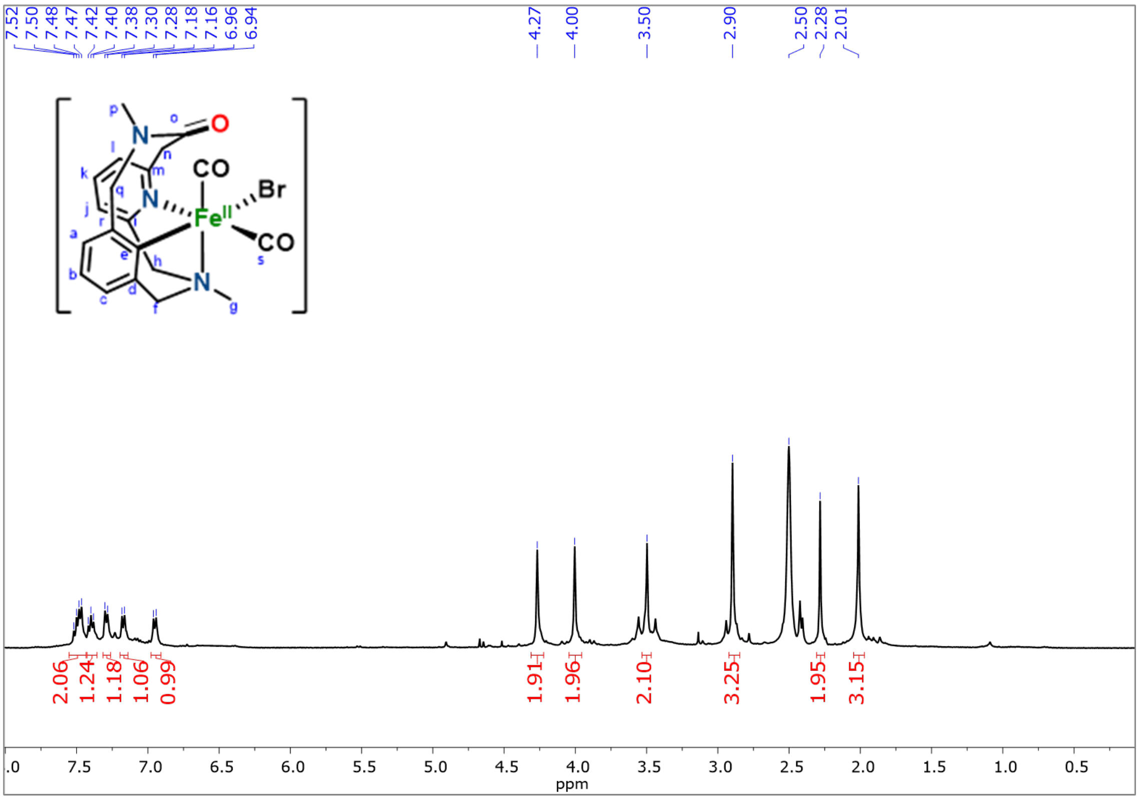

Figure S41. ^{1}H-NMR spectrum of **2^{Me}(CO)** in DMSO-d_6 at room temperature (400 MHz).

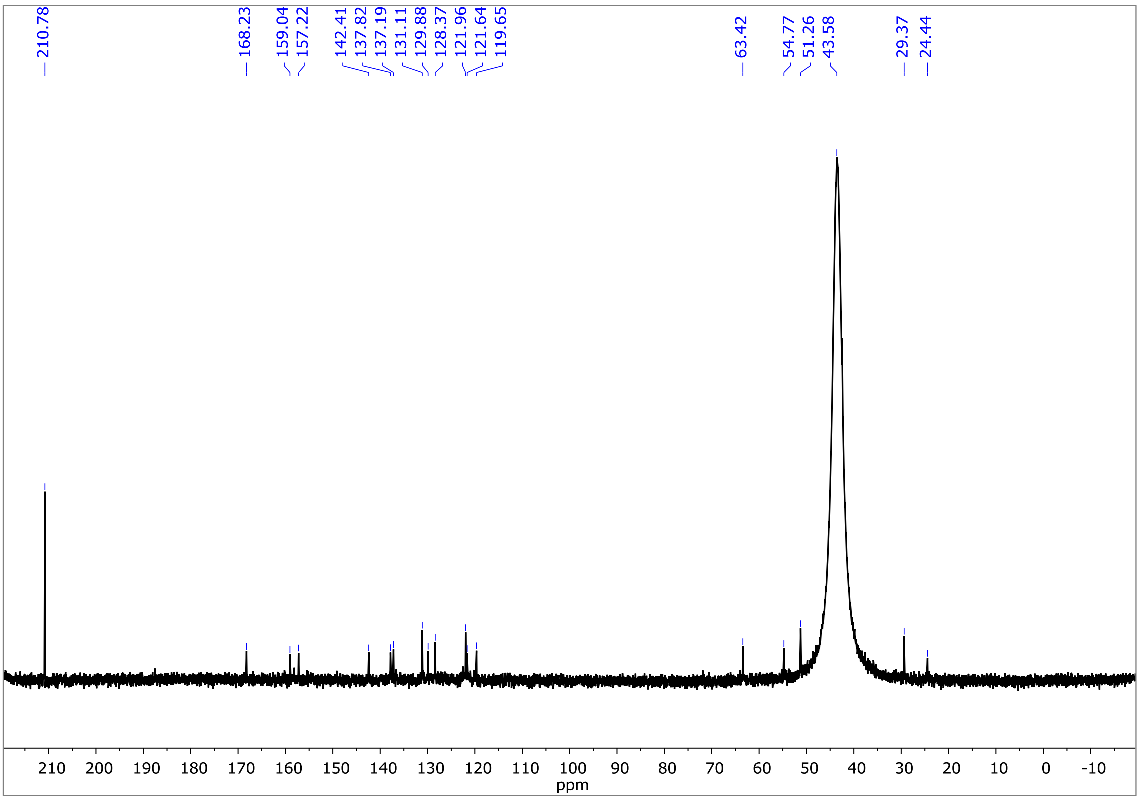

Figure S42. ^{13}C-NMR spectrum of **2^{Me}(CO)** in DMSO-d_6 at room temperature (100 MHz).

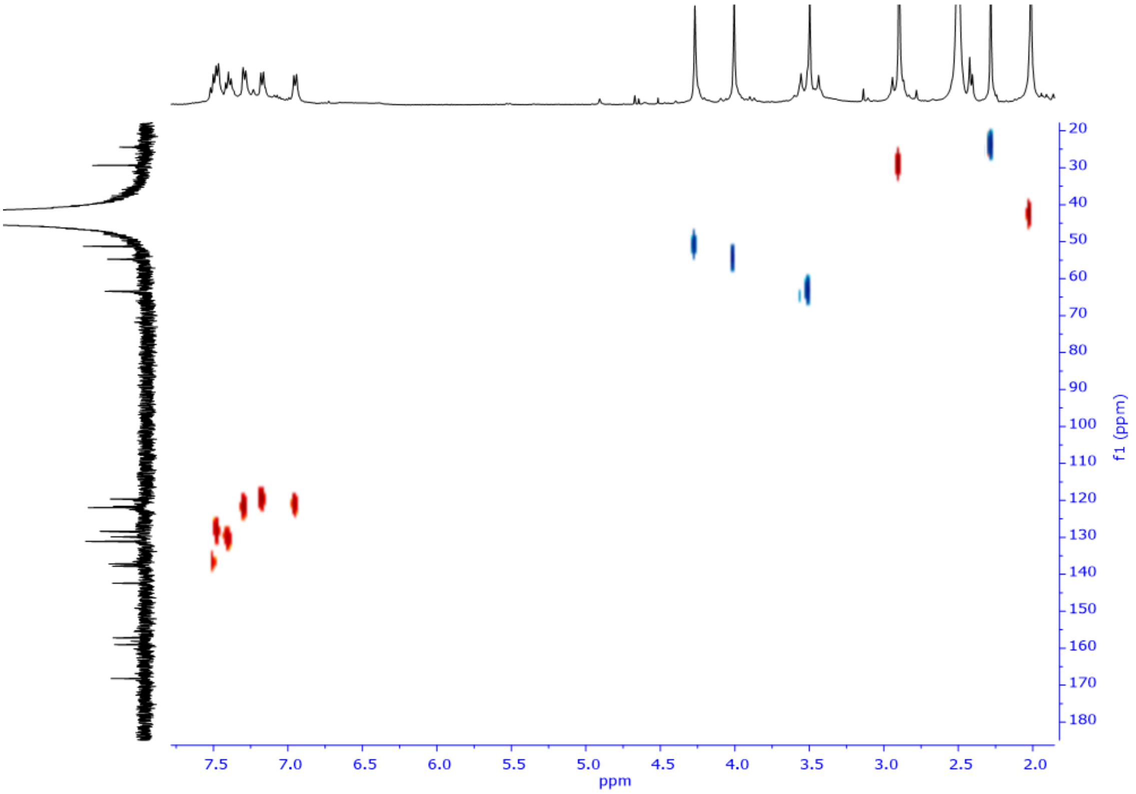

Figure S43. ^{1}H-^{13}C HSQC spectrum of **2^{Me}(CO)** in DMSO-d_6 at room temperature (400 MHz).

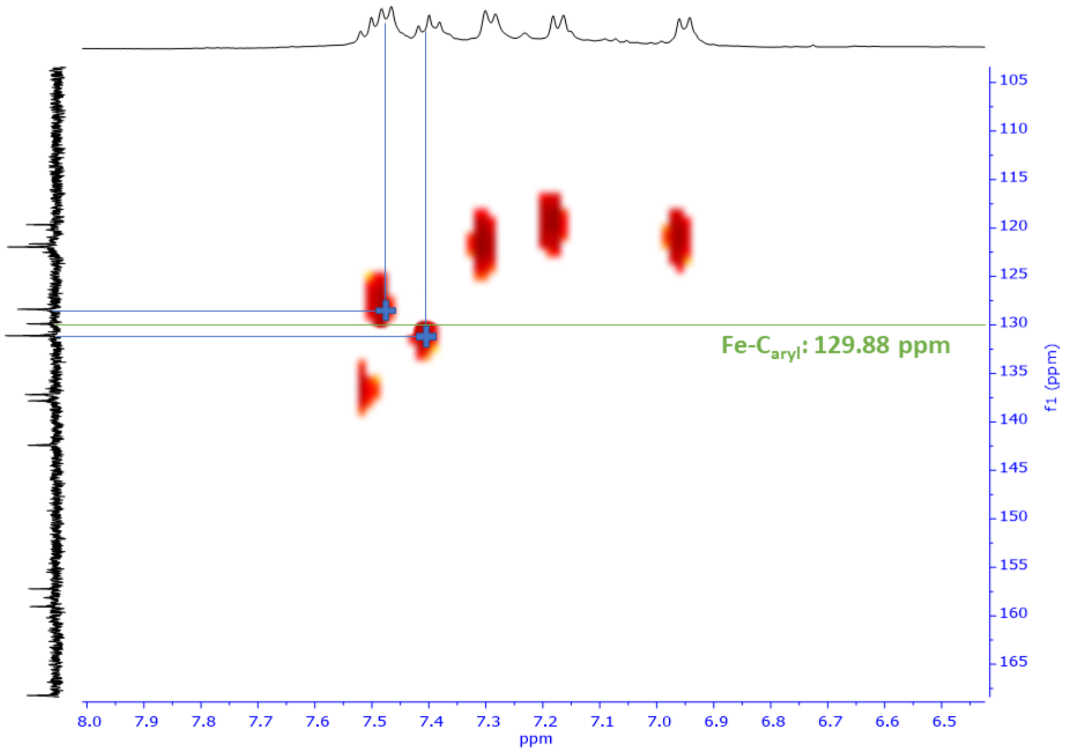

Figure S44. ^{1}H-^{13}C HSQCed spectrum of **2^{Me}(CO)** in DMSO-d_6 at room temperature (400 MHz). Ampliation of the aromatic regions.

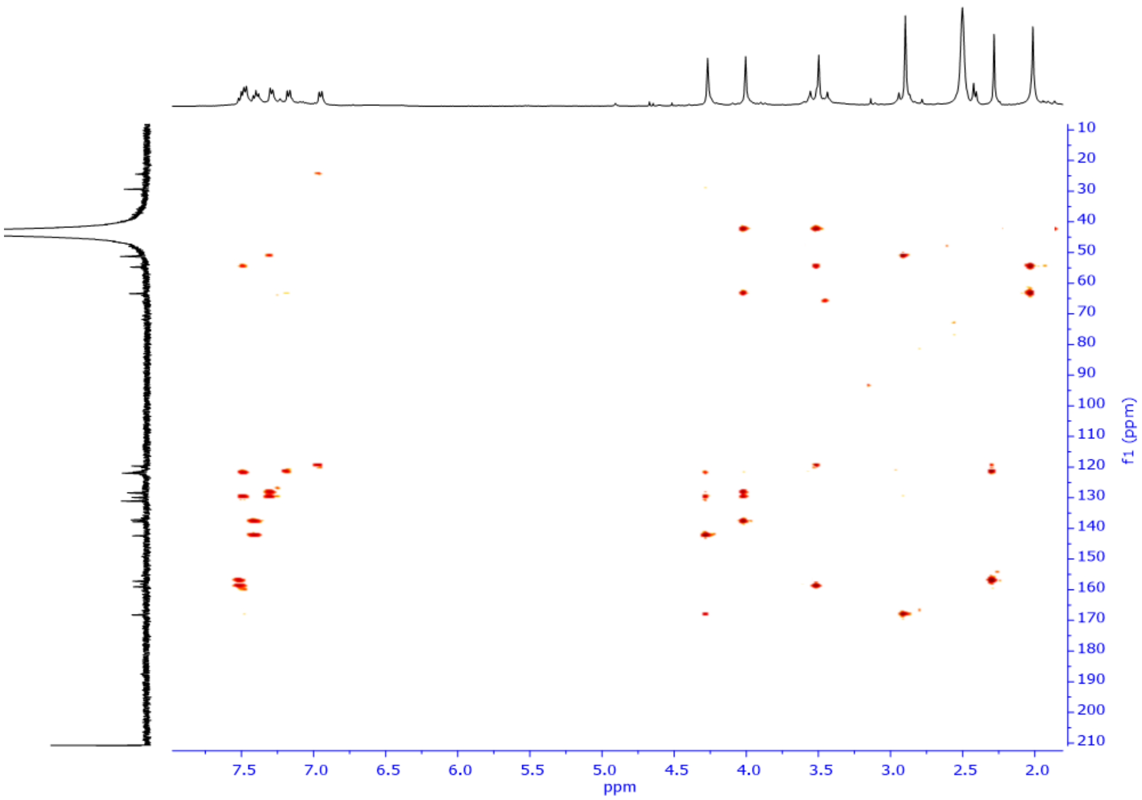

Figure S45. ^{1}H-^{13}C HMBC spectrum of **2^{Me}(CO)** in DMSO-d_6 at room temperature (400 MHz).

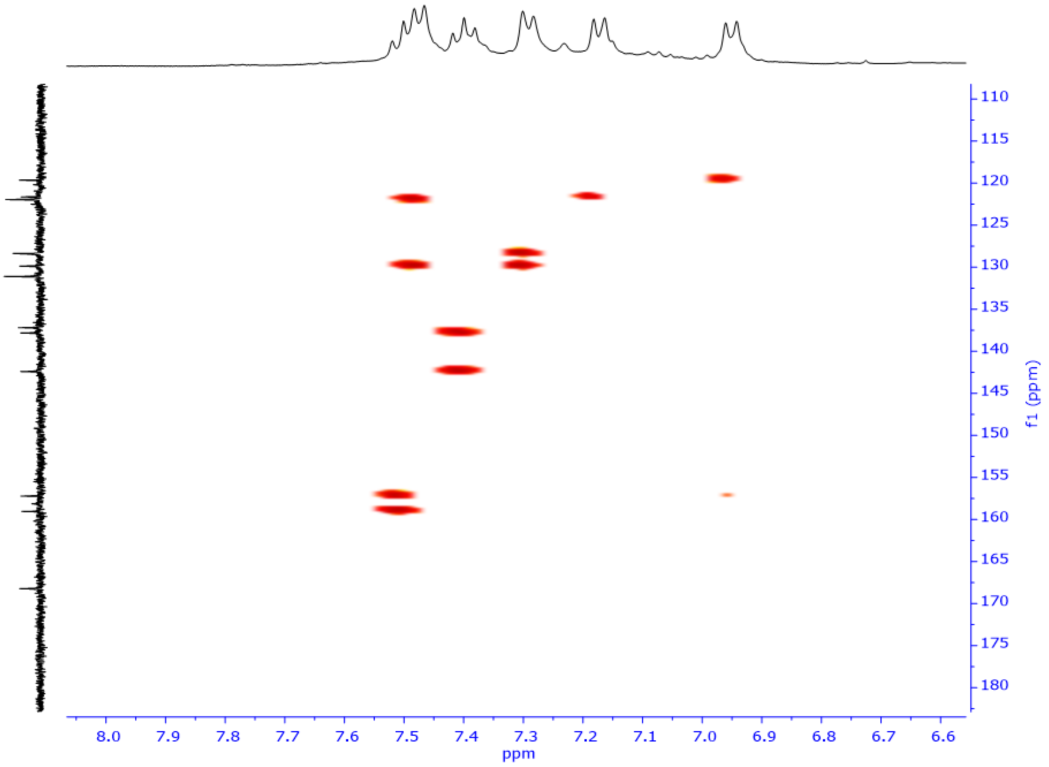

Figure S46. ^{1}H-^{13}C HMBC spectrum of **2^{Me}(CO)** in DMSO-d$_6$ at room temperature (400 MHz). Ampliation of the aromatic regions.

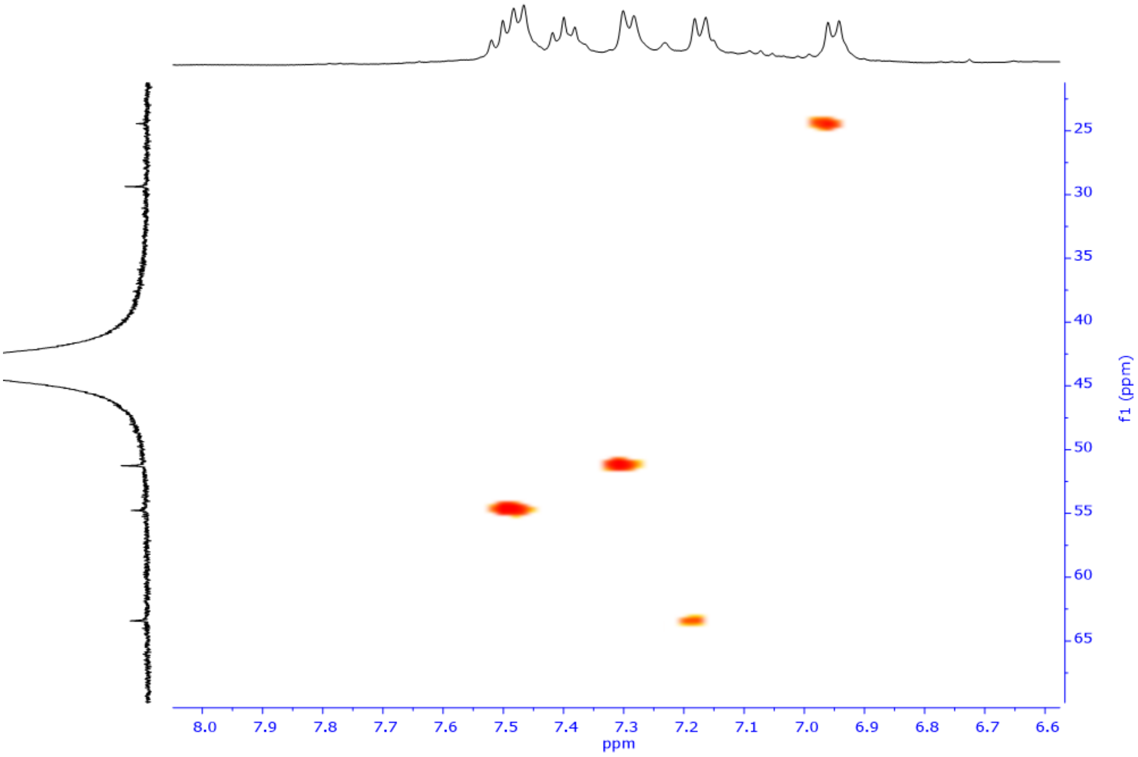

Figure S47. ^{1}H-^{13}C HMBC spectrum of **2^{Me}(CO)** in DMSO-d$_6$ at room temperature (400 MHz). Ampliation of the aromatic (^{1}H) and aliphatic (^{13}C) regions.

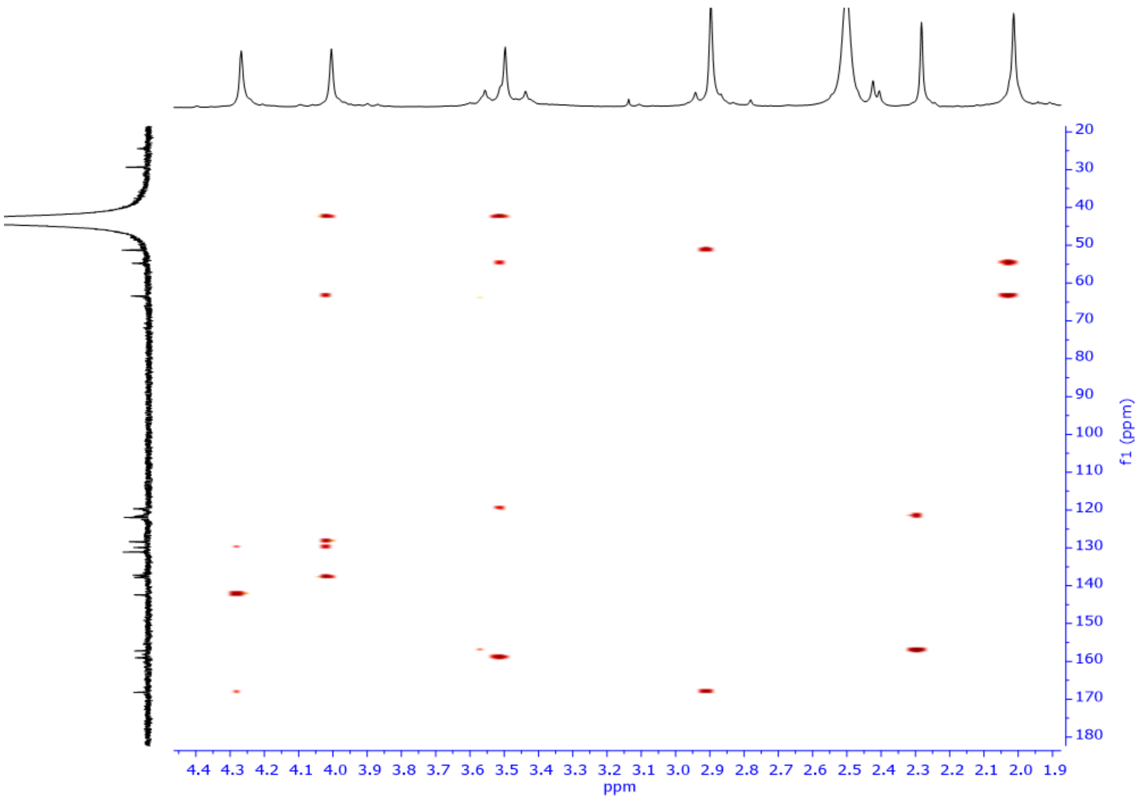

Figure S48. ^{1}H-^{13}C HMBC spectrum of **2^{Me}(CO)** in DMSO-d$_6$ at room temperature (400 MHz). Ampliation of the aliphatic (^{1}H) region vs full range (^{13}C).

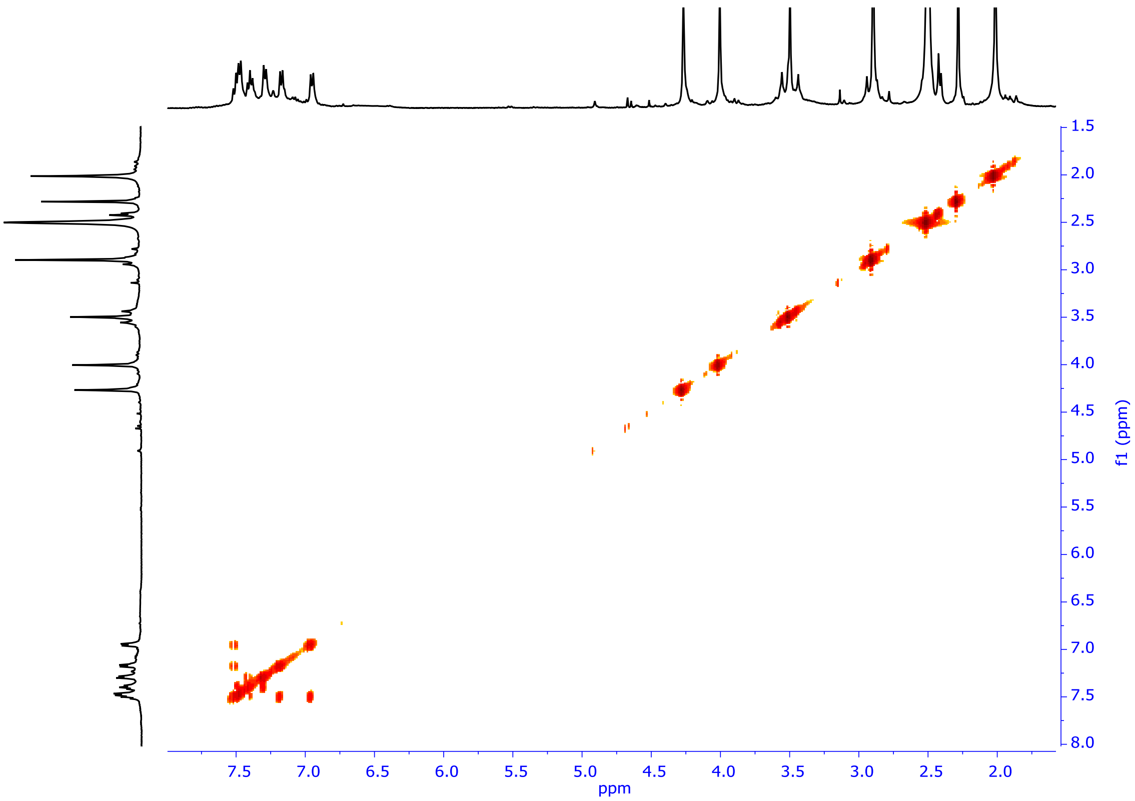

Figure S49. ^{1}H-^{1}H COSY spectrum of **2^{Me}(CO)** in DMSO-d$_6$ at room temperature (400 MHz).

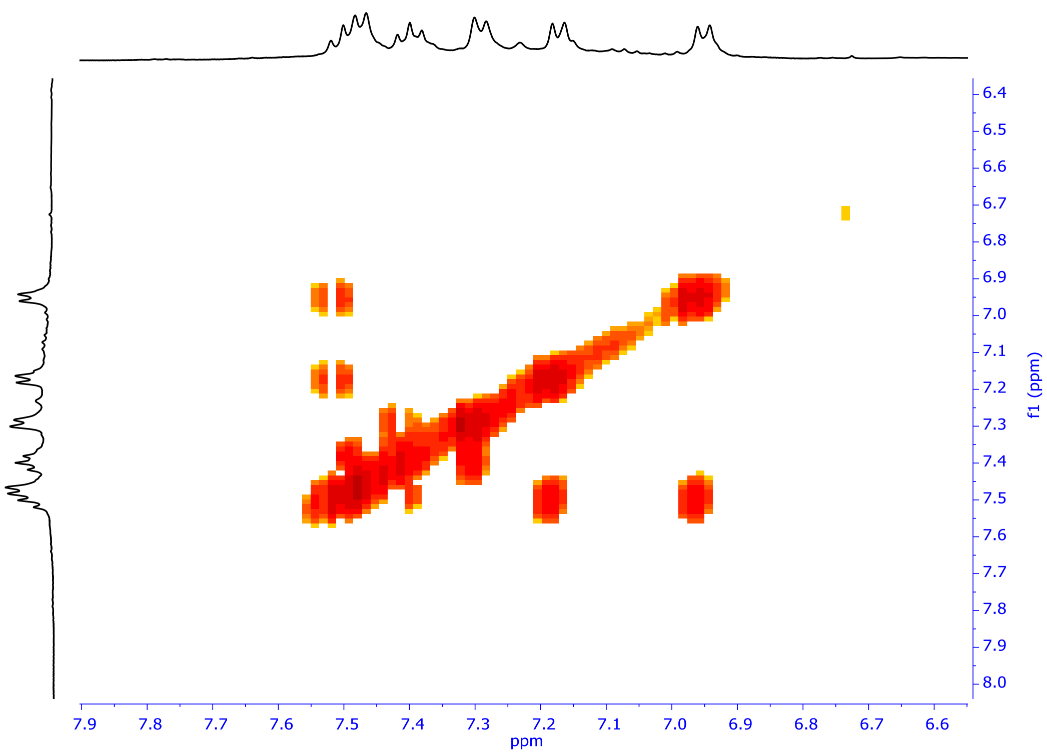

Figure S50. ^{1}H-^{1}H COSY spectrum of **2^{Me}(CO)** in DMSO-d_6 at room temperature (400 MHz). Ampliation of the aromatic region.

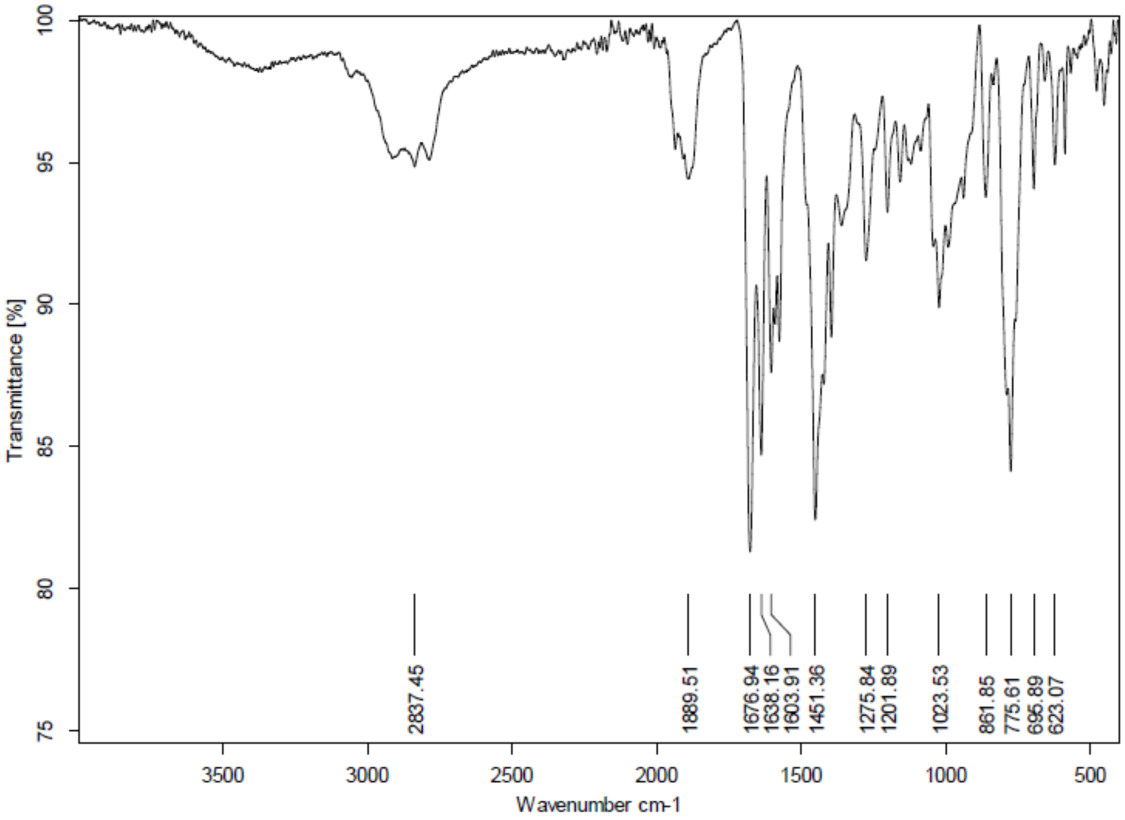

Figure S51. ATR-FT-IR spectrum of **2^{Me}(CO)** at room temperature.

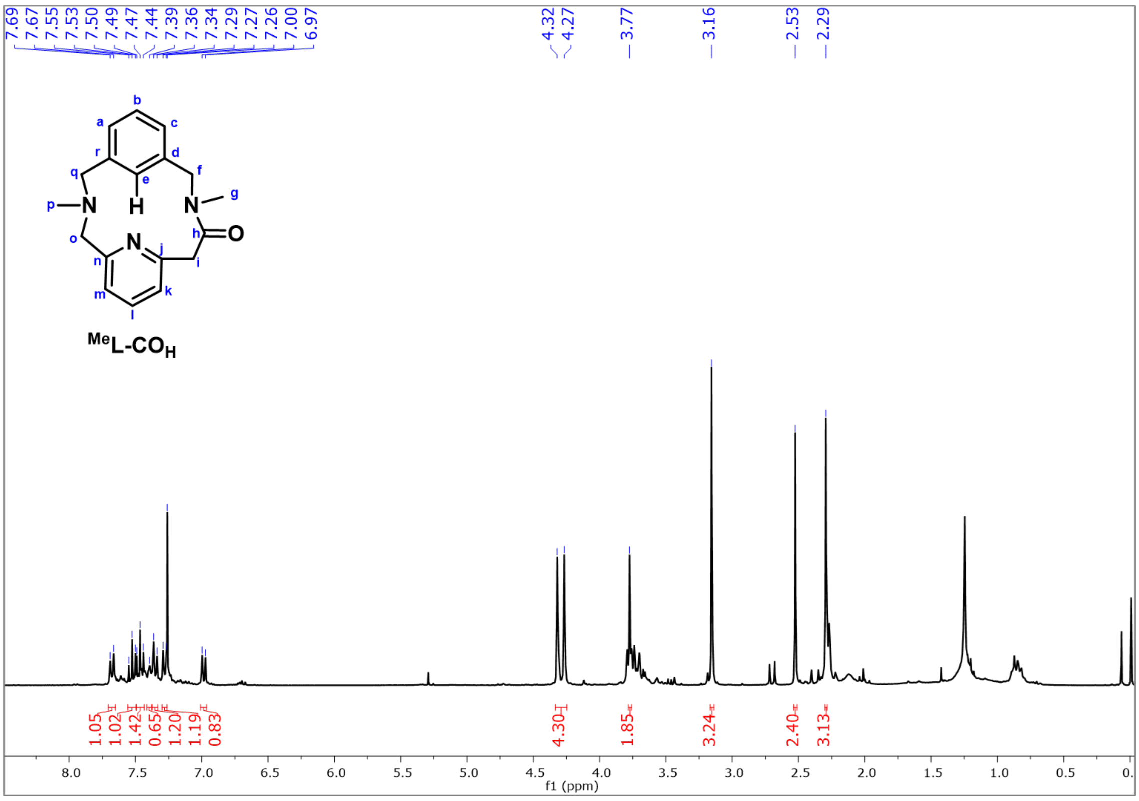

Figure S52. ^{1}H-NMR spectrum of ^{Me}**L-CO$_{H}$** in $CDCl_3$ at room temperature (400 MHz).

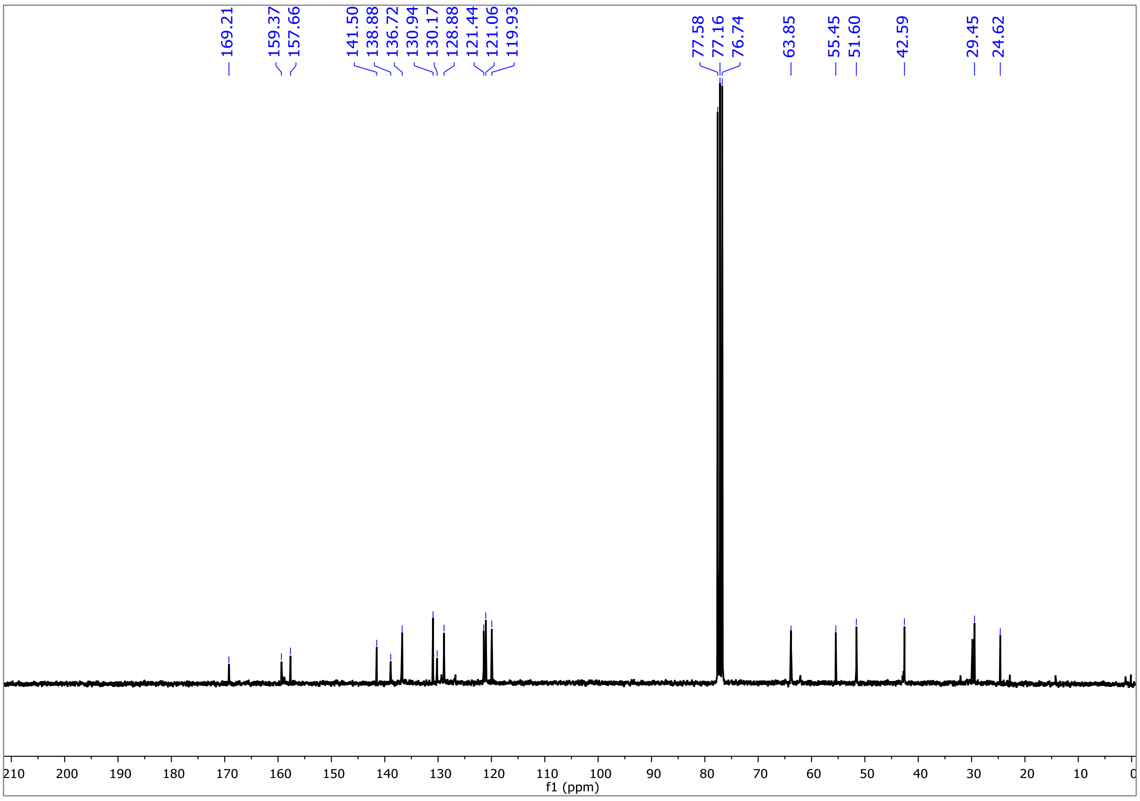

Figure S53. ^{13}C-NMR spectrum of ^{Me}**L-CO$_{H}$** in $CDCl_3$ at room temperature (400 MHz).

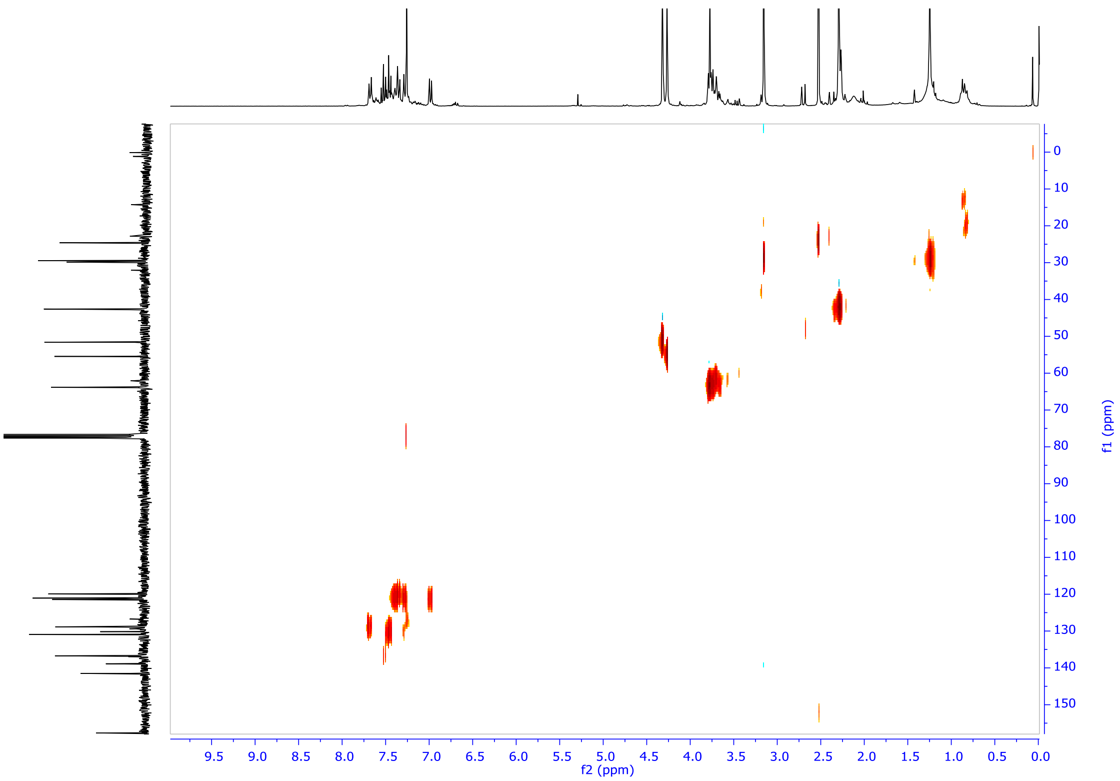

Figure S54. ^{1}H-^{13}C HSQC spectrum of ^{Me}**L-CO$_{H}$** in $CDCl_3$ at room temperature (400 MHz).

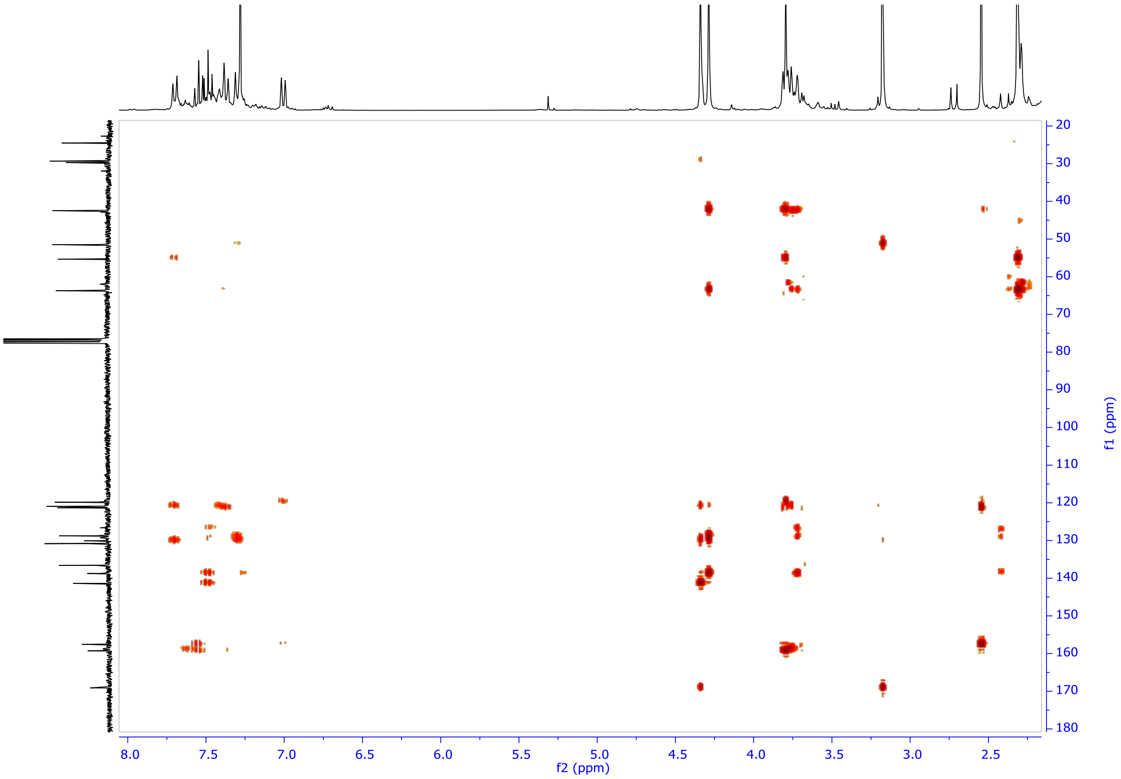

Figure S55.^{1}H-^{13}C HMBC spectrum of ^{Me}**L-CO$_{H}$** in $CDCl_3$ at room temperature (400 MHz).

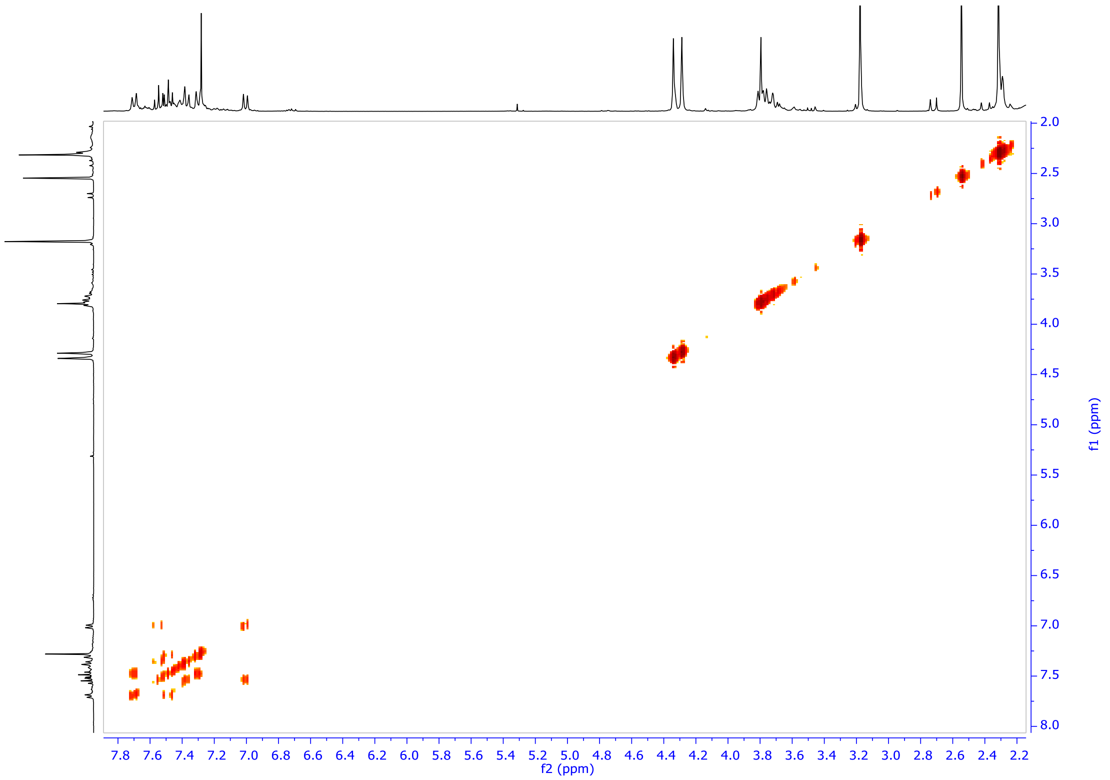

Figure S56. ^{1}H-^{1}H COSY spectrum of ^{Me}**L-CO**$_{H}$ in $CDCl_3$ at room temperature (400 MHz).

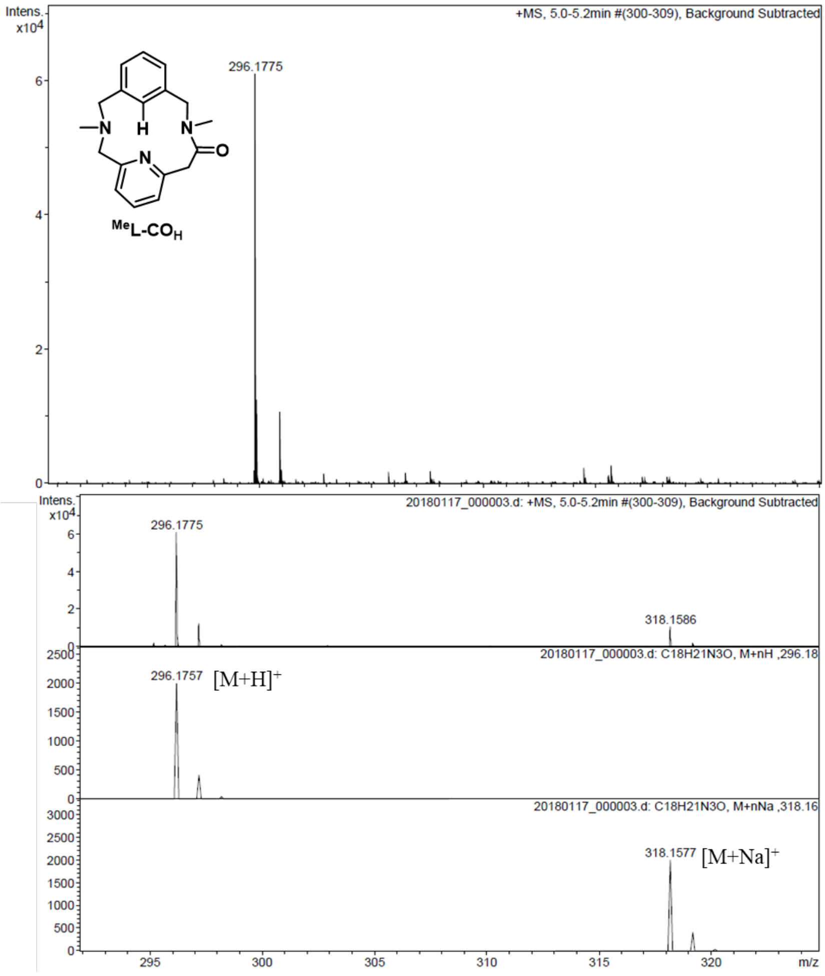

Figure S57. HR-ESI-MS of ^{Me}**L-CO$_H$**. Expanded view of the experimental peaks at m/z = 296.1775 and 318.1586 with a mass value and an isotopic pattern fully consistent with $[M+H/Na]^+$ (top) and the corresponding calculated for these molecular formulas (middle$[M+H]^+$; bottom$[M+Na]^+$). (M = $C_{18}H_{21}N_3O$).

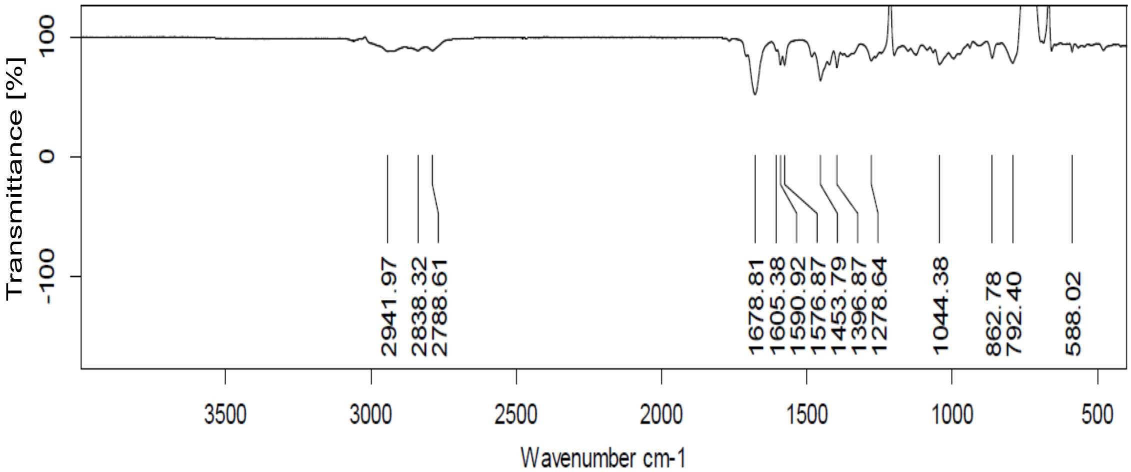

Figure S58. ATR-FT-IR spectrum of ^{Me}**L-CO$_H$** in DCM at room temperature.

10. X-ray Diffraction Analysis

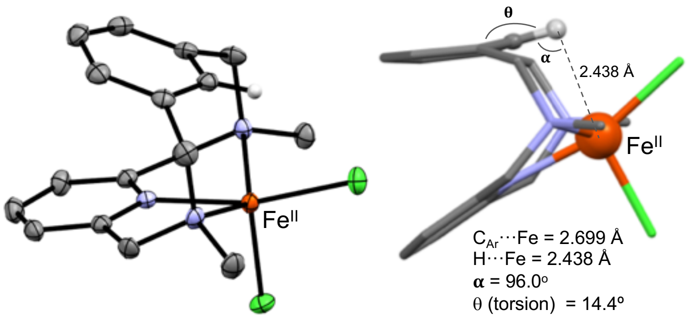

Figure S59. Crystal structure of **1·Cl$_2$** at 100 K (CCDC 2046155).

Table S1. Crystallographic parameters for **1·Cl$_2$**.

	**1·Cl$_2$**
**Chemical formula**	$C_{17}H_{21}Cl_2FeN_3$
**Formula weight**	394.12 g/mol
**Temperature**	100(2) K
**Wavelength**	0.71073 Å
**Crystal system**	orthorhombic
**Space group**	P b c a (61)
**Unit cell dimensions**	a = 12.855(2) Å α = 90°<br>b = 14.842(2) Å β = 90°<br>c = 18.151(3) Å γ = 90°
**Volume**	3463.2(9) Å^3
**Z, Density (calculated)**	8, 1.512 g/cm^3
**Absorption coefficient**	1.181 mm^{-1}
**F(000)**	1632
**Crystal size**	0.180 x 0.220 x 0.250 mm
**Theta range for data collection**	2.38 to 26.08 °
**Index ranges**	-16<=h<=17<br>-18<=k<=19<br>-24<=l<=24
**Reflections collected / Independent**	35615 / 4299<br>[R(int) = 0.0458]
**Completeness to Theta**	99.9% (Theta = 26.08°)
**Refinement method**	Full-matrix least-squares on F^2
**Data / restraints / parameters**	4299 / 0 / 210
**Goodness-of-fit on F^2**	1.075
**Final R indices**	3325 data I>2σ(I): R1 = 0.0449, wR2 = 0.0911<br>all data: R1 = 0.0635, wR2 = 0.1006
**Largest diff. peak and hole**	0.741 and -0.305 eÅ^{-3}

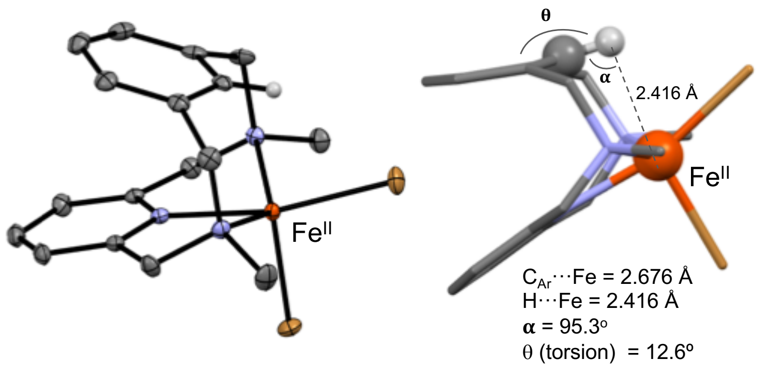

Figure S60. Crystal structure of **1·Br₂** at 100 K (CCDC 2046156).

Table S2. Crystallographic parameters for **1·Br_2**.

	**1·Br_2**	
**Chemical formula**	$C_{17}H_{21}Br_2FeN_3$	
**Formula weight**	483.04 g/mol	
**Temperature**	100(2) K	
**Wavelength**	0.71076 Å	
**Crystal system**	orthorhombic	
**Space group**	P b c a	
**Unit cell dimensions**	a = 12.927(8) Å	α = 90°
	b = 15.209(9) Å	β = 90°
	c = 18.193(12) Å	γ = 90°
**Volume**	3577(4) $Å^3$	
**Z, Density (calculated)**	8, 1.794 g/cm^3	
**Absorption coefficient**	5.313 mm^{-1}	
**F(000)**	1920	
**Crystal size**	0.090 x 0.110 x 0.220 mm	
**Theta range for data collection**	3.05 to 30.08 °	
**Index ranges**	-18<=h<=18<br>-21<=k<=12<br>-25<=l<=25	
**Reflections collected / Independent**	83185 / 5245<br>[R(int) = 0.0788]	
**Completeness to Theta**	99.9% (Theta = 30.08°)	
**Refinement method**	Full-matrix least-squares on F^2	
**Data / restraints / parameters**	5245 / 0 / 210	
**Goodness-of-fit on F^2**	1.020	
**Final R indices**	4148 data; I>2σ(I)	R1 = 0.0282, wR2 = 0.0495
	all data	R1 = 0.0471, wR2 = 0.0543
**Largest diff. peak and hole**	0.518 and -0.465 $eÅ^{-3}$	

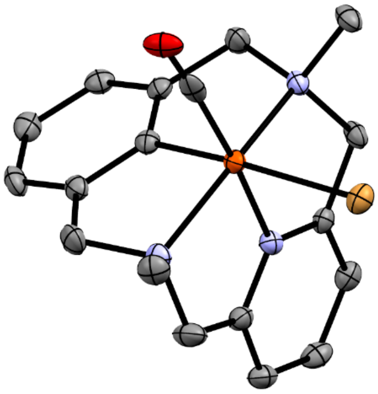

Figure S61. Crystal structure of $\mathbf{1^{Me}}$ at 100 K (CCDC 2046157).

Table S3. Crystallographic parameters for $\mathbf{1^{Me}}$.

	$\mathbf{1^{Me}}$
**Chemical formula**	$C_{18}H_{20}BrFeN_3O$
**Formula weight**	430.13 g/mol
**Temperature**	100(2) K
**Wavelength**	0.71076 Å
**Crystal system**	monoclinic
**Space group**	P 1 21/c 1
**Unit cell dimensions**	a = 14.751(14) Å α = 90°<br>b = 9.565(8) Å β = 105.17(3)°<br>c = 13.177(10) Å γ = 90°
**Volume**	1794(3) Å^3
**Z, Density (calculated)**	4, 1.592 g/cm^3
**Absorption coefficient**	3.076 mm^{-1}
**F(000)**	872
**Crystal size**	0.030 x 0.100 x 0.100 mm
**Theta range for data collection**	2.56 to 27.51 °
**Index ranges**	-19<=h<=19<br>-12<=k<=12<br>-17<=l<=17
**Reflections collected / Independent**	48846 / 4109<br>[R(int) = 0.0775]
**Completeness to Theta**	99.6% (Theta = 27.51°)
**Refinement method**	Full-matrix least-squares on F^2
**Data / restraints / parameters**	4109 / 0 / 219
**Goodness-of-fit on F^2**	1.062
**Final R indices**	3288 data; I>2σ(I) R1 = 0.0403, wR2 = 0.0805<br>all data R1 = 0.0598, wR2 = 0.0872
**Largest diff. peak and hole**	1.408 and -0.409 eÅ^{-3}

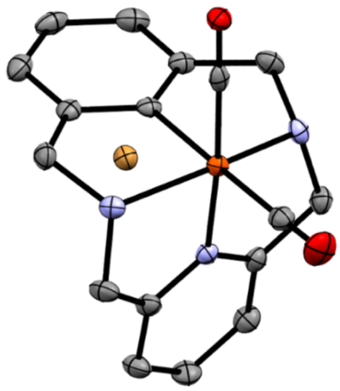

Figure S62. Crystal structure of 1^H at 100 K (CCDC 2046158).

Table S4. Crystallographic parameters for 1^H.

	1^H
**Chemical formula**	$C_{17}H_{16}BrFeN_3O_2$, CH_3CN
**Formula weight**	471.14 g/mol
**Temperature**	100(2) K
**Wavelength**	0.71076 Å
**Crystal system**	monoclinic
**Space group**	P 1 21/n 1
**Unit cell dimensions**	a = 12.717(8) Å α = 90°<br>b = 10.223(7) Å β = 97.74(3)°<br>c = 15.008(9) Å γ = 90°
**Volume**	1933(2) $Å^3$
**Z, Density (calculated)**	4, 1.619 g/cm^3
**Absorption coefficient**	2.868 mm^{-1}
**F(000)**	952
**Crystal size**	0.020 x 0.080 x 0.270 mm
**Theta range for data collection**	2.57 to 27.66 °
**Index ranges**	-16<=h<=16<br>-13<=k<=13<br>-19<=l<=19
**Reflections collected / Independent**	41971 / 4504<br>[R(int) = 0.0651]
**Completeness to Theta**	99.6% (Theta = 27.66°)
**Refinement method**	Full-matrix least-squares on F^2
**Data / restraints / parameters**	4504 / 0 / 253
**Goodness-of-fit on F^2**	1.020
**Final R indices**	3668 data; I>2σ(I) R1 = 0.0290, wR2 = 0.0545
	all data R1 = 0.0452, wR2 = 0.0591
**Largest diff. peak and hole**	0.422 and -0.346 $eÅ^{-3}$

11. DFT Modelling

11.1. Computational details

All DFT calculations were performed with the Gaussian 16 Revision A.03 program.[8] Geometry optimizations were carried out using the long-range corrected ωB97X-D functional,[9] which includes empirical dispersion correction, along with the def2-SVP basis set.[10] Solvation effects were included as a Polarizable Continuum using the SMD model.[11] Subsequently, we performed frequency calculations to each of the optimized structures to ensure that all local minima have only real frequencies and compute the Gibbs energy (ΔG), i.e., to evaluate the entropic and enthalpic corrections, assuming temperature value of 298.15 K and a pressure of 1.0 atm. Finally, single point energy calculations on the equilibrium geometries, including solvent effects, were computed with the more flexible basis set def2-TZPV.[10] Therefore, the values of ΔG reported in the manuscript are calculated at ωB97X-D/def2TZVP//ωB97X-D/def2SVP level, including solvent effects (SMD) and empirical dispersion corrections.

11.2. Relative electronic and Gibbs energy values of D, E-1 and E-2

Table S1. Relative Electronic (ΔE) and Gibbs energy (ΔG) values in kcal/mol of **D**, **E-1** and **E-2** compounds optimized at ωB97X-D/def2TZVP//ωB97X-D/def2SVP level for the spin states S = 0, 1 and 2.

Compound	ΔE	ΔG
D Singlet	0.00	1.01
D triplet	2.18	0.00
D quintuplet	22.44	14.13
E-1 Singlet	18.17	17.56
E-1 triplet	19.20	15.51
E-1 quintuplet	11.69	6.19
E-2 Singlet	36.89	34.01
E-2 triplet	26.27	22.86
E-2 quintuplet	15.47	9.72

11.3. XYZ coordinates of DFT optimized structures

All Cartesian coordinates for the optimized structures can be found in the supplementary file "Magallon_Organometallics_Cartesian_coordinates.xyz".

12. References

1. *1H-NMR spectra compared to the commercial product.*

2. Planas, O.; Whiteoak, C. J.; Martin-Diaconescu, V.; Gamba, I.; Luis, J. M.; Parella, T.; Company, A.; Ribas, X., Isolation of Key Organometallic Aryl-Co(III) Intermediates in Cobalt-Catalyzed C(sp2)–H Functionalizations and New Insights into Alkyne Annulation Reaction Mechanisms. *J. Am. Chem. Soc.* **2016,** *138,* 14388-14397.

3. Zhou, W.; Schultz, J. W.; Rath, N. P.; Mirica, L. M., Aromatic Methoxylation and Hydroxylation by Organometallic High-Valent Nickel Complexes. *J. Am. Chem. Soc.* **2015,** *137,* 7604-7607.

4. Sarbajna, A.; He, Y.-T.; Dinh, M. H.; Gladkovskaya, O.; Rahaman, S. M. W.; Karimata, A.; Khaskin, E.; Lapointe, S.; Fayzullin, R. R.; Khusnutdinova, J. R., Aryl–X Bond-Forming Reductive Elimination from High-Valent Mn–Aryl Complexes. *Organometallics* **2019,** *38,* 4409-4419.

5. Zhan, C.; Wang, X.; Wei, Z.; Evans, D. J.; Ru, X.; Zeng, X.; Liu, X., Synthesis and characterisation of polymeric materials consisting of {Fe2(CO)5}-unit and their relevance to the diiron sub-unit of [FeFe]-hydrogenase. *Dalton Trans.* **2010,** *39,* 11255-11262.

6. Ouizem, S.; Rosario-Amorin, D.; Dickie, D. A.; Paine, R. T.; de Bettencourt-Dias, A.; Hay, B. P.; Podair, J.; Delmau, L. H., Synthesis and f-element ligation properties of NCMPO-decorated pyridine N-oxide platforms. *Dalton Trans.* **2014,** *43,* 8368-8386.

7. Wessel, A. J.; Schultz, J. W.; Tang, F.; Duan, H.; Mirica, L. M., Improved synthesis of symmetrically & asymmetrically N-substituted pyridinophane derivatives. *Org. Biomol. Chem.* **2017,** *15,* 9923-9931.

8. Frisch, M. J. T., G. W.; Schlegel, H. B.; Scuseria, G. E.; Robb, M. A.; Cheeseman, J. R.; Scalmani, G.; Barone, V.; Petersson, G. A.; Nakatsuji, H.; Li, X.; Caricato, M.; Marenich, A. V.; Bloino, J.; Janesko, B. G.; Gomperts, R.; Mennucci, B.; Hratchian, H. P.; Ortiz, J. V.; Izmaylov, A. F.; Sonnenberg, J. L.; Williams-Young, D.; Ding, F.; Lipparini, F.; Egidi, F.; Goings, J.; Peng, B.; Petrone, A.; Henderson, T.; Ranasinghe, D.; Zakrzewski, V. G.; Gao, J.; Rega, N.; Zheng, G.; Liang, W.; Hada, M.; Ehara, M.; Toyota, K.; Fukuda, R.; Hasegawa, J.; Ishida, M.; Nakajima, T.; Honda, Y.; Kitao, O.; Nakai, H.; Vreven, T.; Throssell, K.; Montgomery, J. A., Jr.; Peralta, J. E.; Ogliaro, F.; Bearpark, M. J.; Heyd, J. J.; Brothers, E. N.; Kudin, K. N.; Staroverov, V. N.; Keith, T. A.; Kobayashi, R.; Normand, J.; Raghavachari, K.; Rendell, A. P.; Burant, J. C.; Iyengar, S. S.; Tomasi, J.; Cossi, M.; Millam, J. M.; Klene, M.; Adamo, C.; Cammi, R.; Ochterski, J. W.; Martin, R. L.; Morokuma, K.; Farkas, O.; Foresman, J. B.; Fox, D. J. *Gaussian 16, Revision A.03,* Gaussian Inc.: Wallingford CT, 2016.

9. Chai, J.-D.; Head-Gordon, M., Long-range corrected hybrid density functionals with damped atom–atom dispersion corrections. *Phys. Chem. Chem. Phys.* **2008,** *10,* 6615-6620.

10. Weigend, F.; Ahlrichs, R., Balanced basis sets of split valence, triple zeta valence and quadruple zeta valence quality for H to Rn: Design and assessment of accuracy. *Phys. Chem. Chem. Phys.* **2005,** *7,* 3297-3305.

11. Marenich, A. V.; Cramer, C. J.; Truhlar, D. G., Universal solvation model based on solute electron density and on a continuum model of the solvent defined by the bulk dielectric constant and atomic surface tensions. *J. Phys. Chem. B* **2009,** *113,* 6378-6396.
